# Lipid accumulation by *Coelastrella multistriata* (Scenedesmaceae, Sphaeropleales) during nitrogen and phosphorus starvation

**DOI:** 10.1038/s41598-021-99376-9

**Published:** 2021-10-06

**Authors:** Yevhen Maltsev, Zinaida Krivova, Svetlana Maltseva, Kateryna Maltseva, Elena Gorshkova, Maxim Kulikovskiy

**Affiliations:** 1 К.A. Timiryazev Institute of Plant Physiology RAS, IPP RAS, Moscow, 127276 Russia; 2 Bogdan Khmelnitsky Melitopol State Pedagogical University, Melitopol, 72312 Ukraine

**Keywords:** Phylogenetics, Plant biotechnology

## Abstract

A novel freshwater strain of *Coelastrella multistriata* MZ–Ch23 was discovered in Tula region, Russia. The identification is based on morphological features, phylogenetic analysis of SSU rDNA gene and ITS1–5.8S rDNA–ITS2 region and predicted secondary structure of the ITS2. Phylogenetic analysis places the novel strain in the “core” *Coelastrella* clade within the Chlorophyceae. This is the first record of *Coelastrella multistriata* in the algal flora of Russia. Cultivation experiments were carried out to evaluate growth dynamics of the newly identified strain and the impact of nitrogen and/or phosphorus depletion on the fatty acid profiles and lipid productivity. On the fully supplemented Bold’s basal medium and under phosphorus-depleted conditions as well, the fatty acid profiles were dominated by α-linolenic acid (29.4–38.1% of total fatty acids). Depletion of either nitrogen or both nitrogen and phosphorus was associated with increased content of oleic acid (32.9–33.7%) and linoleic acid (11.9%). Prolongation of the growth to two months (instead of 25 days) resulted in increased content and diversity of very long-chain fatty acids including saturated species. The total very long-chain fatty acid content of 9.99% achieved in these experiments was 1.9–12.3-fold higher than in stress experiments. The highest variation was observed for oleic acid (3.4–33.7%). The novel strain showed the ability to accumulate lipids in amounts up to 639.8 mg L^−1^ under nitrogen and phosphorus starvation, which exceeds the previously obtained values for most *Coelastrella* strains. Thus, the newly identified MZ–Ch23 strain can be considered as a potential producer of omega-3 fatty acids on fully supplemented Bold’s basal medium or as a source of biomass with high content of saturated and monounsaturated fatty acids after nitrogen and phosphorus starvation.

## Introduction

The green microalgae genus *Coelastrella* Chodat (Scenedesmaceae, Sphaeropleales) is characterized by highly variable morphology, which complicates species identification^1–3^. DNA sequencing often provides the only means to determine taxonomic status of algae with *Coelastrella*-like phenotypes. The use of lipid profiles as algal biomarkers^4,5^ indicates their possible taxonomic utility complementing the methods of light microscopy and molecular phylogeny.

*Coelastrella* species have a wide range of biotechnological uses. Certain strains of *Coelastrella* combine the ability to accumulate secondary carotenoids (up to 2% dry weight, DW) and lipids (up to 50% DW) with high resistance to various stresses^6–8^. Experiments were carried out to study the effects of light intensity^9^, supplementation with inorganic or organic carbon against the low-pH stress background^8^, nutrient composition of the media^6,10^, the two-stage mixotrophic/heterotrophic cultivation^11^, and simultaneous changes in illumination intensity and growth temperature^12^ on *Coelastrella* biochemistry. It has been shown that *Coelastrella*’s total cellular lipids can be converted into high quality biodiesel^10,12,13^ including a variety that complies with the EN14214 standard^8^. At the same time, *Coelastrella* represents a source of omega-3 polyunsaturated fatty acids which constitute 19.2–59.4% of its total fatty acid content^4,7,9,10,12^. The great demand for polyunsaturated fatty acids, notably the long-chain omega-3 species (eicosapentaenoic, docosahexaenoic, and α-linolenic), is due to their relevance to human health, with multiple neutralizing functions and pharmacological actions^14^. Arachidonic, docosahexaenoic, eicosapentaenoic, and γ-linolenic acids are important structural components of cell membranes and precursors of eicosanoids, e.g. the biologically active prostaglandins and leukotrienes^15^. Accordingly, new wild algae strains capable of synthesizing high amounts of omega-3 fatty acids are much sought after. Optimized protocols of their cultivation will contribute to balanced diets for humans, particularly by provision of feeds and supplements for agriculture and aquaculture.

Fine changes in algal cell morphology, physiology, and biochemistry, notably the fatty acid content, often represent adaptations to the action of extreme environmental factors^16^. Algae are among the first organisms to colonize anthropogenically disturbed habitats, thanks to their extreme ecological plasticity and ability to withstand the negative impact of extreme environmental conditions^17–19^. Studying the diversity of algae that populate anthropogenically disturbed lands in Tula region, we isolated a new strain of *Coelastrella* from mineral waste (young dump of a granite quarry). The identification involved a complex of morphological, molecular, and biochemical methods. To assess biotechnological potential of the new strain, experiments were carried out to determine the optimal cultivation conditions and variability of the fatty acid profiles as a function of growth medium composition.

## Results

### Taxonomic affiliation of the strain

**Figure 1:**
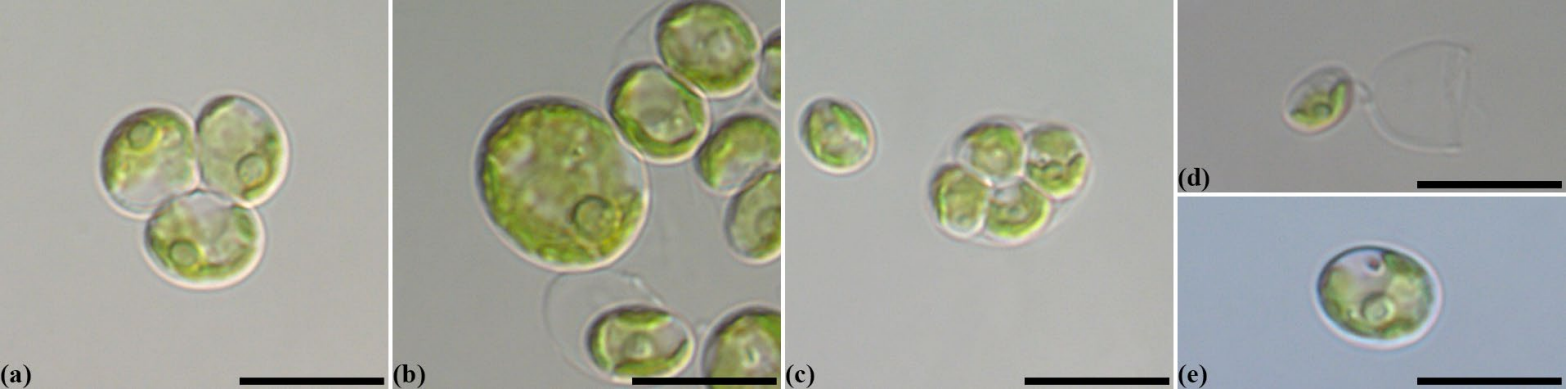
Nomarski interference micrographs for *Coelastrella multistriata* MZ–Ch23 in culture. Scale bar = 10 μm. (**a**) A group of cells in 2-weeks old culture. (**b**) Mature vegetative cell in 4-weeks old culture. (**c**) Autosporangium. (**d**) An autospore beside the mother cell wall remnant. (**e**) Mature cell staining with 0.1% methylene blue, note the absence of mucilage.

*Coelastrella multistriata* MZ–Ch23 is a green unicellular coccoid soil microalga (Fig. 1). The cells are solitary or form groups. Mature cells are ellipsoidal, 10–12 μm in length and 6–8 μm in width, rarely spherical 6.0–14.0 (15.0) μm in diameter. The cell wall is thin and colorless; the chloroplast is parietal, cup-shaped, and often dissected into blades as the cells age. Pyrenoid is single with a sheath of several large starch grains. The strain propagates by autospores. Autospores ellipsoidal or oblong-ovoid, 4–6 × 6–8 μm in size, enclosed 2, 4, or 8 per sporangium. The autospores are released by mother cell wall rupture. No sexual reproduction has been observed.

Sequence data: partial 18S rDNA gene sequence comprising V4 domain sequence (GenBank accession number MZ620281) and the ITS1–5.8S rDNA–ITS2 rDNA sequence (GenBank accession number MZ620280) for the strain MZ–Ch23.

Molecular analysis: According to the modern taxonomy of Chlorophyta, *C*. *multistriata* (Trenkwalder) Kalina et Puncochárová belongs to Scenedesmaceae family within Sphaeropleales^3^. As demonstrated by a number of previous studies, some strains belonging to different Scenedesmaceae species may have identical 18S rDNA sequences; the examples include *Scotiellopsis reticulata* Puncocharova et Kalina, *Scenedesmus rubescens* (P.J.L. Dangeard) Kessler et al.^1^, and *Scenedesmus rotundus* Wood^20^, as well as *Coelastrella tenuitheca* Qinghua Wang, Huiyin Song, Xudong Liu, Guoxing Liu et Zhengyu Hu and *Asterarcys quadricellulare* (K. Behre) Hegewald and Schmidt^3^. The reliability of phylogenetic analysis for Scenedesmaceae can be increased by involving additional genomic sequences. Internal transcribed spacer 2 (ITS2) is a rapidly evolving rDNA segment of the nuclear operon. ITS2 sequencing has been successfully applied to determine phylogenetic status of systematically ambiguous taxa at the species and subspecies levels^21,22^. In this study, the reconstruction of phylogenetic relationships was carried out using the Maximum Likelihood (ML) and Bayesian inference (BI) methods.

**Figure 2:**
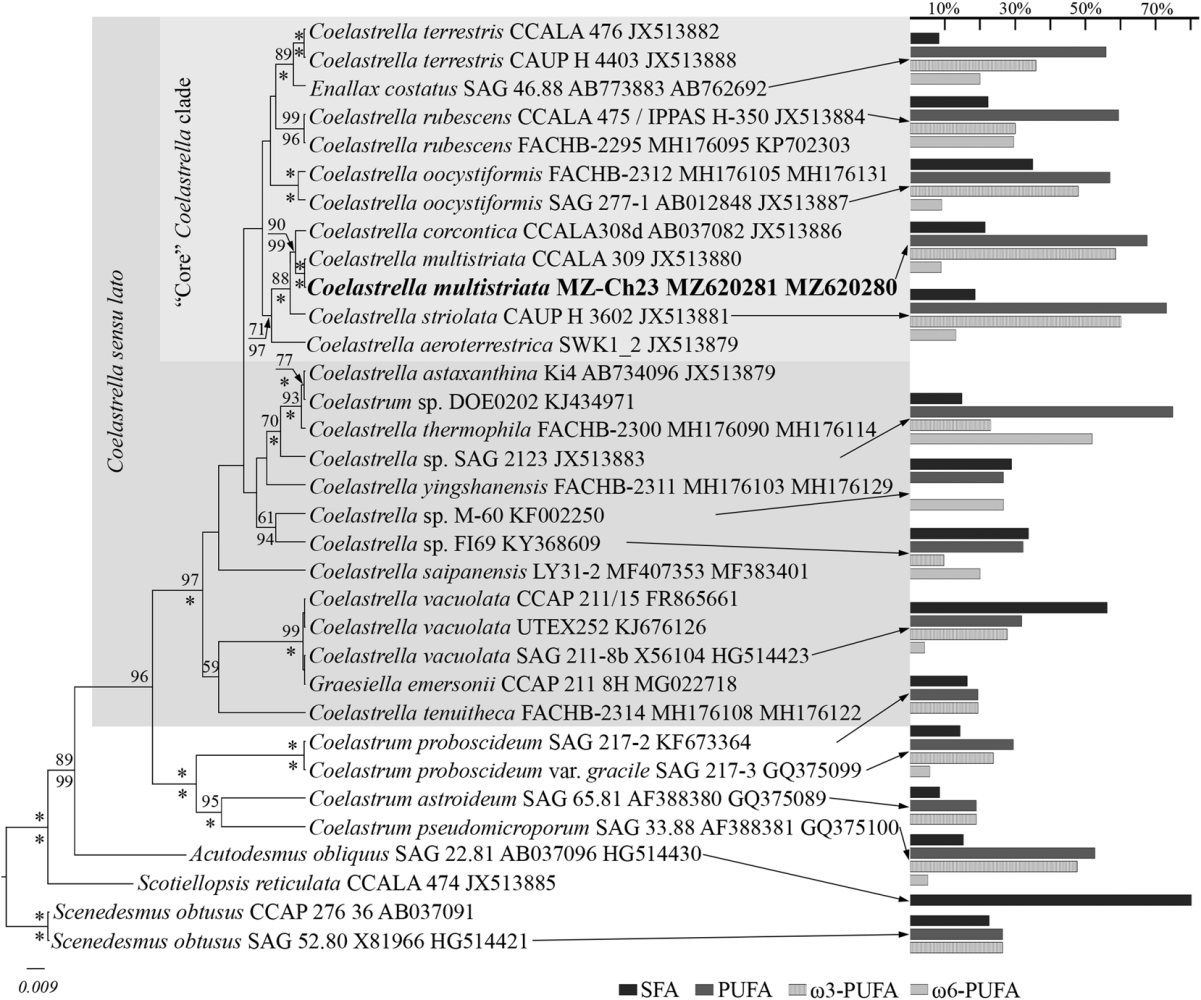
Phylogenetic position of *Coelastrella multistriata* MZ–Ch23 (indicated in bold) within the Sphaeropleales based on Bayesian inference for the partial 18S rDNA gene and ITS1–5.8S rDNA–ITS2 region. Total length of the alignment is 1118 characters. Values above the horizontal lines are bootstrap support from RAxML analyses (< 50 are not shown); values below the horizontal lines are Bayesian posterior probabilities (< 90 are not shown); the asterisk * indicates 100% statistical support. Strain numbers (if available) and GenBank numbers are indicated for all sequences. Clades are designated according to Wang et al.^3^. The content of ω3-PUFAs, ω6-PUFAs, saturated and polyunsaturated fatty acids (% of total fatty acids) of Sphaeropleales species cultivated in fully supplemented media, from this study (printed in bold) and published works^4,7,10,12^ is indicated on the right.

Consistently with previous studies on *Coelastrella* phylogeny, the analysis identified *Coelastrella* sensu lato clade (Fig. 2) encompassing *Coelastrella*, *Enallax* Pascher, and *Graesiella* T. Kalina et M. Puncochárová genera^1–3^. Terminal part of the tree corresponded to the “core” *Coelastrella* subclade comprising the type species *Coelastrella* (*C*. *striolata* Chodat, *C*. *corcontica* (T. Kalina et M. Puncochárová) E. Hegewald et N. Hanagata, *C*. *multistriata*) and *Scotiellopsis* Vinatzer (*S*. *rubescens* = *Coelastrella rubescens* (Vinatzer) Kaufnerová et Eliás) which formed independent branches within the subclade. The new strain MZ–Ch23 was included with high statistical support: likelihood bootstrap (LB) 88, posterior probability (PP) 100 in the original *Coelastrella* group (Fig. 2) and with maximum support (LB 100, PP 100) in a sibling branch comprising the authentic *C*. *multistriata* CCALA 309 strain.

**Figure 3:**
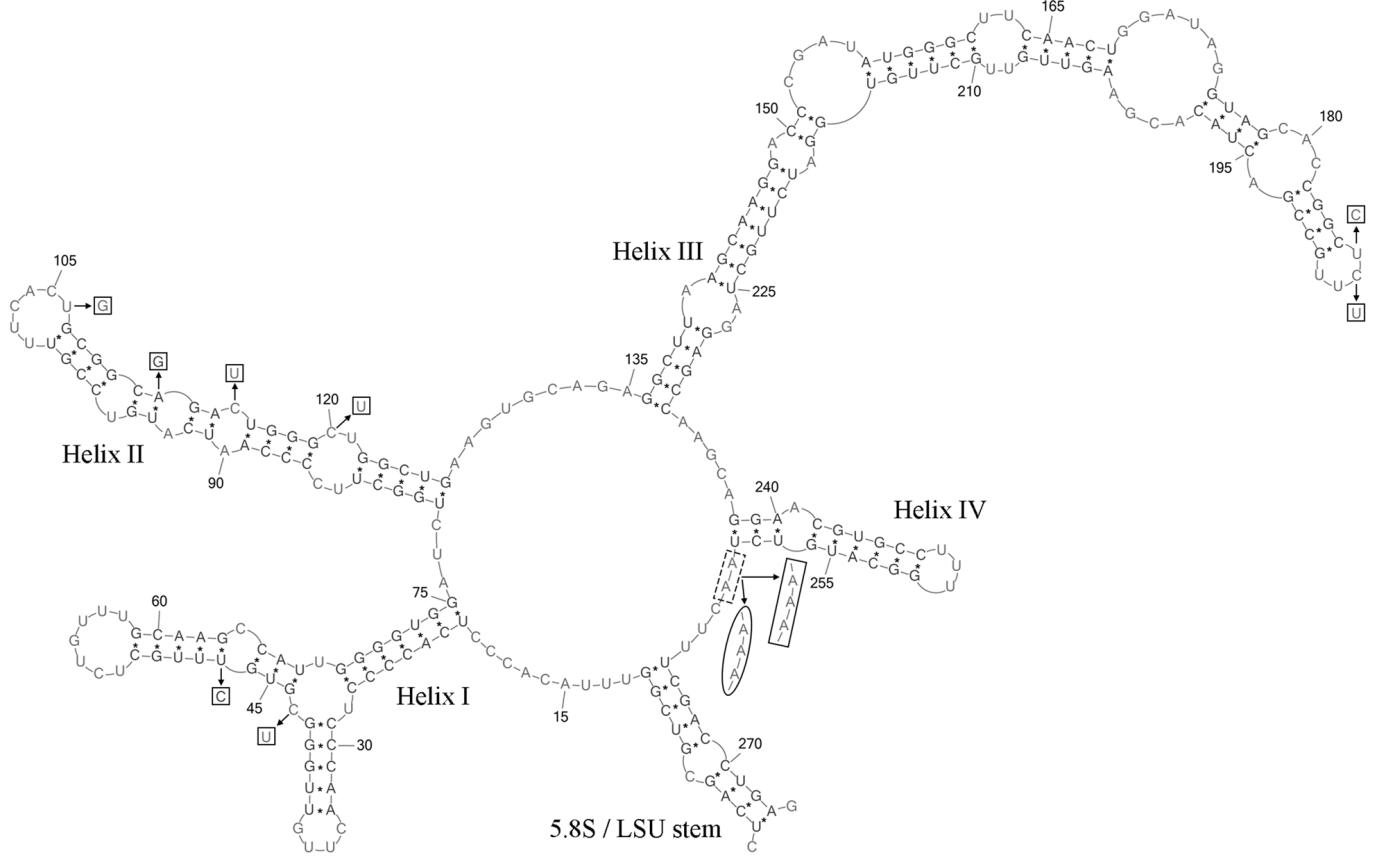
Predicted secondary structure of the ITS2 for *Coelastrella multistriata* MZ–Ch23. Base numbering is indicated every 15 bases. The four helices are numbered with Roman numerals. Nucleotide positions distinctive for *Coelastrella striolata* CAUP H 3602 and *Coelastrella multistriata* CCALA 309 are shown outside the secondary structure. Deletions, single bases and hCBCs for CAUP H 3602 are shown by pentagons. Deletion for CCALA 309 is shown by ellipse.

Comparative analysis of ITS2 sequences for the newly identified MZ–Ch23 *vs C*. *multistriata* CCALA 309 and *C*. *striolata* CAUP H 3602 revealed a number of evolutionary events that occurred in this group. The MZ–Ch23 and *C*. *multistriata* CCALA 309 sequences differed by a single-nucleotide A deletion in the central loop posterior to helix IV (Fig. 3). The MZ–Ch23 and *C*. *striolata* CAUP H 3602 sequences differed by one C → U transition and one hemi-compensatory base change (hCBC) U–G → C–G in helix I, one U → G transversion at the end of helix II, one hCBC U–G → U–A and two C → U transitions in the conserved part of helix II, C → U and U → C transitions at the end of helix III, and a single-nucleotide A deletion in the central loop posterior to helix IV. Thus, the newly identified strain MZ–Ch23 revealed the highest evolutionary affinity to *C*. *multistriata* CCALA 309.

### Growth parameters

**Figure 4:**
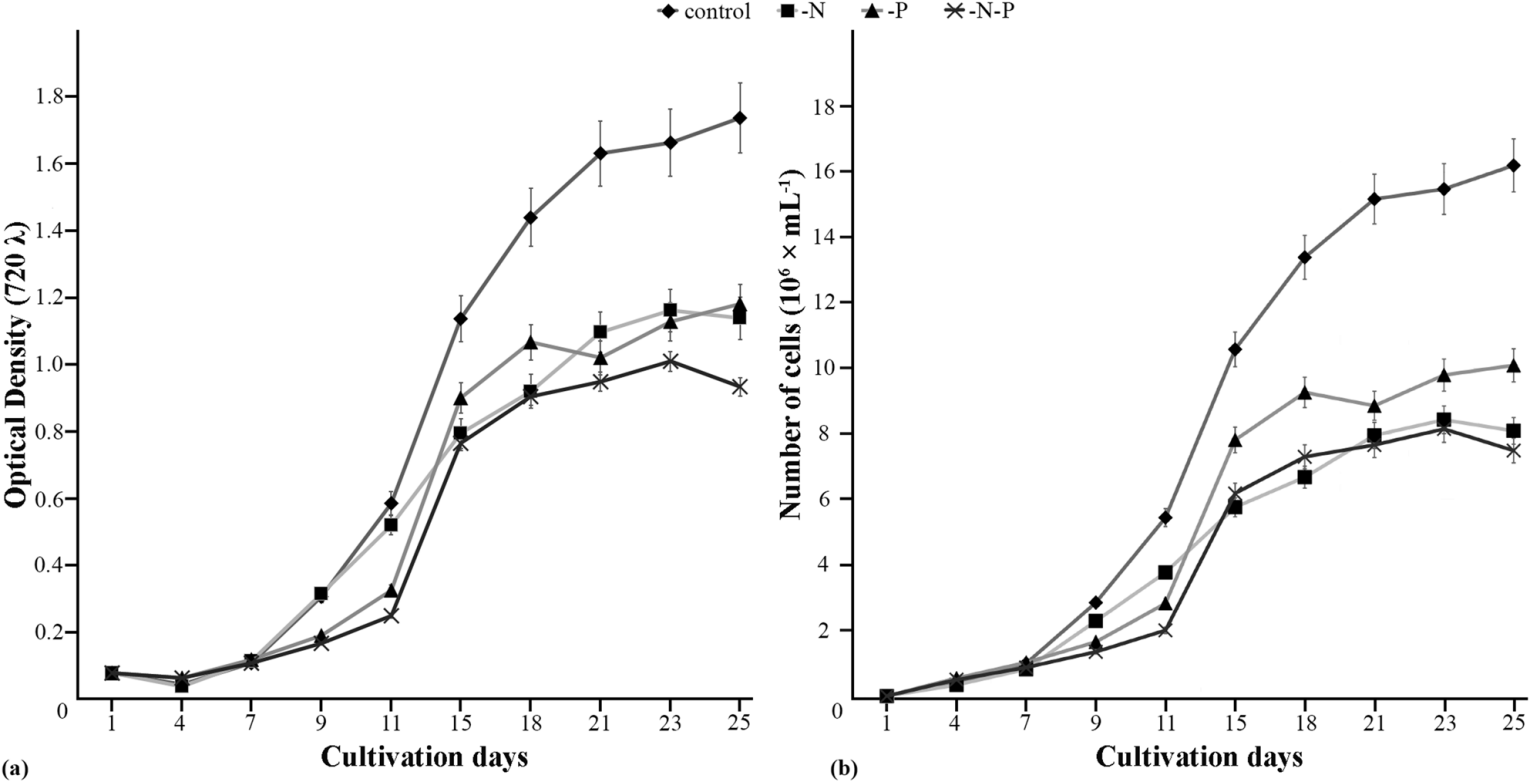
Growth curves of *Coelastrella multistriata* MZ–Ch23 under different cultivation conditions. (**a**) Optical density, arithmetic means ± standard errors (S.E.), n = 3. (**b**) Cell concentration, arithmetic means ± standard errors (S.E.), n = 3.

The newly isolated strain MZ–Ch23 of *C. multistriata* was maintained continuously on Bold’s basal medium (BBM). The effects of nitrogen and/or phosphorus depletion on the biomass production and biochemical parameters of the cultures were studied experimentally using BBM depleted of nitrogen (-N), BBM depleted of phosphorus (-P), and BBM depleted of nitrogen and phosphorus (-N-P), with fully supplemented BBM as the control. The growth rates were assessed by OD_720_ measurements and algal cell counts. The growth curves showed typical sigmoid character for all cultures (Fig. 4).

Lag phase was observed during the initial seven days of cultivation for all media (Fig. 4). After day 7, all cultures entered exponential phase with a steady increase in optical density till day 21 under control conditions or day 18 under the mineral depletion stress. The cultures subsequently slowed down their growth and entered stationary phase, during which OD_720_ and cell counts remained almost constant. Starting from day 11, OD_720_ was significantly lower for all nutrient-depleted formulations compared with the control (Fig. 4a); the lowest values were observed in the experiment with combined depletion of nitrogen and phosphorus (-N-P). The observed character of biomass accumulation by the strain MZ–Ch23 suggests that the growth of *C. multistriata* was significantly inhibited by depletion of nitrogen and phosphorus. It should be noted that prolonged cultivation (for 60 days instead of 25) provided no significant increase in *C. multistriata* culture optical density (Table 1).

**Table 1:** Characteristics of the strain *Coelastrella multistriata* MZ–Ch23 under different cultivation conditions, the data are reported as the mean ± standard error from three independent biological replicates.

	60 days culture	control	-N	-P	-N-P
Biomass dry weight (g L^−1^)	1.39^A^ ± 0.04	1.30^AB^ ± 0.03	1.09^A^ ± 0.02	0.90^AB^ ± 0.02	1.12^B^ ± 0.04
Total lipids (% of biomass)	35.40^AB^ ± 2.43	27.0^AB^ ± 3.64	45.90^A^ ± 1.67	37.10^C^ ± 2.75	57.20^B^ ± 3.21
Total lipids (mg L^−1^)	491.10^AB^ ± 27.23	349.90^A^ ± 26.39	501.50^AB^ ± 23.85	335.30^B^ ± 7.36	639.80^AB^ ± 19.88
Total fatty acids (% of biomass)	38.80^A^ ± 0.71	27.20^AB^ ± 0.93	47.70^AB^ ± 1.72	39.90^B^ ± 0.82	58.50^AB^ ± 0.97
Total fatty acids (mg L^−1^)	538.20^A^ ± 24.2	352.50^AB^ ± 13.8	521.10^B^ ± 23.40	360.60^AB^ ± 15.90	654.30^AB^ ± 33.90
TAGs (% of biomass)	9.30^AB^ ± 0.29	8.40^AB^ ± 0.21	11.70^AB^ ± 0.34	14.50^A^ ± 0.23	14.30^B^ ± 0.32
TAGs (mg L^−1^)	129.0^A^ ± 2.6	108.90^ABC^ ± 2.56	127.80^B^ ± 2.83	131.10^C^ ± 2.33	159.90^ABC^ ± 3.17
Cell number (10^6^ mL^−1^)	11.60^AB^ ± 0.35	16.20^AB^ ± 0.43	8.10^A^ ± 0.31	10.10^AB^ ± 0.24	7.50^B^ ± 0.17

Identical superscript letters indicate that means are significantly different.

^A–D^Means within the same line are significantly different with *P* ≤ 0.01.

The highest dry weight yields of biomass of *C. multistriata* on day 25 were obtained with the control medium (fully supplemented BBM containing 2.94 mM nitrates and 1.72 mM phosphates, Table 1). These yields were only slightly lower than after 60 days of cultivation (Table 1). With either phosphorus or nitrogen depletion, the DW yields of biomass decreased by 30.27% and 15.70%, respectively. With simultaneous depletion of nitrogen and phosphorus (-N-P), the DW yield was 1118.50 ± 36.47 mg L^–1^, 13.69% lower than control values. The differences between the control and nitrogen-depleted conditions (*F*_4,10_ = 6.97, *P* = 0.006), as well as phosphorus-depleted and simultaneous nitrogen and phosphorus depleted conditions (*F*_4,10_ = 7.82, *P* = 0.004), were statistically significant. The non-significant difference in growth rate between (-N) and (-N-P) cultures suggests that depletion of nitrogen promote insensitivity to the shortage of phosphorus (Fig. 4).

### Total lipid and triacylglyceride content

In fully supplemented control cultures, total lipid content was the lowest 27.0 ± 3.64% of DW (Table 1). Depletion of nitrogen from the growth medium was associated with a 1.7-fold increase in the total lipid content, further promoted to a 2.1-fold by simultaneous depletion of nitrogen and phosphorus. Meanwhile, depletion of phosphorus alone had minimal effect on the lipid content. In total values, despite the stress-associated growth inhibition, amounts of accumulated lipids reached their maximum under -N-P conditions. The yield of 639.8 ± 19.88 mg L^–1^ achieved under (-N-P) conditions was 1.83-fold higher compared with the control and 1.91-fold higher than under (-P) conditions. Statistically significant differences in the total lipid content were associated with nitrogen depletion: *F*_4,10_ = 7.82, *P* = 0.004 for (-N) *vs* control; *F*_4,10_ = 11.28, *P* = 0.001 for (-N-P) *vs* control and (-N-P) *vs* P; and *F*_4,10_ = 9.43, *P* = 0.002 for (-N) *vs* (-P). The difference in total lipid content between phosphorus-depleted cultures (-P) and the control was non-significant (*F*_4,10_ = 0.26, *P* = 0.899).

Consistently, the lowest content of triacylglycerides (TAGs) was observed in fully supplemented control cultures. TAG content of *C. multistriata* biomass of increased significantly under (-P) and (-N-P) conditions 1.73-fold and 1.70-fold, respectively, compared with the control (Table 1). The highest total yields of TAGs were obtained in (-N-P) medium 159.9 ± 3.17 mg L^−1^, 1.47-fold higher compared with the control. Statistical differences in TAG yields were significant for all pairs except between the phosphorus-depleted medium (-P) and combined nitrogen and phosphorus starvation (-N-P) (*F*_4,10_ = 0.26, *P* = 0.899).

### Fatty acid profiles

Fatty acid profiles of the newly described *C. multistriata* MZ–Ch23 were assessed after cultivation on BBM under nitrogen and/or phosphorus depletion (-N, -P, -N-P) or without it (control). In the fully supplemented medium, the dominant fatty acids were α-linolenic 18:3 (38.12 ± 0.62% of the total fatty acid content corresponding to the total yield of 134.4 mg L^−1^), hexadecatrienoic 16:3 (19.61 ± 2.13%, 69.0 mg L^−1^), palmitic 16:0 (18.61 ± 1.4%, 65.7 mg L^−1^), and oleic 18:1 (8.05 ± 0.69%, 28.2 mg L^−1^). Depletion of phosphorus (-P) promoted an increase in the content of palmitic 16:0 and oleic 18:1 acids (23.63 ± 1.09% and 13.29 ± 0.81%, respectively, Table 2), while the content of α-linolenic 18:3 acid decreased almost 1.3-fold. Under nitrogen depletion (-N), palmitic acid content remained almost the same as in the control (17.86 ± 2.04%), while oleic 18:1 acid content increased significantly (32.93 ± 1.53%, *F*_4,10_ = 11.29, *P* = 0.001 compared with the control). Also, under (-N) conditions, the content of α-linolenic 18:3 acid showed a significant 2.17-fold decrease, whereas the content of linoleic 18:2 acid increased significantly to 11.94 ± 0.72% (1.63-fold compared with the control). Altogether, the stress conditions prevented accumulation of hexadecatrienoic 16:3 acid, its concentration decreasing significantly from at least 1.53-fold on (-P) medium to 3.98-fold on (-N) medium. The strongest effects of the mineral depletion stress were observed for oleic 18:1 acid. The highest content of oleic acid, 33.71% of total fatty acids, observed in (-N-P) medium, was 3.1–5.7-fold higher than corresponding values obtained for other *Coelastrella* species under various cultivation conditions^4,7,10,12^.

**Table 2:** Fatty acid composition of the strain *Coelastrella multistriata* MZ–Ch23 under different cultivation conditions, the data are reported as the mean (% of total fatty acids) ± standard error from three independent biological replicates.

Acids	60 days culture	control	-N	-P	-N-P
12:0 Lauric acid	0.43 ± 0.01	*	*	*	*
14:0 Myristic acid	1.21^A^ ± 0.05	0.25^A^ ± 0.02	*	*	*
16:0 Palmitic acid	20.38^A^ ± 1.29	18.61^B^ ± 1.40	17.86^C^ ± 2.04	23.63^D^ ± 1.09	18.98^E^ ± 1.74
16:1n-9 *cis*-7-Hexadecenoic acid	1.08 ± 0.12	*	*	*	*
16:1n-7 *cis*-9-Palmitoleic acid	2.37^k^ ± 0.46	1.63^m^ ± 0.19	*	2.57^l^ ± 0.16	1.20^kl^ ± 0.08
16:2n-6 *cis*-7,10-Hexadecadienoic acid	3.60^AB^ ± 0.06	1.49^ACD^ ± 0.11	4.35^CE^ ± 0.40	1.87^BEF^ ± 0.17	3.81^DF^ ± 0.27
16:3n-3 *cis*-7,10,13-Hexadecatrienoic acid	8.96^A^ ± 0.72	19.61^ABCk^ ± 2.13	4.93^BD^ ± 0.60	12.84^Dkl^ ± 1.17	6.77^Cl^ ± 0.65
16:4n-3 *cis*-4,7,10,13-Hexadecatrienoic acid	*	0.26 ± 0.03	*	*	*
18:0 Stearic acid	2.88^kl^ ± 0.04	1.49^BDm^ ± 0.16	5.17^Bk^ ± 0.52	3.79^m^ ± 0.25	4.76^Dl^ ± 0.47
18:1n-9 *cis*-9-Oleic acid	3.41^AB^ ± 0.33	8.05^CDk^ ± 0.69	32.93^AC^ ± 1.53	13.29^AEk^ ± 0.81	33.71^BDE^ ± 1.52
18:1n-7 *cis*-11-Vaccenic acid	2.92^A^ ± 0.37	2.36^B^ ± 0.31	*	2.35^C^ ± 0.26	*
18:2n-6 *cis*-9,12-Linoleic acid	9.19^A^ ± 1.10	7.32^B^ ± 0.99	11.94^C^ ± 1.47	8.10^D^ ± 0.96	10.29^E^ ± 1.39
18:3n-3 *cis*-9,12,15-*α*-Linolenic acid	33.58^ABkl^ ± 1.25	38.12^CDk^ ± 0.62	17.56^AC^ ± 0.57	29.38^CEl^ ± 1.08	18.04^BDE^ ± 0.70
20:0 Arachidic acid	0.48^AB^ ± 0.06	*	1.45^A^ ± 0.16	*	1.06^B^ ± 0.08
20:1n-7 *cis*-13-Eicosenoic acid	*	*	0.32 ± 0.05	*	*
20:3n-6 *cis*-8,11,14-Dihomo-γ-linolenic acid	0.15 ± 0.01	*	*	*	*
20:5n-3 *cis*-5,8,11,14,17-Eicosapentaenoic acid	0.28 ± 0.02	*	*	*	*
21:0 Heneicosanoic acid	*	0.54 ± 0.03	*	*	*
22:0 Behenic acid	1.03^Ak^ ± 0.05	0.27^AB^ ± 0.03	2.00^AC^ ± 0.08	*	0.75^BCk^ ± 0.05
24:0 Lignoceric acid	3.67^AB^ ± 0.19	*	1.49^Ak^ ± 0.11	0.93^Bk^ ± 0.06	0.63^A^ ± 0.05
26:0 Cerotic acid	3.87^A^ ± 0.13	*	*	1.25^A^ ± 0.04	*
28:0 Montanic acid	0.51 ± 0.03	*	*	*	*
ΣSFAs	34.46^A^ ± 2.12	21.16^A^ ± 1.62	27.97^B^ ± 2.88	29.60^C^ ± 1.43	26.18^D^ ± 2.38
ΣMUFAs	9.78^ABk^ ± 1.27	12.04^CD^ ± 1.19	33.25^ACE^ ± 1.58	18.21^EFk^ ± 1.23	34.91^BDF^ ± 1.60
ΣSFAs + MUFAs	44.24^kl^ ± 3.39	33.20^AB^ ± 2.80	61.22^Ak^ ± 4.44	47.81^C^ ± 2.66	61.09^Bl^ ± 3.93
ΣPUFAs	55.76^kl^ ± 3.39	66.80^AB^ ± 2.80	38.78^Ak^ ± 4.44	52.19^C^ ± 2.66	38.91^Bl^ ± 3.93

The asterisk * indicates “not registered”, MUFA, monounsaturated fatty acid; PUFA, polyunsaturated fatty acid; SFA, saturated fatty acid.

Identical superscript letters indicate that means are significantly different.

^A–F^ Means within the same line are significantly different with *P* ≤ 0.01.

^k–m^ Means within the same line are significantly different with *P* ≤ 0.05.

Comparison of the MZ–Ch23 biomass collected immediately after transition to the stationary phase (day 25) with longer term cultivation (day 60) shows considerable differences in the fatty acid profiles. After 60 days on fully supplemented BBM, the biomass had lower content of hexadecatrienoic 16:3 and oleic 18:1 fatty acids compared with the 25 days-old cultures. At the same time, the 60 days-old cultures contained the highest diversity of very long-chain fatty acids (VLCFAs) including saturated species. The content of VLCFAs after 60 days cultures was 9.99% of total fatty acids, which is 1.9–12.3-fold higher than corresponding values obtained in the 25-days stress experiments. In the 60 days-old cultures, the highest proportions of VLCFAs were constituted by lignoceric 24:0 and cerotic 26:0 acids (Tables 2 and 3).

**Table 3:** Fatty acid content of the strain *Coelastrella multistriata* MZ–Ch23 under different cultivation conditions, the data are reported as the mean (mg L^−1^) ± standard error from three independent biological replicates.

Acids	60 days culture	control	-N	-P	-N-P
12:0 Lauric acid	2.2 ± 0.1	*	*	*	*
14:0 Myristic acid	6.5^A^ ± 0.2	0.9^A^ ± 0.3	*	*	*
16:0 Palmitic acid	109.7^k^ ± 1.9	65.7^Ak^ ± 3.3	93.0^B^ ± 5.1	85.2^l^ ± 6.3	124.2^Al^ ± 15.0
16:1n-9 *cis*-7-Hexadecenoic acid	5.8 ± 0.1	*	*	*	*
16:1n-7 *cis*-9-Palmitoleic acid	12.8^A^ ± 0.3	5.7^A^ ± 0.9	*	9.3^B^ ± 1.8	7.8^C^ ± 0.9
16:2n-6 *cis*-7,10-Hexadecadienoic acid	19.4^A^ ± 1.1	5.4^ABC^ ± 0.6	22.8^BD^ ± 3.6	6.9^DE^ ± 0.6	24.9^CE^ ± 3.3
16:3n-3 *cis*-7,10,13-Hexadecatrienoic acid	48.2^kl^ ± 1.7	69.0^ABCk^ ± 3.1	25.8^Almn^ ± 3.9	46.2^Bm^ ± 4.5	44.4^Cn^ ± 4.5
16:4n-3 *cis*-4,7,10,13-Hexadecatrienoic acid	*	0.9 ± 0.3	*	*	*
18:0 Stearic acid	15.5^Akl^ ± 0.3	5.4^BCk^ ± 0.9	27.0^BDl^ ± 2.7	13.8^DE^ ± 1.8	31.2^ACE^ ± 3.1
18:1n-9 *cis*-9-Oleic acid	18.4^AB^ ± 0.9	28.2^CD^ ± 3.6	171.3^ACE^ ± 18.9	48.0^EF^ ± 4.5	220.5^BDF^ ± 27.9
18:1n-7 *cis*-11-Vaccenic acid	15.7^AB^ ± 0.2	8.4^A^ ± 1.5	*	8.4^B^ ± 2.1	*
18:2n-6 *cis*-9,12-Linoleic acid	49.5^k^ ± 1.5	25.8^ABk^ ± 3.6	62.1^AC^ ± 5.4	29.1^CD^ ± 4.5	67.5^BD^ ± 6.9
18:3n-3 *cis*-9,12,15-*α*-Linolenic acid	180.7^ABC^ ± 5.4	134.4^A^ ± 10.2	91.5^A^ ± 4.8	105.9^B^ ± 5.7	117.9^C^ ± 5.4
20:0 Arachidic acid	2.6^AB^ ± 0.1	*	7.5^A^ ± 0.6	*	6.9^B^ ± 0.9
20:1n-7 *cis*-13-Eicosenoic acid	*	*	1.8 ± 0.3	*	*
20:3n-6 *cis*-8,11,14-Dihomo-γ-linolenic acid	0.8 ± 0.1	*	*	*	*
20:5n-3 *cis*-5,8,11,14,17-Eicosapentaenoic acid	1.5 ± 0.1	*	*	*	*
21:0 Heneicosanoic acid	*	1.8 ± 0.3	*	*	*
22:0 Behenic acid	5.5^A^ ± 0.1	0.9^AB^ ± 0.3	10.5^AC^ ± 1.2	*	4.8^BC^ ± 0.6
24:0 Lignoceric acid	19.8^ABC^ ± 1.6	*	7.8^Ak^ ± 0.9	3.3^Bk^ ± 0.3	4.2^C^ ± 0.3
26:0 Cerotic acid	20.8^A^ ± 1.2	*	*	4.5^A^ ± 0.3	*
28:0 Montanic acid	2.8 ± 0.1	*	*	*	*
ΣSFAs	185.5^AB^ ± 5.2	74.7^ACD^ ± 4.8	145.8^C^ ± 10.2	106.8^Bk^ ± 8.4	171.3^Dk^ ± 19.2
ΣMUFAs	52.6^AB^ ± 1.6	42.3^CD^ ± 5.7	173.1^ACE^ ± 19.2	65.7^EF^ ± 8.4	228.3^BDF^ ± 28.8
ΣSFAs + MUFAs	238.1^Akl^ ± 6.7	117.0^Bk^ ± 10.5	318.9^Cl^ ± 29.1	172.5^D^ ± 16.5	399.6^ABCD^ ± 47.7
ΣPUFAs	300.1^Ak^ ± 9.7	235.5^B^ ± 17.1	202.2^k^ ± 17.4	188.1^A^ ± 15.3	254.7^C^ ± 19.8

The asterisk * indicates “not registered”. Identical superscript letters indicate that means are significantly different.

^A–F^Means within the same line are significantly different with *P* ≤ 0.01.

^k–n^Means within the same line are significantly different with *P* ≤ 0.05.

The highest content of saturated fatty acids (29.60% of the total fatty acid content of the biomass) was observed under depletion of phosphorus (-P). Simultaneous depletion of nitrogen and phosphorus (-N-P) enriched *C. multistriata* MZ–Ch23 biomass with monounsaturated fatty acids (34.91% of total fatty acids). The highest content of polyunsaturated fatty acids (66.80%) was observed in fully supplemented BBM. The highest total content of saturated and monounsaturated fatty acids, amid sufficiently rapid growth, was observed in nitrogen-depleted cultures: 61.22 ± 4.44% for (-N) and 61.09 ± 3.93% for (-N-P). The highest total yield of polyunsaturated fatty acids (254.7 mg L^–1^) was observed under nitrogen-and-phosphorus depletion (-N-P). The same (-N-P) medium provided the highest total yield of saturated and monounsaturated fatty acids (399.6 mg L^−1^, 3.4-fold as much as in the control).

## Discussion

Morphological features of the isolated strain MZ–Ch23 correspond to the established morphotype of *Coelastrella*: single (occasionally grouped) ovoid and ellipsoidal cells with clearly visible cell walls without polar thickenings, comprising single parietal lobular chloroplast with single pyrenoid in a starch sheath. More detailed morphological examination suggests identification of the MZ–Ch23 strain with *C. multistriata*, which is distinct from other known species of *Coelastrella* by ellipsoidal shape (compared to the rounded cells of *C. rubescens*), specific size range of vegetative cells (6.0–15.0 μm in diameter, compared to 10.0–16.0 μm in *C. thermophila* Qinghua Wang, Huiyin Song, Xudong Liu, Guoxiang Liu et Zhengyu Hu, 12.0–18.0 µm in *C. yingshanensis* Qinghua Wang, Huiyin Song, Xudong Liu, Guoxiang Liu et Zhengyu Hu, and 5.0–10.0 µm in *C. astaxanthina* K. Ohkoshi, R. Yoshida et S. Kawasaki), and the lack of apical thickening (compared to its observable presence in *C. oocystiformis* (J.W.G. Lund) E. Hegewald et N. Hanagata, *C. terrestris* (Reisigl) Hegewald et N. Hanagata, and *C. rubescens*). The new MZ–Ch23 strain is distinguished by the absence of meridional ribs on the cell surface, revealed by light microscopy. The ML and BI phylogenetic analysis of V4 region of the 18S rDNA gene and the downstream ITS1–5.8S rRNA–ITS2 region associates MZ–Ch23 with strain *C. multistriata* CCALA 309 with maximum statistical support (LB 100, PP 100; Fig. 2). Its strong relationship with other strains of *Coelastrella* sensu lato (LB 97, PP 100), notably strains of the “core” *Coelastrella* clade, is evident as well. Thus, the results of morphological analysis, molecular phylogeny investigation, and comparative analysis of the ITS2 secondary structure unanimously identify the discovered strain MZ–Ch23 with *C. multistriata*.

*Coelastrella* species are widespread in European ecosystems: *C. rubescens* is ubiquitous, and *C. terrestris* and *C. oocystiformis* are frequent. These are mostly terrestrial species also found in temporary water bodies. *C. striolata* is noted less frequently, predominantly as a benthic and periphytic species in sphagnum bogs, or occasionally in overgrown ponds and soil^23^. *Coelastrella* species found outside Europe include terrestrial *C. astaxanthina*, described from Japan^2^ and *C. ellipsoidea* (P.M. Novis et G. Visnovksy) K. Gopalakrishnan, P.M. Novis et G. Visnovsky, known from New Zealand^24^. Among the freshwater planktonic species *C. yingshanensis* from China^3^ and *C. saipanensis* N. Hanagata from Japan^25^ are known. *C. tenuitheca* and *C. thermophila* are quite common, characterized simultaneously as subaerophytic, terrestrial, and hydrophytic^3^. *C. multistriata*, classified as a terrestrial species, has been originally isolated from the soils of Italian Alps and Germany^26^. Its finding closest to Russia was on granite surfaces and in its cracks of the Teteriv river canyon in Zhytomyr region, Ukraine^27^. To the best of our knowledge (based on published evidence), the strain we isolated from a young granite carrier dump in Tula region represents the first record of *C. multistriata* in the algal flora of Russia.

The previous experience of different *Coelastrella* strains cultivation shows their ability to produce biomass in the range of (0.78) 1.10–1.85 (3.0) g L^–1^. The lowest yields of biomass were recorded for a *Coelastrella* sp. strain grown for 7 days on BG11 medium under nitrogen and phosphorus depletion^11^, whereas the highest yields were recorded for *C. striolata* var. *multistriata* cultured in a slender test tube for 30 days on Bold’s basal medium with 5.88 mM NaNO_3_ at 65 μmol photons m^–2^ s^–1^^6^. Consequently, the ability of MZ–Ch23 to produce biomass within the range of 0.9–1.39 g L^–1^ is inherent to most *Coelastrella* strains, including *C. rubescens* IPPAS H-350 (= CCALA 475)^7,8^ and *Coelastrella* sp.^11^.

Content of synthesized lipids in algal biomass is an important indicator of biotechnological utility. The discovered strain *C. multistriata* MZ–Ch23 accumulates lipids in amounts of up to 57.2% of its DW biomass (639.8 mg L^–1^ under nitrogen-and-phosphorus depleted conditions), which exceeds the previously obtained values for most *Coelastrella* strains. A high lipid content recorded for strain *C. rubescens* IPPAS H-350 amounted to 57% of its biomass under conditions of increased acidity of the medium: Bold’s basal medium acidified to pH 5^7^, optionally with continuous air sparging^8^. A high content of lipids (40% of DW biomass) was obtained with combined mixotrophic and heterotrophic cultivation of *Coelastrella* sp. under nitrogen and phosphorus depletion^11^, and a similar value (37% of DW biomass) was obtained upon cultivation of *Coelastrella* sp. M-60 on BG11 medium under salt stress^10^. However, all of these high lipid concentrations were obtained at lower biomass production values; consequently, the total yields of lipids per liter of culture volume were significantly lower than corresponding values obtained by us for strain MZ–Ch23, which highlights its biotechnological potential.

For different strains of *Coelastrella*, fatty acid repertoires of cultures grown under standard conditions are quite similar^4,7,9,10,12^ (Fig. 2). In most strains, 7,10-hexadecadienoic (1.5–8.4%), palmitic (6.3–19.6%), 7,10,13-hexadecatrienoic (8.1–26.9%), stearic (1.3–28.6%), oleic (6.0–31.4%), linoleic (4.1–41.8%), and α-linolenic (6.9–44.2%) acids are dominant. Compared to other *Coelastrella* strains, *C. multistriata* MZ–Ch23 shows enrichment with 7,10,13-hexadecatrienoic acid (19.61% on fully supplemented medium) and oleic acid (32.93% under nitrogen depletion).

The content of 7,10,13-hexadecatrienoic acid in the MZ–Ch23 biomass obtained on fully supplemented Bold’s basal medium 19.61% of total fatty acids is considerably higher (up to 22.5%) than has been reported for strain *C. striolata* CAUP H 3602 (15.20%). In addition, the strain MZ–Ch23 contains oleic acid at a level of 8.05% under normal conditions boosting to 13.29–32.93% under stress, in striking contrast to the strain CAUP H 3602 containing oleic acid in negligible quantities^4^. The same strain *C. striolata* CAUP H 3602, however, accumulates remarkably high amounts of vaccenic (9.30% of total fatty acids), linoleic (12.90%), and α-linolenic (44.20%) acids, cf. the maxima of 2.36% for 18:1 (cis-∆11), 11.94% for 18:2 (under nitrogen depletion), and 38.12% for 18:3, respectively, in MZ–Ch23. At the same time, MZ–Ch23 and CAUP H 3602 accumulate similar amounts of the saturated palmitic acid: 17.86–23.63% and 18.40% of total fatty acids, respectively. The 9.99% VLCFA content of MZ–Ch23 strain approaches the maximum among the analyzed *Coelastrella* strains; the only higher value (11.70% of total fatty acids) was obtained for *Coelastrella* sp. M-60 on BG11 medium^10^.

Depletion of nitrogen and/or phosphorus from the culture medium significantly alters the contributions of particular fatty acids to the fatty acid profiles of the strain MZ–Ch23. For instance, nitrogen starvation boosted the content of oleic and linoleic acids, while promoting a decrease in the content of 7,10,13-hexadecatrienoic and α-linolenic acids, while phosphorus starvation enriched the cultures with palmitic acid (Table 2).

Various effects of stress on fatty acid profiles of *Coelastrella* have been reported. An increase in the illuminance from 900 to 4200 lx significantly affected the content of palmitic, stearic, and linoleic acids in the biomass of *Coelastrella* sp. V3 grown on Bold’s basal medium^9^. Shifting BBM acidity to pH 5 led to increased content of oleic acid, while maintaining it at pH 7 led to increased content of α-linolenic acid in the strain *C. rubescens* IPPAS H-350^7^. Exclusion of nitrogen and phosphorus from BG11 medium favored accumulation of oleic and linoleic acids in *Coelastrella* sp., while combined mixotrophic/heterotrophic cultivation with depletion of nitrogen and phosphorus reduced the content of palmitic acid in favor of oleic acid^11^. These findings indicate that various types of stress (nitrogen and phosphorus starvation, altered pH and luminance, and the alternation of mixotrophic and heterotrophic nutrition) significantly affect the contributions of palmitic, oleic, linoleic, and α-linolenic acids to fatty acid profiles in most *Coelastrella* strains.

Figure 2 illustrates the existence of many more species and phylogenetic lineages of *Coelastrella* and *Coelastrum*, the biomass of which has yet to be analyzed for fatty acid content. Relation of the fatty acid profiles to phylogenetic positions of certain algal strains reveals, for example, significant predominance of omega-3 over omega-6 polyunsaturated fatty acids characteristic of the “core” *Coelastrella* clade species. Outside the “core” *Coelastrella* clade, the predominance of saturated over polyunsaturated fatty acids can be observed, for example, in *Coelastrella* sp. M60, *Coelastrella* sp. FI69, and *C. vacuolata* (I. Shihira et R.W.Krauss) Hegewald et N. Hanagata SAG 211-8b.

Both *C. multistriata* MZ–Ch23 and *C. striolata* CAUP H 3602 contain almost equal amounts of saturated and polyunsaturated fatty acids; the same is true for omega-3 and omega-6 polyunsaturated fatty acids. Such characteristics can be taken into account as chemotaxonomic markers. Another feature of *C. multistriata* and *C. striolata* (when grown under normal cultivation conditions) is the dominance of 7,10,13-hexadecatrienic (15.20–19.60%) and α-linolenic (38.10–44.20%) acids among omega-3 polyunsaturated fatty acids. Apart from that, some strains present with higher concentrations of minor fatty acid species, e.g. 4,7,11,13-hexadecatetraenoic (7.1%) and stearidonic (4.0%) acids in *C. vacuolata* and 11,14,17-eicosatrienoic acid (5.2%) in *Coelastrella* sp. SAG 2123^4^.

Prospective feedstocks for the balanced fatty acids formulations must have the fatty acids composition adjusted to physiological needs of the consumer^28,29^. The no less than 1:4 ratio between omega-3 and omega-6 polyunsaturated fatty acids in human diet is considered healthy and has been shown to reduce the dosage of pharmaceuticals required for an individual^30^. A screening for potential biomass sources should therefore be keen on sufficiently low omega-6 fatty acids concentrations accompanied by high omega-3 fatty acid concentrations. In *C. multistriata* MZ–Ch23, polyunsaturated fatty acids constituted 38.78–66.80% of total fatty acids (stress experiments included), which exceeded the proportion of saturated fatty acids (21.16–29.60%). Most analyzed strains of *Coelastrella* (Fig. 2) are characterized by high content of both omega-3 and omega-6 fatty acids; the ranges are 29.6–59.4% and 8.8–51.3%, respectively. Moreover, most of the strains reveal prevalence of omega-3 over omega-6 by the factor of 1.8–6.7. The highest omega-3 to omega-6 ratio (6.7:1) was recorded for strain *C. vacuolata* SAG 211-8b^4^ and the lowest value (1:1) was recorded for *C. rubescens* IPPAS H-350^7^. Among other studied genera phylogenetically close to *Coelastrella*, the highest omega-3 to omega-6 ratio of 9.4:1 was observed in *Coelastrum pseudomicroporum* Korshikov SAG 33.88 (Fig. 2,^4^). The opposite excess of omega-6 over omega-3 fatty acids has been reported *Coelastrella* sp. SAG 2123 (2.3:1^4^) and *Coelastrella* sp. FI69 (2.1:1^12^). In the studied strain *C. multistriata* MZ–Ch23, the ratio of biotechnologically valuable fatty acids is dominated by omega-3 species, especially in stress-free cultures (1.4:1 to 6.6:1 dependent on the conditions). The high content of omega-3 fatty acids, especially α-linolenic acid, highlights the novel strain MZ–Ch23 as a prospective source of biomass for the food and pharmaceutical industries, agriculture, and aquaculture. The high content of saturated and monounsaturated fatty acids in MZ–Ch23 biomass obtained under nitrogen-and-phosphorus starvation is promising as well (61.09% of total fatty acids, which is close to corresponding values for vegetable oils used as feedstock for biodiesel production^31^).

## Conclusions

The newly identified strain of green algae *Coelastrella multistriata* MZ–Ch23 reveals characteristic growth dynamics and lipid production in culture. This strain is the first known representative of *C. multistriata* in the wild algal flora of Russia. Lipid and fatty acid profiles of *C. multistriata* MZ–Ch23 are sensitive to nitrogen and phosphorus starvation, with the strongest response observed for palmitic, oleic, linoleic, and α-linolenic fatty acids. A 25-day cultivation protocol using fully supplemented Bold's basal medium afforded high yields of biomass rich in polyunsaturated and omega-3 fatty acids. Extension of the cultivation time to 60 days enhanced the biomass production, while preserving it sufficiently rich in lipids and fatty acids, primarily polyunsaturated. The 60-days-old cultures had the highest content and diversity of very long-chain fatty acids (9.99% of total fatty acids). Simultaneous depletion of nitrogen and phosphorus from the growth medium promoted accumulation of lipids and fatty acids in the biomass with maximal efficiency (respectively, 639.8 mg L^–1^ and 654.3 mg L^–1^). In this case, monounsaturated fatty acids become dominant in the spectrum, primarily due to oleic acid (33.7% of total fatty acids corresponding to 220.5 mg L^–1^). The developed cultivation strategies can be used to obtain *C. multistriata* biomass for multiple R&D purposes. High yields of saturated and monounsaturated fatty acids, obtained under depletion of nitrogen and phosphorus, position the novel MZ–Ch23 strain as a promising feedstock for biofuel production.

## Materials and methods

### Isolation

The novel strain MZ–Ch23 of green algae was isolated by micropipetting from a fouled glass culture using an inverted Zeiss Axio Vert A1 microscope (Zeiss, Germany). The culture originated from mineral waste (a mixture of loam with shale and quartzite) collected from young dump (up to 10 years old) of the Gurovo quarry (N 54°28′36.05″, E 37°19′24.68″, June 15, 2016, Tula region, Russia). A Zeiss Scope A1 microscope equipped with an oil immersion lens (× 100/n.a. 1.4, DIC) was used to conduct light microscopy and microphotography. The structure of mucus was revealed by staining cells with 0.1% methylene blue solution and 1.0% ink solution. Observations of the strain lasted from 7 days to 3 months. The cultures were maintained on BBM^32^. The strain was deposited in the Algae Collection of Molecular Systematics of Aquatic Plants at К.A. Timiryazev Institute of Plant Physiology RAS and the Collection of Algae at Bogdan Khmelnitsky Melitopol State Pedagogical University CAMU (WDCM1158) as perpetually-transferred pure cultures.

### Molecular analysis

Total DNA from the studied strain MZ–Ch23 was extracted using Chelex 100 Chelating Resin, molecular biology grade (Bio-Rad Laboratories, USA), according to the manufacturer’s protocol 2.2. A fragment of 18S rDNA gene (487 bp, including the highly variable V4 region) was amplified using D512for and D978rev primers by Zimmerman et al.^33^. Amplification of the 605 bp ITS1–5.8S rDNA–ITS2 region was performed using ITS1 and ITS4 primers by White et al.^34^.

Amplifications were carried out using premade polymerase chain reaction (PCR) mastermixes (ScreenMix by Evrogen, Russia). Amplification conditions for 18S rDNA gene were as follows: initial denaturation for 5 min at 95 ºC followed by 35 cycles of 30 s denaturation at 94 ºC, 30 s annealing at 52 ºC, and 50 s extension at 72 ºC, with the final extension for 10 min at 72 ºC. Amplification conditions for the ITS1–5.8S rDNA–ITS2 region were as follows: initial denaturation for 5 min at 95 °C followed by 35 cycles of 30 s denaturation at 94 °C, 30 s annealing at 60 °C, and 60 s extension at 72 °C, with the final extension for 5 min at 72 °C. PCR products were visualized by horizontal electrophoresis in 1.0% agarose gel stained with SYBRTM Safe (Life Technologies, USA). The products were purified with a mixture of FastAP, 10 × FastAP Buffer, Exonuclease I (Thermo Fisher Scientific, USA), and water. The sequencing was performed using a Genetic Analyzer 3500 instrument (Applied Biosystems, USA).

Editing and assembling of the consensus sequences were carried out by processing the direct and reverse chromatograms in Ridom TraceEdit (ver. 1.1.0) and Mega7 software^35^. The reads were included in the alignments along with corresponding sequences of 33 green algae species downloaded from GenBank (taxa names and Accession Numbers are given in Fig. 2). The outgroup comprised two strains of *Scenedesmus obtusus* Meyen (Scenedesmaceae).

The alignments for all strains including outgroups were obtained by means of comparing secondary structures of the 18S rDNA gene and the ITS1–5.8S rDNA–ITS2 region presented for the strain *Coccomyxa elongata* Chodat et Jaag MZ–Ch64^36^. The resulting alignments had lengths of 1118 characters (Supplementary material 1).

The data set was analyzed using the BI method implemented in Beast ver. 1.10.1 software^37^ to construct phylogeny. For the alignment partition the most appropriate substitution model, shape parameter α and a proportion of invariable sites (pinvar) were estimated using the Bayesian information criterion (BIC) as implemented in jModelTest 2.1.10^38^. This BIC-based model selection procedure selected the JC + I model and pinvar = 0.8050 for the 18S rDNA gene; TPM3 + G model and α = 0.2650 for the ITS1–5.8S rDNA–ITS2 region. We used the HKY model of nucleotide substitution instead of JC, the GTR model instead of TPM3 given that they were the best matching model available for BI. A Yule process tree prior was used as a speciation model. The analysis ran for 10 million generations with chain sampling every 1000 generations. The parameters-estimated convergence, effective sample size (ESS) and burn-in period were checked using the Tracer ver. 1.7.1 software^37^. The initial 25% of the trees were removed, the rest were retained to reconstruct a final phylogeny. The phylogenetic tree and posterior probabilities of its branching were obtained on the basis of the remaining trees, having stable estimates of the parameter models of nucleotide substitutions and likelihood. The ML analysis was performed using RAxML program^39^. The nonparametric bootstrap analysis with 1000 replicas was used. The phylogenetic tree topology is available online in the Supplementary material 2. The programs FigTree ver. 1.4.4 and Adobe Photoshop CC (19.0) were used for viewing and editing of the trees.

The Mfold version 2.5 software was used to model secondary structure of ITS2^40^. The presence of UU pyrimidine–pyrimidine unpaired section in the second hairpin and the conservative GGUAG motive in the 5′ side of helix III^41^, as well as the length and nucleotide composition of the spacers in the central loop determining the helix boundaries, were taken into consideration when constructing the final ITS2 model^42^. Construction of a hybrid stem with the 5.8S rDNA terminal site and the complementary LSU start site determined the beginning and the end of ITS2. The PseudoViewer3 program was used to visualize the resulting secondary structure^43^ available online in the Supplementary material 3.

### Cultivation

Four sets of cultivation conditions were used to assess fatty acid profiles of microalga at different nitrogen and phosphorus concentrations: (1) Bold’s basal medium with 2.94 mM NaNO_3_, 0.43 mM K_2_HPO_4_, and 1.29 mM KH_2_PO_4_ (control); (2) BBM depleted of NaNO_3_ (-N); (3) BBM depleted of K_2_HPO_4_ and KH_2_PO_4_ (-P); and (4) BBM depleted of NaNO_3_, K_2_HPO_4_, and KH_2_PO_4_ (-N-P). All experiments were conducted in three independent culture replicates.

The cultures were maintained in 250-mL Erlenmeyer glass flasks with 150 mL medium, under constant orbital shaking (150 rpm in ELMI Sky Line Shaker S-3L, ELMI Ltd, Latvia) for 25 or 60 days at 25 °C. The light intensity was 100 μmol photons m^−2^ s^−1^ with 16:8 h light/dark photoperiod. IMPLEN Nanophotometer P300 (Implen GmbH, Germany) was used to measure optical density at λ = 720 nm (OD_720_). Cell concentrations were measured with a TC20 Automated Cell Counter (Bio-Rad Laboratories, USA). The initial cultures had OD_720_ of 0.07925 corresponding to 0.01 × 10^6^ cells mL^−1^.

### Total lipid and triacylglyceride content measurements

The total lipid content was measured using Bligh and Dyer method^44^ with modifications^45^. The algal suspension was transferred to 50 mL tubes and centrifuged for 3 min at 3600*g*. The freeze-drying process was carried out in a Labconco FreeZone 2.5 L Benchtop Freeze Dryer (Labconco Corporation, USA) at a vacuum set point of 0.1 mBar. Freeze-dried biomass (200 mg DW) was mixed with 3.2 mL methanol and homogenized. After homogenization, 4 mL chloroform and 4 mL methanol were added and mixed for 15 min. After mixing, 4 mL chloroform and 4 mL 0.3% NaCl solution were added. The upper fraction containing methanol and NaCl solution was discarded. The lower fraction containing chloroform and lipids was evaporated, and lipid content was determined gravimetrically.

Triacylglyceride determination was carried out according to Mamaeva et al.^45^ and Maltsev et al.^46^. TAGs were extracted from 25 to 40 mg dry biomass with 650 μL of methanol-dichloromethane (1:1) using homogenization with glass beads. The extract was centrifuged at 1517 g and the supernatant was collected. The extraction was repeated three times; the three extracts were mixed and filtered through a 0.22-μm PTFE filter prior to analysis. High-performance liquid chromatography analysis of the lipid extract was performed in a gradient mode using an Agilent 1260 Infinity Series chromatograph (Agilent Technologies, USA) with a normal-phase column and Agilent G4260 evaporative light scattering detector (ELSD). The detector temperature was held at 50 °C and nitrogen was used as nebulizing gas at a flow rate of 10 L min^−1^. The mobile phase was a mixture of solvent A (a mixture of hexane 98% and methyl *tert*-butyl ether 2%) and solvent B (a mixture of hexane 39.2%, methyl *tert*-butyl ether 0.8%, isopropanol 52%, and water 8%) with the gradient composition descending from 100% A to 100% B in 15 min and to 100% A in 25 min. Injection volumes of 10 μL and sample flow rates of 1 mL min^−1^ were used in all experiments. The column temperature was held constant at 40 °C. The total analysis time was 25 min. The column and detector signal were calibrated using a TAG–tristearin 18:0 standard^47^. Individual peaks were identified by comparing their retention times with those of pure TAGs in standard mixtures. TAGs were quantified by peak area using calibration curve.

### Fatty acid composition analysis

Biomass preparation for the fatty acid methyl ester (FAME) profiling was performed according to Maltsev et al.^46^. The MZ–Ch23 algal suspensions were transferred to 15–50 mL tubes (depending on the volume). The cells were pelleted at room temperature for 3 min at 3600*g*. The supernatant was removed, and the pelleted cells were resuspended in 10–15 mL volume (depending on the amount of biomass) of distilled water, quantitatively transferred to 15 mL centrifuge tubes and pelleted again by centrifugation. The supernatant was removed and the entire quantity was transferred to a 50-mL round-bottom flask. Heptadecanoic acid (Sigma-Aldrich, USA) was used as internal standard for the fatty acid composition determination. To avoid the oxidation of unsaturated fatty acids, all samples were processed under argon atmosphere. Ten milliliters of 1 M KOH in 80% aqueous ethanol was added to the dry residue, the flask was sealed with a reflux condenser, and kept for 60 min at the boiling point of the mixture (~ 80 °C). After the time lapse, the solvents were evaporated in vacuo. The resulting volume of ~ 3 mL was quantitatively transferred to a 50-mL centrifuge tube with distilled water added to a total volume of 25 mL. Unsaponifiable components were extracted with three 10 mL changes of n-hexane (Himmed, Russia); the tube was centrifuged at room temperature for 5 min at 2022*g* to accelerate phase separation. After that, the aqueous phase was acidified with few drops of 20% sulfuric acid (Himmed, Russia) to a slightly acidic pH (checked with indicator paper) and free fatty acids were extracted with 20 mL of *n*-hexane. The hexane solution of free fatty acids was transferred to a dry 50-mL round-bottom flask and the solvent was evaporated to dryness on a rotary evaporator (IKA RV-10, Germany), after which 10 mL of absolute methanol (Sigma-Aldrich, USA) and 1 mL of acetyl chloride (Sigma-Aldrich, USA) were added to the dry residue. The flask, closed with a reflux condenser, was kept for one hour at 70 °C, then the solvents were evaporated to dryness, a few drops of distilled water were added to the dry residue, and FAMEs were extracted with *n*-hexane.

The obtained FAMEs were analyzed using an Agilent 7890A gas–liquid chromatograph (Agilent Technologies) equipped with an Agilent 5975C mass spectrometric detector. A DB-23 capillary column (B&W, USA), 60 m long and 0.25 mm in diameter, was used for the analysis. The remaining conditions of the analysis were as follows: carrier gas helium at a flow rate of 1 mL min^−1^, injected sample volume 1 μL, 1:5 flow split ratio, and evaporation temperature 260 °C. The temperature gradient program was as follows: 130 °C to 170 °C at 6.5 °C min^−1^ steps; 170 °C to 215 °C at 2.5 °C min^−1^ increments, hold at 215 °C for 25 min, 215 °C to 240 °C at 40 °C min^−1^ increments, and the final hold at 240 °C for 50 min; operating temperature of the mass spectrometric detector was 240 °C and ionization energy was 70 eV.

### Data analysis

All analyses were performed in triplicate. Figures show the mean values and standard errors. Significance of differences between the groups was evaluated by one-way analysis of variance (ANOVA) with a Tukey's post-hoc test performed using Statgraphics Centurion ver. 18 software. A difference between two groups was declared significant at *P* < 0.05. Analysis of variance tables are available online in the Supplementary material 4.

## Supplementary Information

Supplementary Information 1.

Supplementary Information 2.

Supplementary Information 3.

Supplementary Information 4.

Supplementary Information 5.
