# Supplementary material for: Lipid accumulation by Coelastrella multistriata (Scenedesmaceae, Sphaeropleales) during nitrogen and phosphorus starvation
Source: Sci Rep. 2021 Oct 6;11:19818. doi: 10.1038/s41598-021-99376-9 (PMC8494790; doi:10.1038/s41598-021-99376-9)
Supplement: Supplementary file 1 — Supplementary Information 1. [file 41598_2021_99376_MOESM1_ESM.docx]

SUPPLEMENTARY INFORMATION

**Lipid accumulation by *Coelastrella multistriata* (Scenedesmaceae, Sphaeropleales) during nitrogen and phosphorus starvation**

Yevhen Maltsev^1^*, Zinaida Krivova^1^, Svetlana Maltseva^1^, Kateryna Maltseva^2^, Elena Gorshkova^1^, Maxim Kulikovskiy^1^

^1^ К.А. Timiryazev Institute of Plant Physiology RAS, IPP RAS, Moscow, 127276, Russia

^2^ Bogdan Khmelnitsky Melitopol State Pedagogical University, Melitopol, 72312 Ukraine

**Supplementary material 1** Alignment of the 18S rDNA gene partial sequence and ITS1–5.8S rDNA–ITS2 region for different representatives of Sphaeropleales, in .doc format.

**Supplementary material 2** The Bayesian phylogenetic tree topology, in .doc format.

**Supplementary material 3** Predicted secondary structure of the ITS2 for *Coelastrella multistriata* MZ–Ch23, in .doc format.

**Supplementary material 4** Analysis of variance tables, in .pdf format.
