# Supplementary material for: Lipid accumulation by Coelastrella multistriata (Scenedesmaceae, Sphaeropleales) during nitrogen and phosphorus starvation
Source: Sci Rep. 2021 Oct 6;11:19818. doi: 10.1038/s41598-021-99376-9 (PMC8494790; doi:10.1038/s41598-021-99376-9)
Supplement: Supplementary file 2 — Supplementary Information 2. [file 41598_2021_99376_MOESM2_ESM.docx]

>Acutodesmus_obliquus_SAG_22.81

gccagcagccgcggtaattccagctccaatagcgtatatttaagttgttgcagttaaaaagctcgtagttggatttcgggtgggttctagcggtccgcctagtgtgagtactgctatggccttcctttctgtcggggacgggcttctgggcttcactgtccgggactcggagtcgacgtggttactttgagtaaattagagtgttcaaagcaggcttacg-ccagaatactttagcatggaataacacgataggactctggcctatcttgttggtctgtaggaccggagtaatgattaagagggacagtcgggggcattcgtatttcattgtcagaggtgaaattcttggatttatgaaagacgaactactgcgaaagcatttgccaaggatgttttcattaatcaagaacgaaagttgggggctcgaagacgattagataccgtcgtagtctcaaccataaacgatgccgactagggattggcgaaaattggctt----gcccaattgtacttgcaag-ctgg--tgcgagtaatttgattacttgc----atcagtgg-cgctttggcatgc-ttatacacc-agtgctaaccactgtcaaaaccaaactctgaagctttgattgcta-ttaactggcaatcttaaccaaagacaactctcaacaacggatatcttggctctcgcaacgatgaagaacgcagcgaaatgcgatacgtagtgtgaattgcagaattccgtgaaccatcgaatctttgaacgcatattgcgctcgagccctcgggcaagagcatgtctgcctcagcgtcggtttataccctcacccctctctccttttggagggctggtcagc--ttctagttggccttaggggtggatctggctttcccaattggtt----------cactccgattgggttggctgaagcttagaggc-ttaagcaaggacccgatatgggcttc-aactggataggtagcaccggcttctgccgactacacgaagttgtggcttgtggactttgctagaggccaagcaggaa-catg-ctttgcatgtctt-aaacnnnnnnnnnnnnnnnnnnnnnnnnnnnnnnnnnnnnnnnnnnnnnnnnn

>Chlorella_emersonii_CCAP_211_15

gccagcagccgcggtaattccagctccaatagcgtatatttaagttgttgcagttaaaaagctcgtagttggatttcgggtgggttctagcggtccgcctatggtgagtactgctatggccttcctttctgtcggggacgggcttctgggcttaattgtccgggactcggagtcgacgtggttactttgagtaaattagagtgttcaaagcaggcttacgccctgaatactttagcatggaataacacgataggactctggcctatcttgttggtctgtaggactggagtaatgattaagagggacagtcgggggcattcgtatttcattgtcagaggtgaaattcttggatttatgaaagacgaactactgcgaaagcatttgccaaggatgttttcattaatcaagaacgaaagttgggggctcgaagacgattagataccgtcgtagtctcaaccataaacgatgccgactagggattggcgaaatccgtcaac-tgcgttggccacc-ctcacag-ctca---gtgggcaat---gctgccgac-----tgagtgg-cgccttagcatgc-ttatacacc-agtgctaaccactgttgaaaccaaactctgaagttgtgattgcta-ttaattggcaatcttaaccaaagacaactctcaacaacggatatcttggctctcgcaacgatgaagaacgcagcgaaatgcgatacgtagtgtgaattgcagaattccgtgaaccatcgaatctttgaacgcatattgcgctcgagccctcgggcaagagcatgtctgcctcagcgtcggtttacaccctcacccctccctcttttt-gtgggtcagttggc--atattgccagcc-taggggtggatctggcttccccaatctgccgc--------ttggtgggttgggttggctgaagtacagaggc-ttaagcaaggacccgatatgggcttcaaactggataggtagctccggcttgtgccgactacacgaagttgtggcttgtggatcttgctaggagcccagcaggaaccgtg-ccttaggcatgtataaacattcgacctgagctcaggcaaggctacccgctgaacttaagcatat-ca

>Chlorella_fusca_var._vacuolata_UTEX252

gccagcagccgcggtaattccagctccaatagcgtatatttaagttgttgcagttaaaaagctcgtagttggatttcgggtgggttctagcggtccgcctatggtgagtactgctatggnnnnnnnnnnnnnnnnnnnnnnnnnnnnnnnnnnnnnnnnnnnnnnnnnnnnnnnnnnnnnnnnnnnnnnnnnnnnnnnnnnnnnnnnnnnnnnnnnnnnnnnnnnnnnnnnnnnnnnnnnnnnnnnnnnnnnnnnnnnnnnnnnnnnnnnnnnnnnnnnnnnnnnnnnnnnnnnnnnnnnnnnnnnnnnnnnnnnnnnnnnnnnnnnnnnnnnnnnnnnnnnnnnnnnnnnnnnnnnnnnnnnnnnnnnnnnnnnnnnnnnnnnnnnnnnnnnnnnnnnnnnnnnnnnnnnnnnnnnnnnnnnnnnnnnnnnnnnnnnnnnnnnnnnnnnnnnnnnnnnnnnnnnnnnnnnnnnnnnnnnnnnnnnnatccgtcaac-tgcgttggccacc-ctcacag-ctca---gtgggcaat---gctgccgac-----tgagtgg-cgccttagcatgc-ttatacacc-agtgctaaccactgttgaaaccaaactctgaagttgtgattgcta-ttaattggcaatcttaaccaaagacaactctcaacaacggatatcttggctctcgcaacgatgaagaacgcagcgaaatgcgatacgtagtgtgaattgcagaattccgtgaaccatcgaatctttgaacgcatattgcgctcgagccctcgggcaagagcatgtctgcctcagcgtcggtttacaccctcacccctccctcttttt-gtgggtcagttggc--atattgccagcc-taggggtggatctggcttccccaatctgccgc--------ttggtgggttgggttggctgaagtacagaggc-ttaagcaaggacccgatatgggcttc-aactggataggtagctccggcttgtgccgactacacgaagttgtggcttgtggatcttgctaggagcccagcaggaa-cgtg-ccttaggcatgtataaacattcgacctgagctcaggcaaggctacccgctgaacttaagcatat-ca

>Coelastrella_aeroterrestrica_SWK1_2

gccagcagccgcggtaattccagctccaatagcgtatatttaagttgttgcagttaaaaagctcgtagttggatttcgggtgggttctagcggtccgcctatggtgagtactgctatggccttcctttctgtcggggacgggcttctgggcttcactgtccgggactcggagtcgacgtggttactttgagtaaattagagtgttcaaagcaggcttacgccctgaatactttagcatggaataacacgataggactctggcctatcttgttggtctgtaggactggagtaatgattaagagggacagtcgggggcattcgtatttcattgtcagaggtgaaattcttggatttatgaaagacgaactactgcgaaagcatttgccaaggatgttttcattaatcaagaacgaaagttgggggctcgaagacgattagataccgtcgtagtctcaaccataaacgatgccgactagggattggcgaaattcggcata-caacccgtttgta-ctcacag-ctgg---gtaggcact---gcagcctgc-----tcagtgg-cgccttggtatga-ctttacacc-agtgctaaccactgat-aaaccaaactctgaagtaatgattgcta-gcaactggcaattataaccaaagacaactctcaacaacggatatcttggctctcgcaacgatgaagaacgcagcgaaatgcgatacgtagtgtgaattgcagaattccgtgaaccatcgaatctttgaacgcatattgcgctcgagccctcgggcaagagcatgtctgcctcagcgtcggtttacaccctcacccctcc---cttac--tgggtgtgtttgctctgtttgcaagccattggggtggatctggcttccccaatcagccgt--ttcactgcggcaggttgggttggctgaagtgcagaggc-ttaagcaaggacccgatatgggcttc-aactggataggtagcaccggcttctgccgactacacgaagttgttgcttgtggatcttgctaggagccaagcaggaa-cgtg-cctttggcatgtctaaactttcgacctgagctcaggcaagattacccgctgaacttaagcatat-ca

>Coelastrella_corcontica_CCALA_308

gccagcagccgcggtaattccagctccaatagcgtatatttaagttgttgcagttaaaaagctcgtagttggatttcgggtgggttctagcggtccgcctatggtgagtactgctatggccttcctttctgtcggggacgggcttctgggcttcactgtccgggactcggagtcgacgtggttactttgagtaaattagagtgttcaaagcaggcttacgccctgaatactttagcatggaataacacgataggactctggcctatcttgttggtctgtaggactggagtaatgattaagagggacagtcgggggcattcgtatttcattgtcagaggtgaaattcttggatttatgaaagacgaactactgcgaaagcatttgccaaggatgttttcattaatcaagaacgaaagttgggggctcgaagacgattagataccgtcgtagtctcaaccataaacgatgccgactagggattggcgaaattcggcata-aaacccgtttgta-ctcacag-ctga---gtagacgtt---ggtgcctac-----tcagtgg-cgccttggcatgt-ctatacacc-aatgctaaccactgtt-aaaccaaactctgaagcattgattgcta-gcaactggcaatcttaaccaaagacaactctcaacaacggatatcttggctctcgcaacgatgaagaacgcagcgaaatgcgatacgtagtgtgaattgcagaattccgtgaaccatcgaatctttgaacgcatattgcgctcgagccctcgggcaagagcatgtctgcctcagcgtcggtttacaccctcacccctcccatcttgt--tgggcgtgtttgctctgtttgcaagccattggggtggatctggcttccccaatcatgtccgtttcactgcggcagattgggttggctgaagtgcagaggc-ttaagcaaggacccgatatgggcttc-aactggataggtagcaccggcttttgccgactacacgaagttgttgcttgtggatcttgctaggagccaagcaggaa-cgtg-cctctggcatgtcnnnnnnnnnnnnnnnnnnnnnnnnnnnnnnnnnnnnnnnnnnnnnnnnnnnnnn

>Coelastrella_oocystiformis_FACHB-2312

gccagcagccgcggtaattccagctccaatagcgtatatttaagttgttgcagttaaaaagctcgtagttggatttcgggtgggttctagcggtccgcctatggtgagtactgctatagccttcctttctgtcggggacgggcttctgggcttcactgtccgggactcggagtcgacgtggttactttgagtaaattagagtgttcaaagcaggcttacgccctgaatactttagcatggaataacacgataggactctggcctatcttgttggtctgtaggactggagtaatgattaagagggacagtcgggggcattcgtatttcattgtcagaggtgaaattcttggatttatgaaagacgaactactgcgaaagcatttgccaaggatgttttcattaatcaagaacgaaagttgggggctcgaagacgattagataccgtcgtagtctcaaccataaacgatgccgactagggattggcgaaattcggcata-aaacccgtttgta-ctcacag-ccgg---gtgggcatt---gctgcctgc-----tcggtgg-cgccttggcatga-ctgtacacc-agtgctaaccattgtcaaaaccaaattctgaagtaacgattgcta-gtaactggcaatcttaaccaaagacaactctcaacaacggatatcttggctctcgcaacgatgaagaacgcagcgaaatgcgatacgtagtgtgaattgcagaattccgtgaaccatcgaatctttgaatgcatattgcgctcgagccctcgggcaagagcatgtctgcctcagcgtcggtttacaccctcacccctcccaacttgt--tgggtgtgcttgctgcttttgcaagccactggggtggatctggcttccccaatccaccat--ta----taggtagattgggttggctgaagtgcagaggt-gtaagcaaggacccaatatgggcttc-aactggataggtagccccggcttttgccgactacacgaagttgttgcttgtggatcttgctagaagccaagcaggaa-cgtg-cctccggcacgtctaaactttcgacctgagctcaggcaagattacccgctgaacttaagcatat-ca

>Coelastrella_oocystiformis_SAG_277-1

nnnnnnnnnnnnnnnnnnnnnnnnnnnnnnnnnnnnnnnnnnnnnnnnnnnngttaaaaagctcgtagttggatttcgggtgggttctagcggtccgcctatggtgagtactgctatagccttcctttctgtcggggacgggcttctgggcttcactgtccgggactcggagtcgacgtggttactttgagtaaattagagtgttcaaagcaggcttacgccctgaatactttagcatggaataacacgataggactctggcctatcttgttggtctgtaggactggagtaatgattaagagggacagtcgggggcattcgtatttcattgtcagaggtgaaattcttggatttatgaaagacgaactactgcgaaagcatttgccaaggatgttttcattaatcaagaacgaaagttgggggctcgaagacgattagataccgtcgtagtctcaaccataaacgatgccgactagggattggcgaaattcggcata-agacccgtttgta-ctcacag-ccgg---gtgggcatt---gctgcctgc-----tcggtgg-cgccttggcatga-ctgtacacc-agtgctaaccactgtcaaaaccaaattctgaagtaacgattgcta-gtaactggcaatcttaaccaaagacaactctcaacaacggatatcttggctctcgcaacgatgaagaacgcagcgaaatgcgatacgtagtgtgaattgcagaattccgtgaaccatcgaatctttgaacgcatattgcgctcgagccctcgggcaagagcatgtctgcctcagcgtcggtttacaccctcacccctcccaacttgt--tgggtgtgcttgctgtttttgcaagccactggggtggatctggcttccccaatcagccat--ta----taggcagattgggttggctgaagtgcagaggc-gtaagcaaggacccaatatgggcttc-aactggataggtagccccggcttttgccgactacacgaagttgttgcttgtggatcttgctagaagccaagcaggaa-cgtg-cctccggcacgtctaaactttcgacctgagctcaggcaagattacccgctgaacttaagcatat-ca

>Coelastrella_rubescens_FACHB-2295

gccagcagccgcggtaattccagctccaatagcgtatatttaagttgttgcagttaaaaagctcgtagttggatttcgggtgggttctagcggtccgcctatggtgagtactgctatggccttcctttctgtcggggacgggcttctgggcttaactgtccgggactcggagtcgacgtggttactttgagtaaattagagtgttcaaagcaggcttacgccctgaatactttagcatggaataacacgataggactctggcctatcttgttggtctgtaggactggagtaatgattaagagggacagtcgggggcattcgtatttcattgtcagaggtgaaattcttggatttatgaaagacgaactactgcgaaagcatttgccaaggatgttttcattaatcaagaacgaaagttgggggctcgaagacgattagataccgtcgtagtctcaaccataaacgatgccgactagggattggcgaaattcggc-ca-aaacccgtttgta-ctcacag-ctga---gtgggcatt---gcgacctgc-----tcagtgg-cgccttggcatga-ctttacacc-agtgctaaccactgttaaaaccaaactctgaagtattggttgctg-gtaactggcaatcttaaccaaagacaactctcaacaacggatatcttgg-tctcgcaacgatgaagaacgcagcgaaatgcgatacgtagtgtgaattgcagaattccgtgaaccatcgaatctttgaacgcatattgcgctcaagccctcgggcaagagcatgtctgcctcagcgtcggtttacaccctcacccctcccaacttgt--tgggtgtgcttgctttattggcaagccgttggggtggatctggcttccccaatcagccat--------caggcagattgggttggctgaagtgcagaggc-ttaagcaaggacccgatatgggcttc-aactggataggtagcaccggcttctgccgactacacgaagttgttgcttgtggctcttgctagaggccaagcaggaa-cgtg-cctttggcatgtctaaactttcgacctgagctcaggcaagattacccgctgaacttaagcatat-ca

>Coelastrella_rubescens_IPPAS_H-350

gccagcagccgcggtaattccagctccaatagcgtatattt-agttgttgcagttaaaaagctcgtagttggatttcgggtgggttctagcggtccgcctatggtgagtactgctatggccttcctttctgtcggggacgggcttctgggcttaactgtccgggactcggagtcgacgtggttactttgagtaaattagagtgttcaaagcaggcttacgccctgaatactttagcatggaataacacgataggactctggcctatcttgttggtctgtaggactggagtaatgattaagagggacagtcgggggcattcgtatttcattgtcagaggtgaaattcttggatttatgaaagacgaactactgcgaaagcatttgccaaggatgttttcattaatcaagaacgaaagttgggggctcgaagacgattagataccgtcgtagtctcaaccataaacgatgccgactagggattggcgaannnnnnnnnnnnnnnnnnnnnnnnnnnnnnnnnnnnnnnnnnnnnnnnnnnnnnnnnnnnnnnnnnnnnnnnnnnnnnnnnnnnnnnnnnnnnnnnnnnnnnnnnnnnnnnnnnnnnnnnnnnnnnnnnnnnnnnnnnnnnnnnnnnnnnnnnnnnnnnaaccaaagacaactctcaacaacggatatcttggctctcgcaacgatgaagaacgcagcgaaatgcgatacgtagtgtgaattgcagaattccgtgaaccatcgaatctttgaacgcatattgcgctcnnnnnnnnnnnnnnnnnnnnnnnnnnnnnnnnnnnnnnnnnnnnnnnnnnnnnnnnnnnnnnnnnnnnnnnnnnnnnnnnnnnnnnnnnnnnnnnnnnnnnnnnnnnnnnnnnnnnnnnnnnnnnnnnnnnnnnnnnnnnnnnnnnnnnnnnnnnnnnnnnnnnnnnnnnnnnnnnnnnnnnnnnnnnnnnnnnnnnnnnnnnnnnnnnnnnnnnnnnnnnnnnnnnnnnnnnnnnnnnnnnnnnnnnnnnnnnnnnnnnnnnnnnnnnnnnnnnnnnnnnnnnnnnnnnnnnnnnnnnnnnnnnnnnnnnnnnnnnnnnnnnnnnnnnnnnnnnnnnnnnnnn

>Coelastrella_saipanensis_LY31-2

gccagcagccgcggtaattccagctccaatagcgtatatttaagttgttgcagttaaaaagctcgtagttggatttcgggtgggttctagcggtccgcctatggtgagtactgctatggccttcctttctgtcggggacgggcttctgggcttaactgtccgggactcggagtcgacgtggttactttgagtaaattagagtgttcaaagcaggcttacgccctgaatactttagcatggaataacacgataggactctggcctatcttgttggtctgtaggactggagtaatgattaagagggacagtcgggggcattcgtatttcattgtcagaggtgaaattcttggatttatgaaagacgaactactgcgaaagcatttgccaaggatgttttcattaatcaagaacgaaagttgggggctcgaagacgattagataccgtcgtagtctcaaccataaacgatgccgactagggattggcgaaattcgggttataagcccgtctgcatctcacag-ctgg---ttgggaaat---gctacccaa-----ccagtggccgccttggcatga-ccatacaccaagggccaaccgctgattaaaccaaactctgaagctgtgactgctatttgattggcagtcttaaccaaagacaactctcaacaacggatatcttggctctcgcaacgatgaagaacgcagcgaaatgcgatacgtagtgtgaattgcagaattccgtgaaccatcgaatctttgaacgcatattgcgctcgagccttcgggcgagagcatgtctgcctcagcgtcggtttacaccatcaccccccaaatttctttttgggtgtgtcggcatttattgctggctgtaggggtggatctgacttccccaacctgcaat--------gcgtttggttgggctagttgaagtgcagaggc-ttaagcaaggacccattaagggcttc-aactggataggtagcaccggcttgtgccgactacacgaagttgttgcttgtggatcttgct-ggtgccaagcaggaa-cgtgccaaatggcatgtctaaactttcgacctgagctcaggcaaggctacccgctgaacttaagcatattca

>Coelastrella_sp._FI69

gccagcagccgcggtaattccagctccaatagcgtatatttaagttgttgcagttaaaaagctcgtagttggatttcgggtgggttctagcggtccgcctatggtgagtactgctatggccttcctttctgtcggggacgggcttctgggcttaactgtccgggactcggagtcgacgtggttactttgagtaaattagagtgttcaaagcaggcttacgccctgaatactttagcatggaataacacgataggactctggcctatcttgttggtctgtaggactggagtaatgattaagagggacagtcgggggnnnnnnnnnnnntgtctgaggggtgaaattcttggatttatgaaagacgaactactgcgaaagcatttgccaaggatgttttcattaatcaagaacgaaagttgggggctcgaagacgattagataccgtcgtagtctcaaccataaacgatgccgactagggattggcgaannnnnnnnnnnnnnnnnnnnnnnnnnnnnnnnnnnnnnnnnnnnnnnnnnnnnnnnnnnnnnnnnnnnnnnnnnnnnnnnnnnnnnnnnnnnnnnnnnnnnnnnnnnnnnnnnnaaaaccaaannnnnnnnnnnnnnnnnnnnnnnnnnnnnnnnnnnnaaccaaagacaactctcaacaacggatatcttggctctcgcaacgatgaagaacgcagcgaaatgcgatacgtagtgtgaattgcagaattccgtgaaccatcgaatctttgaacgcatattgcgctcnnnnnnncgggcaagagcatgtctgcctcagcgtcggtttacaccctcacccctcccaacttgtnnnnnnnnnnnnnnnnnnnnnnnnnnnnnnnnnnnnnnnnnnnnnnnnnnnnnnnnnnnnnnnnnnnnnnnnnnnnnnnnnnnnnnnnnnnnnnnnnnnnnnnttaagcaaggacccgatatgggcttc-aactggataggtagcaccggcttctgccgactacacgaagttgttgcttgtggatcttgctaggagccaagcaggaannnnnnnnnnnnnnatgtctaaactttcgacctgagctcaggcaaggctacccgctgaacttaagcatat-ca

>Coelastrella_sp._M-60

gccagcagccgcggtaattccagctccaatagcgtatatttaagttgttgcagttaaaaagctcgtagttggatttcgggtgggttctagcggtccgcctatggtgagtactgctatggccttcctttctgtcggggacgggcttctgggcttaactgtccgggactcggagtcgacgtggttactttgagtaaattagagtgttcaaagcaggcttacgccctgaatactttagcatggaataacacgataggactctggcctatcttgttggtctgtaggactggagtaatgattaagagggacagtcgggggcgctttctttttggtgtcagaggtgaaattcttggatttatgaaagacgaactactgcgaaagcatttgccaaggatgttttcattaatcaagaacgaaagttgggggctcgaagacgattagaccccgtcgtagtctcaaccataaacgatgccgactagggattggcgaannnnnnnnnnnnnnnnnnnnnnnnnnnnnnnnnnnnnnnnnnnnnnnnnnnnnnnnnnnnnnnnnnnnnnnnnnnnnnnnnnnnnnnnnnnnnnnnnnnnnnnnnnnnnnnnnnaaaaccaaannnnnnnnnnnnnnnnnnnnnnnnnnnnnnnnnnnnaaccaaagacaactctcaacaacggatatcttggctctcgcaacgatgaagaacgcagcgaaatgcgatacgtagtgtgaattgcagaattccgtgaaccatcgaatctttgaacgcatattgcgctcnnnnnnncgggcaagagcatgtctgcctcagcgtcggtttacaccctcacccctcccaacttgtnnnnnnnnnnnnnnnnnnnnnnnnnnnnnnnnnnnnnnnnnnnnnnnnnnnnnnnnnnnnnnnnnnnnnnnnnnnnnnnnnnnnnnnnnnnnnnnnnnnnnnnttaagcaaggacccgatatgggcttc-aactggataggtagcaccggcttctgccgactacacgaagttgttgcttgtggatcttgctaggggccaagcaggaannnnnnnnnnnnnnatgtctaaactttcgacctgagctcaggcaaggctacccgctgaacttaagcatat-ca

>Coelastrella_sp._QW-2019b_FACHB-2314

gccagcagccgcggtaattccagctccaatagcgtatatttaagttgttgcagttaaaaagctcgtagttggatttcgggtgggttctagcggtccgcctatggtgagtactgctatggccttcctttctgtcggggacgggcttctgggcttcactgtccgggactcggagtcgacgtggttactttgagtaaattagagtgttcaaagcaggcttacgccctgaatactttagcatggaataacacgataggactctggcctatcttgttggtctgtaggactggagtaatgattaagagggacagtcgggggcattcgtatttcattgtcagaggtgaaattcttggatttatgaaagacgaactactgcgaaagcatttgccaaggatgttttcattaatcaagaacgaaagttgggggctcgaagacgattagataccgtcgtagtctcaaccataaacgatgccgactagggattggcgaaattcggt----aaatccgtatggagcccacag-ctga---gcgagcaat---gctgcccgc-----tcagtgg-cgccttggcatgc-ttatacacc-agtgctaaccactgttaaaaccaaatactgaatttgtgattgcta-gtaattggcaatcttaaccaaagataactctcaacaacggatatcttggctctcgcaacgatgaagaacgcagcgaaatgcgatacgtagtgtgaattgcagaattccgtgaaccatcgaatctttgaacgcatattgcgctcgagcctccgggcaagagcatgtctgcctcagcgtcggtttacaccctcacccctcccactttgt--tgggtt-gtttggcaactttgccagcataggtggtggatctggcttccccaatctgccgt--------ctggtagattgggttggctgaagaatagaggt-ttaagcaaggacccgttatgggcttc-aactggataggtagcaccggcttgtgccgactacacgaagttgatgctcgtggatcttgctagaggccaagcaggaa-cgtg-cccatggcacgtctaaactttcgacctgagctcaggcaaggctacccgctgaacttaagcatat-ca

>Coelastrella_sp._QW-2019c_FACHB-2300

gccagcagccgcggtaattccagctccaatagcgtatatttaagttgttgcagttaaaaagctcgtagttggatttcgggtgggttctagcggtccgcctatggtgagtactgctatggcctatctttctgtcggggacgggcttctgggcttaactgtccgggactcggagtcgacgtggttactttgagtaaattagagtgttcaaagcaggcttacgccctgaatactttagcatggaataacacgataggactctggcctatcttgttggtctgtaggactggagtaatgattaagagggacagtcgggggcattcgtatttcattgtcagaggtgaaattcttggatttatgaaagacgaactactgcgaaagcatttgccaaggatgttttcattaatcaagaacgaaagttgggggctcgaagacgattagataccgtcgtagtctcaaccataaacgatgccgactagggattggcgaagttcggctta-aaacccgctcgcatctcacag-ctgg---gtgggcatt---gctgcctgc-----tcagtgg-cgccttggcatga-ctatacacc-agtgctaaccactgttaaaactaaactctgaagttgtgattgcta-gtaactggcaatcttaaccaaagacaactctcaacaacggatatcttggctctcgcaacgatgaagaacgcagcgaaatgcgatacgtagtgtgaattgcagaattccgtgaaccatcgaatctttgaacgcatattgcgctcgagccttcgggcaagagcatgtctgcctcagcgtcggtttacaccctcacccctcccaacttgt--tgggtgtgtcggcttctctagctggccattggggtggatctggcttccccaatctgcttt--------actgcggattgggttggctgaagtgcagaggc-ttaagcaaggacccgttatgggcttc-aactggataggtagcaccggcttctgccgactacacgaagttgttgcttgtggatcttgctaggagccaagcaggaa-cata-cctatggtatgtctaaactttcgacctgagctcaggcaaggctacccgctgaacttaagcatat-ca

>Coelastrella_sp._QW-2019e_FACHB-2311

gccagcagccgcggtaattccagctccaatagcgtatatttaagttgttgcagttaaaaagctcgtagttggatttcgggtgggttctagcggtccgcctatggtgagtactgctatggccttcctttctgtcggggacgggcttctgggcttaactgtccgggactcggagtcgacgtggttactttgagtaaattagagtgttcaaagcaggcttacgccctgaatactttagcatggaataacacgataggactctggcctatcttgttggtctgtaggactggagtaatgattaagagggacagtcgggggcattcgtatttcattgtcagaggtgaaattcttggatttatgaaagacgaactactgcgaaagcatttgccaaggatgttttcattaatcaagaacgaaagttgggggctcgaagacgattagataccgtcgtagtctcaaccataaacgatgccgactagggattggcgaaattcggctta-aaacccgtctgta-ctcacag-ctgg---gtgggcatt---gctgcctgc-----tcagtgg-cgccttggcatgacctatacacc-agtgctaaccactgtcaaaaccaaactctgaagttgtgattgcta-gtaactggcaatcttaaccaaagacaactctcaacaacggatatcttggctctcgcaacgatgaagaacgcagcgaaatgcgatacgtagtgtgaattgcagaattccgtgaaccatcgaatctttgaacgcatattgcgctcgagccctcgggcaagagcatgtctgcctcagcgtcggtttacaccctcacccctcccaacttgt--tgggtgtgtttgcttctatagcaggccattggggtggatctggcttccccaatctgccat--------taggcgggttgggttggctgaagtgcagaggc-ttaagcaaggacccgatatgggcttc-aactggataggtagcaccggcttctgccgactacacgaagttgttgcttgtggatcttgctaggggccaagcaggaa-cgtgtcttttggcatgtctaaactttcgacctgagctcaggcaaggctacccgctgaacttaagcatat-ca

>Coelastrella_sp._SAG_2123

gccagcagccgcggtaattccagctccaatagcgtatatttaagttgttgcagttaaaaagctcgtagttggatttcgggtgggttctagcggtccgcctatggtgagtactgctatggcctatctttctgtcggggacgggcttctgggcttcactgtccgggactcggagtcgacgtggttactttgagtaaattagagtgttcaaagcaggcttacgccctgaatactttagcatggaataacacgataggactctggcctatcttgttggtctgtaggactggagtaatgattaagagggacagtcgggggcattcgtatttcattgtcagaggtgaaattcttggatttatgaaagacgaactactgcgaaagcatttgccaaggatgttttcattaatcaagaacgaaagttgggggctcgaagacgattagataccgtcgtagtctcaaccataaacgatgccgactagggattggcgaaattcggcgta-aaacccgtttgtatctcacag-ctgg---gtaggcaat---gttgcctat-----tcagtgg-cgccttggcatga-ctatacacc-agtgctaa-cactgttaaaaccaaactctgaagttgtgattgcta-gtaactggcaatcttaaccaaagacaactctcaacaacggatatcttggctctcgcaacgatgaagaacgcagcgaaatgcgatacgtagtgtgaattgcagaattccgtgaaccatcgaatctttgaacgcatattgcgctcgagccttcgggcaagagcatgtctgcctcagcgtcggtttaaaccctcacccctcccaacttgt-ttgggtgtgtcggcttctatagctggccacaggggtggatctggcttccccaatctgccat--------tcggtggattgggttggctgaagtgcagaggc-ttaagcaaggacccgatatgggcttc-aactggataggtagcaccggcttctgccgactacacgaagttgttgcttgtggatcttgctaggggccaagcaggaa-catg-cctatggtatgtctaaactttcgacctgagctcaggcaaggctacccgctgaacttaagcatat-ca

>Coelastrella_striolata_CAUP_H_3602

gccagcagccgcggtaattccagctccaatagcgtatatttaagttgttgcagttaaaaagctcgtagttggatttcgggtgggttctagcggtccgcctatggtgagtactgctatggccttcctttctgtcggggacgggcttctgggcttcactgtccgggactcggagtcgacgtggttactttgagtaaattagagtgttcaaagcaggcttacgccctgaatactttagcatggaataacacgataggactctggcctatcttgttggtctgtaggactggagtaatgattaagagggacagtcgggggcattcgtatttcattgtcagaggtgaaattcttggatttatgaaagacgaactactgcgaaagcatttgccaaggatgttttcattaatcaagaacgaaagttgggggctcgaagacgattagataccgtcgtagtctcaaccataaacgatgccgactagggattggcgaaattcggcata-aaacccgtttgta-ctcacag-ctga---gtagacgtt---gatgcctac-----tcagtgg-cgacttggcatgt-ctatacacc-agtgtcaaccactgtt-gaaccaaactctggagctttgattgcta-gcaactggcaatcttaaccaaagacaactctcaacaacggatatcttggctctcgcaacgatgaagaacgcagcgaaatgcgatacgtagtgtgaattgcagaattccgtgaaccatcgaatctttgaacgcatattgcgctcgagccctcgggcaagagcatgtctgcctcagcgtcggtttacaccctcacccctcccaacttgt--tgggtgtgcttgctctgtttgcaagccattggggtggatctggcttccccaatcatgtccgtttcacggcggcggattgggttggctgaagtgcagaggc-ttaagcaaggacccgatatgggcttc-aactggataggtagcaccggcttctgccgactacacgaagttgttgcttgtggatcttgctaggagccaagcaggaa-cgtg-cctttggcatgtctaaactttcgacctgagctcaggcaagactacccgctgaacttaagcatat-ca

>Coelastrella_striolata_var._multistriata_CCALA_309

gccagcagccgcggtaattccagctccaatagcgtatatttaagttgttgcagttaaaaagctcgtagttggatttcgggtgggttctagcggtccgcctatggtgagtactgctatggccttcctttctgtcggggacgggcttctgggcttcactgtccgggactcggagtcgacgtggttactttgagtaaattagagtgttcaaagcaggcttacgccctgaatactttagcatggaataacacgataggactctggcctatcttgttggtctgtaggactggagtaatgattaagagggacagtcgggggcattcgtatttcattgtcagaggtgaaattcttggatttatgaaagacgaactactgcgaaagcatttgccaaggatgttttcattaatcaagaacgaaagttgggggctcgaagacgattagataccgtcgtagtctcaaccataaacgatgccgactagggattggcgaaattcggcata-aaacccgtttgtg-ctcacag-ctga---gtggacgtt---gatgtctac-----tcagtgg-cgccttggcatgt-ctatacacc-aatgctaaccattgtt-aaaccaaactctgaagcattgattgcta-gcaactggcaatcttaaccaaagacaactctcaacaacggatatcttggctctcgcaacgatgaagaacgcagcgaaatgcgatacgtagtgtgaattgcagaattccgtgaaccatcgaatctttgaacgcatattgcgctcgagccctcgggcaagagcatgtctgcctcagcgtcggtttacaccctcacccctcccaacttgt--tgggcgtgtttgctctgtttgcaagccattggggtggatctggcttccccaatcatgtccgtttcactgcggcagactgggctggctgaagtgcagaggc-ttaagcaaggacccgatatgggcttc-aactggataggtagcaccggctcttgccgactacacgaagttgttgcttgtggatcttgctaggagccaagcaggaa-cgtg-cctttggcatgtctaaactttcgacctgagctcaggcaagactacccgctgaacttaagcatat-ca

>Coelastrella_terrestris_CAUP_H_4403

gccagcagccgcggtaattccagctccaatagcgtatatttaagttgttgcagttaaaaagctcgtagttggatttcgggtgggttctagcggtccgcctatggtgagtactgctatggnnnnnnnnnnnnnnnnnnnnnnnnnnnnnnnnnnnnnnnnnnnnnnnnnnnnnnnnnnnnnnnnnnnnnnnnnnnnnnnnnnnnnnnnnnnnnnnnnnnnnnnnnnnnnnnnnnnnnnnnnnnnnnnnnnnnnnnnnnnnnnnnnnnnnnnnnnnnnnnnnnnnnnnnnnnnnnnnnnnnnnnnnnnnnnnnnnnnnnnnnnnnnnnnnnnnnnnnnnnnnnnnnnnnnnnnnnnnnnnnnnnnnnnnnnnnnnnnnnnnnnnnnnnnnnnnnnnnnnnnnnnnnnnnnnnnnnnnnnnnnnnnnnnnnnnnnnnnnnnnnnnnnnnnnnnnnnnnnnnnnnnnnnnnnnnnnnnnnnnnnnnnnnnnattcggcata-aaacccgtttgta-ctcacag-ctggcacgtaggcatt---gctgcct-c-----tcagtgg-cgccttggcatga-ctttacacc-agtgctaaccactgttaaaaccaaaatctgaagtattgattgcta-gtaattggcgatcttaaccaaagacaactctcaacaacggatatcttggctctcgcaacgatgaagaacgcagcgaaatgcgatacgtagtgtgaattgcagaattccgtgaaccatcgaatctttgaacgcatattgcgctcgagccctcgggcaagagcatgtctgcctcagcgtcggtttacaccctcacccctcccaacttgt--tgggtatgtttgctttttttgcaagccacaggggtggatctggcttccccaatcagccat--------caggtagattgggttggctgaagtacagaggc-ttaagcaaggacccgatatgggcttc-aactggataggtagcaccggcttttgccgactacacgaagttgttgcttgtggatcttgctagaagccaagcaggaa-tgtg-cctttggcatatcttaactttcgacctgagctcaggcaagattacccgctgaacttaagcatat-ca

>Coelastrella_terrestris_CCALA_476

gccagcagccgcggtaattccagctccaatagcgtatatttaagttgttgcagttaaaaagctcgtagttggatttcgggtgggttctagcggtccgcctatggtgagtactgctatggccttcctttctgtcggggacgggcttctgggcttcactgtccgggactcggagtcgacgtggttactttgagtaaattagagtgttcaaagcaggcttacgccctgaatactttagcatggaataacacgataggactctggcctatcttgttggtctgtaggactggagtaatgattaagagggacagtcgggggcattcgtatttcattgtcagaggtgaaattcttggatttatgaaagacgaactactgcgaaagcatttgccaaggatgttttcattaatcaagaacgaaagttgggggctcgaagacgattagataccgtcgtagtctcaaccataaacgatgccgactagggattggcgaaattcggcata-aaacccgtttgta-ctcacag-ctggcacgtaggcatt---gctgcct-c-----tcagtgg-cgccttggcatga-ctttacacc-agtgctaaccactgttaaaaccaaaatctgaagtattgattgcta-gtaattggcgatcttaaccaaagacaactctcaacaacggatatcttggctctcgcaacgatgaagaacgcagcgaaatgcgatacgtagtgtgaattgcagaattccgtgaaccatcgaatctttgaacgcatattgcgctcgagccctcgggcaagagcatgtctgcctcagcgtcggtttacaccctcacccctcccaacttgt--tgggtatgtttgctttttttgcaagccacaggggtggatctggcttccccaatcagccat--------caggtagattgggttggctgaagtacagaggc-ttaagcaaggacccgatatgggcttc-aactggataggtagcaccggcttttgccgactacacgaagttgttgcttgtggatcttgctagaagccaagcaggaa-tgtg-cctttggcatatcttaactttcgacctgagctcaggcaagattacccgctgaacttaagcatnnnnn

>Coelastrum_astroideum_SAG_65.81

gccagcagccgcggtaattccagctccaatagcgtatatttaagttgttgcagttaaaaagctcgtagttggatttcgggtgagttctagcggtccgcctatggtgagtactgctatggctctcctttctgtcggggacgggcttctgggcttcactgtccgggactcggagtcgacgtggttactttgagtaaattagagtgttcaaagcaggcttacgccctgaatactttagcatggaataacacgataggactctggcctatcttgttggtctgtaggaccggagtaatgattaagagggacagtcgggggcattcgtatttcattgtcagaggtgaaattcttggatttatgaaagacgaactactgcgaaagcatttgccaaggatgttttcattaatcaagaacgaaagttgggggctcgaagacgattagataccgtcgtagtctcaaccataaacgatgccgactagggattggcgaaattggtcttc-ggactga--tgcatctcgcaa-ccaa--cttgggcaat---gctgcctgg-----gttgggg-cgctacggcatgc-ct-cacacc-attgccaaccaatgtacaaaccaaaatct-tactttcgactgcta-ttaactggcggtcttaaccaaagacaactctcaacaacggatatcttggctctcgcaacgatgaagaacgcagcgaaatgcgatacgtagtgtgaattgcagaattccgtgaaccatcgaatctttgaacgcatattgcgctcgagcctccgggcaagagcatgtctggctcagcgtcggtttaacccctcactcctcctcccttgt-ggtggttggttggcttctctagctagcctttggagtggatctggcttccccaattggcc----------ttggctggttgggttggctgaagtgcagaggc-ttaagcaaggacccgatatgggcttc-aactggataggtagcaccggtttcgaccgactacacgaagttgtggcttgtggaccttgctaggagccaagcaggaa-acgc-gcttgcgcgtatc-aaactttcgacctgagctcaggcaaggctacccgctgaacttaagcatat-gg

>Coelastrum_proboscideum_SAG_217-2

gccagcagccgcggtaattccagctccaatagcgtatatttaagttgttgcagttaaaaagctcgtagttggatttcgggtgggttctagcggtccgcctatggtgagtactgctatggccttcctttctgtcggggacgggcttctgggcttcactgtccgggactcggagtcgacgtggttactttgagtaaattagagtgttcaaagcaggcttacgccctgaatactttagcatggaataacacgataggactctggcctatcttgttggtctgtaggaccggagtaatgattaagagggacagtcgggggcattcgtatttcattgtcagaggtgaaattcttggatttatgaaagacgaactactgcgaaagcatttgccaaggatgttttcattaatcaagaacgaaagttgggggctcgaagacgattagataccgtcgtagtctcaaccataaacgatgccgactagggattggcgaagcttgcccta-aaaagcaagtgcatctcacaa-ccaa--cttgggcaat---gctgcttga-----gttgggg-cgctttggcatga-ccatacacc-agtgctaaccactgtcaaaaccaaaatctgaatacgtgactgcta-ttaactggcggtcttaaccaaagacaactctcaacaacggatatcttggctctcgcaacgatgaagaacgcagcgaaatgcgatacgtagtgtgaattgcagaattccgtgaaccatcgaatctttgaacgcatattgcgctcgagcctccgggcaagagcatgtctgcctcagcgtcggtttacaccctcatccctctcacctttg-gtgagtcggttggcttctatagccagcctcagggatggatctggcttccccaagcagcct---------tgtgccggttgggttggctgaagtgtagaggc-ttaaacaaggacccaacatgggcttc-aactggataggtagcaccggcttctgccgactacacgaagttgtagcttgtggaccttgttcggagccaagcaggaa-acgc-gcttgcgcgtctc-aaactttcgacctgagctcaggcaaggctacccgctgaacttaagcatat-ca

>Coelastrum_proboscideum_var._gracile_SAG_217-3

gccagcagccgcggtaattccagctccaatagcgtatatttaagttgttgcagttaaaaagctcgtagttggatttcgggtgggttctagcggtccgcctatggtgagtactgctatggccttcctttctgtcggggacgggcttctgggcttcactgtccgggactcggagtcgacgtggttactttgagtaaattagagtgttcaaagcaggcttacgccctgaatactttagcatggaataacacgataggactctggcctatcttgttggtctgtaggaccggagtaatgattaagagggacagtcgggggcattcgtatttcattgtcagaggtgaaattcttggatttatgaaagacgaactactgcgaaagcatttgccaaggatgttttcattaatcaagaacgaaagttgggggctcgaagacgattagataccgtcgtagtctcaaccataaacgatgccgactagggattggcgaagcttgcccta-aaaagcaagtgcatctcacaa-ccaa--cttgggcaat---gctgcttga-----gttgggg-cgctttggcatga-ccatacacc-agtgctaaccactgtcaaaaccaaaatctgaatacgtgactgcta-ttaactggcggtcttaaccaaagacaactctcaacaacggatatcttggctctcgcaacgatgaagaacgcagcgaaatgcgatacgtagtgtgaattgcagaattccgtgaaccatcgaatctttgaacgcatattgcgctcgagcctccgggcaagagcatgtctgcctcagcgtcggtttacaccctcatccctctcacctttg-gtgagtcggttggcttctatagccagcctcagggatggatctggcttccccaagcagcct---------tgtgccggttgggttggctgaagtgtagaggc-ttaaacaaggacccaacatgggcttc-aactggataggtagcaccggcttctgccgactacacgaagttgtagcttgtggaccttgttcggagccaagcaggaa-acgc-gcttgcgcgtctc-aaactttcgacctgagctcaggcaaggctacccgctgaacttaagcatat-ca

>Coelastrum_pseudomicroporum_SAG_33.88

gccagcagccgcggtaattccagctccaatagcgtatatttaagttgttgcagttaaaaagctcgtagttggatttcgggtgggttctagcggtccgcctatggtgagtactgctatggccttcctttctgtcggggacgggcttctgggcttcactgtccgggactcggagtcgacgtggttactttgagtaaattagagtgttcaaagcaggcttacgccctgaatactttagcatggaataacacgataggactctggcctatcttgttggtctgtaggaccggagtaatgattaagagggacagtcgggggcattcgtatttcattgtcagaggtgaaattcttggatttatgaaagacgaactactgcgaaagcatttgccaaggatgttttcattaatcaagaacgaaagttgggggctcgaagacgattagataccgtcgtagtctcaaccataaacgatgccgactagggattggcgaaaaacaatggt-gacattgtttgtatctcacaa-ccaa--cttgggcaat---gcagcctgg-----gttgggg-cgctttggcatgc-ccatacacc-attgccaactatttttcaaaacaaaatctgaagtcttgattgttg-gtaaccagcaatcttaaccaaagacaactctcaacaacggatatcttggctctcgcaacgatgaagaacgcagcgaaatgcgatacgtagtgtgaattgcagaattccgtgaaccatcgaatctttgaacgcatattgcgctcgaggctccggccgagagcatgtctgcctcagcgtcggtttaacccctcacccctcccatctagt-atgggttggttggcttctctagtcgacctttggggtggagctggcttccccaattagt-----------ttgattgattgggttggctgaagcatagaggc-ttaagcaaggacccgaaatgggcttc-aactggataggtagcaccggcctcggccgactacacgaagttgtggcttgtggacctcgctaggagccaagcaggaa-atgc-acatgtgcatctc-taac-ttcgacctgagctcaggcaaggctacccgctgaacttaagcatat-gg

>Coelastrum_sp.

gccagcagccgcggtaattccagctccaatagcgtatatttaagttgttgcagttaaaaagctcgtagttggatttcgggtgggttctagcggtccgcctatggtgagtactgctatggnnnnnnnnnnnnnnnnnnnnnnnnnnnnnnnnnnnnnnnnnnnnnnnnnnnnnnnnnnnnnnnnnnnnnnnnnnnnnnnnnnnnnnnnnnnnnnnnnnnnnnnnnnnnnnnnnnnnnnnnnnnnnnnnnnnnnnnnnnnnnnnnnnnnnnnnnnnnnnnnnnnnnnnnnnnnnnnnnnnnnnnnnnnnnnnnnnnnnnnnnnnnnnnnnnnnnnnnnnnnnnnnnnnnnnnnnnnnnnnnnnnnnnnnnnnnnnnnnnnnnnnnnnnnnnnnnnnnnnnnnnnnnnnnnnnnnnnnnnnnnnnnnnnnnnnnnnnnnnnnnnnnnnnnnnnnnnnnnnnnnnnnnnnnnnnnnnnnnnnnnnnnnnnnnnnnnnnnnnnnnnnnnnnnnnnnnnnnnnnnnnnnnnnnnnnnnnnnnnnnnnnnnnnnnnnnnnnnnnnnnnnnnnnnnnnnnnnnnnnnnnnnnnnnnnnnnnnnnnnnnnnnnnnnnnnnnnnnnnnnnnnnnnnnnnnnnnnnnnnnnnnnnnnaaccaaagacaactctcaacaacggatatcttggctctcgcaacgatgaagaacgcagcgaaatgcgatacgtagtgtgaattgcagaattccgtgaaccatcgaatctttgaacgcatattgcgctcnnnnnnnnnnncaagagcatgtctgcctcagcgtcggtttacaccctcacccctcccaacttgt--tgggtgtgtcggcttctctagctggccattggggtggatctggcttccccaatctgcttt--------actgcggattgggttggctgaagtgcagaggc-ttaagcaaggacccgttatgggctgc-aactggataggtagcaccggcttctgccgactacacgaagttgttgcttgtggatcttgctaggagccaagcaggaa-cata-cctatggtatgtctaaactttcgacctgagctcaggcaaggctaccnnnnnnnnnnnnnnnnnnnnn

>Graesiella_emersonii_CCAP_211_8H

gccagcagccgcggtaattccagctccaatagcgtatatttaagttgttgcagttaaaaagctcgtagttggatttcgggtgggttctagcggtccgcctatggtgagtactgctatggccttcctttctgtcggggacgggcttctgggcttaattgtccgggactcggagtcgacgtggttactttgagtaaattagagtgttcaaagcaggcttacgccctgaatactttagcatggaataacacgataggactctggcctatcttgttggtctgtaggactggagtaatgattaagagggacagtcgggggcattcgtatttcattgtcagaggtgaaattcttggatttatgaaagacgaactactgcgaaagcatttgccaaggatgttttcattaatcaagaacgaaagttgggggctcgaagacgattagataccgtcgtagtctcaaccataaacgatgccgactagggattggcgaaatccgtcaac-tgcgttggccacc-ctcacag-ctca---gtgggcaat---gctgccgac-----tgagtgg-cgccttagcatgc-ttatacacc-agtgctaaccactgttgaaaccaaactctgaagttgtgattgcta-ttaattggcaatcttaaccaaagacaactctcaacaacggatatcttggctctcgcaacgatgaagaacgcagcgaaatgcgatacgtagtgtgaattgcagaattccgtgaaccatcgaatctttgaacgcatattgcgctcgagccctcgggcaagagcatgtctgcctcagcgtcggtttacaccctcacccctccctcttttt-gtgggtcagttggc--atattgccagcc-taggggtggatctggcttccccaatctgccgc--------ttggtgggttgggttggctgaagtacagaggc-ttaagcaaggacccgatatgggcttc-aactggataggtagctccggcttgtgccgactacacgaagttgtggcttgtggatcttgctaggagcccagcaggaa-cgtg-ccttaggcatgtataaacattcgacctgagctcaggcaaggctacccgctgaacttaagcatat-ca

>Scenedesmus_costatus_SAG_46.88

gccagcagccgcggtaattccagctccaatagcgtatatttaagttgttgcagttaaaaagctcgtagttggatttcgggtgggttctagcggtccgcctatggtgagtactgctatggccttcctttctgtcggggacgggcttctgggcttcactgtccgggactcggagtcgacgtggttactttgagtaaattagagtgttcaaagcaggcttacgccctgaatactttagcatggaataacacgataggactctggcctatcttgttggtctgtaggactggagtaatgattaagagggacagtcgggggcattcgtatttcattgtcagaggtgaaattcttggatttatgaaagacgaactactgcgaaagcatttgccaaggatgttttcattaatcaagaacgaaagttgggggctcgaagacgattagataccgtcgtagtctcaaccataaacgatgccgactagggattggcgaaattcggcat--aaacccgtttgta-ctcacag-ctgg---gtgggcatt---gctgcctgc-----tcagtgg-cgccttggcatga-ctttacacc-agtgctaaccactgttaaaaccaaactctgaagtattgattgcta-gtaactggcgatcttaaccaaagacaactctcaacaacggatatcttggctctcgcaacgatgaagaacgcagcgaaatgcgatacgtagtgtgaattgcagaattccgtgaaccatcgaatctttgaacgcatattgcgctcgagccctcgggcaagagcatgtctgcctcagcgtcggtttacaccctcacccctcccaacttgt--tgggtatgtttgatctatttgcaagctacaggggtggatctggcttccccaatcagccat--------caggcagattgggttggctgaagtacagaggc-ttaagcaaggacccgatatgggcttc-aactggataggtagcaccggcttctgccgactacacgaagttgttgcttgtggatcttgctagaagccaagcaggaa-tgtg-cctttggcatatctaaactttcgacctgagctcaggcaagattacccgctgaacttaannnnnnnnn

>Scenedesmus_obtusus_CCAP_276_36

gccagcagccgcggtaattccagctccaatagcgtatatttaagttgttgcagttaaaaagctcgtagttggatttcgggtgggttctagcggtccgcctatggtgagtactgctatggccttcctttctgtcggggacgggcttctgggcttcactgtccgggactcggagtcgacgtggttactttgagtaaattagagtgttcaaagcaggcttacg-ccagaatactttagcatggaataacacgataggactctggcctatcttgttggtctgtaggaccggagtaatgattaagagggacagtcgggggcattcgtatttcattgtcagaggtgaaattcttggatttatgaaagacgaactactgcgaaagcatttgccaaggatgttttcattaatcaagaacgaaagttgggggctcgaagacgattagataccgtcgtagtctcaaccataaacgatgccgactagggattggcgaaaatcgggct--agtctcgattgtagcctgcag-ctgg--tgcgagcagt---gctgcccgc----atcagtgg-cgctctggcatgc-ctatacacc-agtgctaaccactgtcaaaaccaaactctgaagttttgattgcta-gtaactggcaatcctaaccaaagacaactctcaacaacggatatcttggctctcgcaacgatgaagaacgcagcgaaatgcgatacgtagtgtgaattgcagaattccgtgaaccatcgaatctttgaacgcatattgcgctcgagccctcgggcaagagcatgtctgcctcagcgtcggtttacaccctcacccctctttcctttt-ggatcgcaggtcagcttttcagctggccctaggggtggatctggctttcccaatcctttct-------------gggttgggttggctgaagtgtagaggc-ttaatcaaggacccgatatgggcttc-aactggataggtagcaacggcttctgccgactacacgaagttgtggcttgtggactttgataggagccaagcaggaa-acgt-gcttgcacgttct-aaacnnnnnnnnnnnnnnnnnnnnnnnnnnnnnnnnnnnnnnnnnnnnnnnnn

>Scenedesmus_obtusus_SAG_52.80

gccagcagccgcggtaattccagctccaatagcgtatatttaagttgttgcagttaaaaagctcgtagttggatttcgggtgggttctagcggtccgcctatggtgagtactgctatggccttcctttctgtcggggacgggcttctgggcttcactgtccgggactcggagtcgacgtggttactttgagtaaattagagtgttcaaagcaggcttacg-ccagaatactttagcatggaataacacgataggactctggcctatcttgttggtctgtaggaccggagtaatgattaagagggacagtcgggggcattcgtatttcattgtcagaggtgaaattcttggatttatgaaagacgaactactgcgaaagcatttgccaaggatgttttcattaatcaagaacgaaagttgggggctcgaagacgattagataccgtcgtagtctcaaccataaacgatgccgactagggattggcgaaaatcgggct--agtctcgattgtagcctgcag-ctgg--tgcgagcagt---gctgcccgc----atcagtgg-cgctctggcatgc-ctatacacc-agtgctaaccactgtcaaaaccaaactctgaagttttgattgcta-gtaactggcaatcctaaccaaagacaactctcaacaacggatatcttggctctcgcaacgatgaagaacgcagcgaaatgcgatacgtagtgtgaattgcagaattccgtgaaccatcgaatctttgaacgcatattgcgctcgagccctcgggcaagagcatgtctgcctcagcgtcggtttacaccctcacccctctttcctttt-ggatcgcaggtcagcttttcagctggccctaggggtggatctggctttcccaatcctttct-------------gggttgggttggctgaagtgtagaggc-ttaatcaaggacccgatatgggcttc-aactggataggtagcaacggcttctgccgactacacgaagttgtggcttgtggactttgataggagccaagcaggaa-acgt-gcttgcacgttct-aaannnnnnnnnnnnnnnnnnnnnnnnnnnnnnnnnnnnnnnnnnnnnnnnnn

>Scenedesmus_rubescens_CCALA_475

gccagcagccgcggtaattccagctccaatagcgtatatttaagttgttgcagttaaaaagctcgtagttggatttcgggtgggttctagcggtccgcctatggtgagtactgctatggccttcctttctgtcggggacgggcttctgggcttaactgtccgggactcggagtcgacgtggttactttgagtaaattagagtgttcaaagcaggcttacgccctgaatactttagcatggaataacacgataggactctggcctatcttgttggtctgtaggactggagtaatgattaagagggacagtcgggggcattcgtatttcattgtcagaggtgaaattcttggatttatgaaagacgaactactgcgaaagcatttgccaaggatgttttcattaatcaagaacgaaagttgggggctcgaagacgattagataccgtcgtagtctcaaccataaacgatgccgactagggattggcgaaattcggc-ca-aaacccgtttgta-ctcacag-ctga---gtgggcatt---gcgacctgc-----tcagtgg-cgccttggcatga-ctttacacc-agtgctaaccactgttaaaaccaaactctgaagtattggttgctg-gtaactggcaatcttaaccaaagacaactctcaacaacggatatcttggctctcgcaacgatgaagaacgcagcgaaatgcgatacgtagtgtgaattgcagaattccgtgaaccatcgaatctttgaacgcatattgcgctcaagccctcgggcaagagcatgtctgcctcagcgtcggtttacaccctcacccctcccaacttgt--tgggtgtgcttgctttattggcaagccgttggggtggatctggcttccccaatcagccat--------caggcagattgggttggctgaagtgcagaggc-ttaagcaaggacccgatatgggcttc-aactggataggtagcaccggcttctgccgactacacgaagttgttgcttgtggctcttgctagaggccaagcaggaa-cgtg-cctttggcatgtctaaactttcgacctgagctcaggcaagattacccgctgaacttaagcatat-ca

>Scenedesmus_sp._Ki4

gccagcagccgcggtaattccagctccaatagcgtatatttaagttgttgcagttaaaaagctcgtagttggatttcgggtgggttctagcggtccgcctatggtgagtactgctatggcctatctttctgtcggggacgggcttctgggcttaactgtccgggactcggagtcgacgtggttactttgagtaaattagagtgttcaaagcaggcttacgccctgaatactttagcatggaataacacgataggactctggcctatcttgttggtctgtaggactggagtaatgattaagagggacagtcgggggcattcgtatttcattgtcagaggtgaaattcttggatttatgaaagacgaactactgcgaaagcatttgccaaggatgttttcattaatcaagaacgaaagttgggggctcgaagacgattagataccgtcgtagtctcaaccataaacgatgccgactagggattggcgaagttcggctta-aaacccgttcgcatctcacag-ctgg---gcgggcatt---gctgcctgc-----tcagtgg-cgccttggcatga-ctatacacc-agtgctaaccactgttaaaactaaactctgaagttgtgattgcta-gtaactggcaatcttaaccaaagacaactctcaacaacggatatcttggctctcgcaacgatgaagaacgcagcgaaatgcgatacgtagtgtgaattgcagaattccgtgaaccatcgaatctttgaacgcatattgcgctcgagccttcgggcaagagcatgtctgcctcagcgtcggtttacaccctcacccctcccaacttgt--tgggtgtgtcggcttctctagctggccattggggtggatctggcttccccaatctgcttt--------actgcggattgggttggctgaagtgcagaggc-ttaagcaaggacccgttatgggctgc-aactggataggtagcaccggcttctgccgactacacgaagttgttgcttgtggatcttgctaggagccaagcaggaa-cata-cctatggtatgtctaaactttcgacctgagctcaggcaaggctacccgctgaacttaannnnnnnnn

>Scenedesmus_vacuolatus_SAG_211-8b

gccagcagccgcggtaattccagctccaatagcgtatatttaagttgttgcagttaaaaagctcgtagttggatttcgggtgggttctagcggtccgcctatggtgagtactgctatggccttcctttctgtcggggacgggcttctgggcttaattgtccgggactcggagtcgacgtggttactttgagtaaattagagtgttcaaagcaggcttacgccctgaatactttagcatggaataacacgataggactctggcctatcttgttggtctgtaggactggagtaatgattaagagggacagtcgggggcattcgtatttcattgtcagaggtgaaattcttggatttatgaaagacgaactactgcgaaagcatttgccaaggatgttttcattaatcaagaacgaaagttgggggctcgaagacgattagataccgtcgtagtctcaaccataaacgatgccgactagggattggcgaaatccgtcaac-tgcgttggccacc-ctcacag-ctca---gtgggcaat---gctgccgac-----tgagtgg-cgccttagcatgc-ttatacacc-agtgctaaccactgttgaaaccaaactctgaagttgtgattgcta-ttaattggcaatcttaaccaaagacaactctcaacaacggatatcttggctctcgcaacgatgaagaacgcagcgaaatgcgatacgtagtgtgaattgcagaattccgtgaaccatcgaatctttgaacgcatattgcgctcgagccctcgggcaagagcatgtctgcctcagcgtcggtttacaccctcacccctccctcttttt-gtgggtcagttggc--atattgccagcc-taggggtggatctggcttccccaatctgccgc--------ttggtgggttgggttggctgaagtacagaggctttaagcaaggacccgatatgggcttc-aactggataggtagctccggcttgtgccgactacacgaagttgtggcttgtggatcttgctaggagcccagcaggaa-cgtg-ccttaggcatgtataaacattcgacctgagctcaggcaaggctacccgctgaacttaannnnnnnnn

>Scotiellopsis_reticulata_CCALA_474

gccagcagccgcggtaattccagctccaatagcgtatatttaagttgttgcagttaaaaagctcgtagttggatttcgggtgggttctagcggtccgcctatggtgagtactgctatggccttcctttctgtcggggacgggcttctgggcttcactgtccgggactcggagtcgacgtggttactttgagtaaattagagtgttcaaagcaggcttacg-ccagaatactttagcatggaataacacgataggactctggcctatcttgttggtctgtaggaccggagtaatgattaagagggacagtcgggggcattcgtatttcattgtcagaggtgaaattcttggatttatgaaagacgaactactgcgaaagcatttgccaaggatgttttcattaatcaagaacgaaagttgggggctcgaagacgattagataccgtcgtagtctcaaccataaacgatgccgactagggattggcgaaaatcaggat--tcccctggttgta-cctgcaa-gctg---gtgcaagta---ttctattacttgtatcaatgg-cgctctggcatgc-tatcacacc-agtgccaaccacagttaaatcaaaactctgaagctttgattgcta-gtaactggcaatcttaaccaaaaacaactctcaacaacggatatcttggctctcgcaacgatgaagaacgcagcgaaatgcgatacgtagtgtgaattgcagaattccgtgaaccatcgaatctttgaacgcatattgcgctcgagccctcgggcaagagcatgtctgcctcagcgtcggtttataccctcacccctcctccctcag--gagagctggccaacat-tcagttggcctcaggggtggatctggctttcccaatttgtt----------cactccgattgggttggctgaagcttagaggc--taagcaaggacccgatatgggcttc-aactggataggtagcaccggcgtctgccgactacacgaagttgtggcttgtggactttgctagaggccaagcaggaa-acat-gctgtgcatgtcttaaactttcgacctgagctcaggcaaggctacccgctgaacttaagcatat-ca

>Coelastrella multistriata Ch23

gccagcagccgcggtaataccagctccaatagcgtatatttaagttgttgcagttaaaaagctcgtagttggatttcgggtgggttctagcggtccgcctatggtgagtactgctatggccttcctttctgtcggggacgggcttctgggcttcactgtccgggactcggagtcgacgtggttactttgagtaaattagagtgttcaaagcaggcttacgccctgaatactttagcatggaataacacgataggactctggcctatcttgttggtctgtaggactggagtaatgattaagagggacagtcgggggcattcgtatttcattgtcagaggtgaaattcttggatttatgaaagacgaactactgcgaaagcatttgccaaggatgttttcattaatcaagaacgaaagttgggggctcgaagacgattagataccgtcgtagtctcaaccataaacgatgccgactagggattggcgaaattcggcata-aaacccgtttgtg-ctcacagactga---gtggacgtt---gatgtctac-----tcagtgg-cgccttggcatgt-ctatacacc-aatgctaaccattgtt-aaaccaaactctgaagcattgattgcta-gcaactggcaatcttaaccaaagacaactctcaacaacggatatcttggctctcgcaacgatgaagaacgcagcgaaatgcgatacgtagtgtgaattgcagaattccgtgaaccatcgaatctttgaacgcatattgcgctcgagccctcgggcaagagcatgtctgcctcagcgtcggtttacaccctcacccctcccaacttgt--tgggcgtgtttgctctgtttgcaagccattggggtggatctggcttccccaatcatgtccgtttcactgcggcagactgggctggctgaagtgcagaggc-ttaagcaaggacccgatatgggcttc-aactggataggtagcaccggctcttgccgactacacgaagttgttgcttgtggatcttgctaggagccaagcaggaa-cgtg-cctttggcatgtct-aactttcgacctgagctcaggcaagactacccgctgaacttaagcatat-ca
