# Supplementary material for: Lipid accumulation by Coelastrella multistriata (Scenedesmaceae, Sphaeropleales) during nitrogen and phosphorus starvation
Source: Sci Rep. 2021 Oct 6;11:19818. doi: 10.1038/s41598-021-99376-9 (PMC8494790; doi:10.1038/s41598-021-99376-9)
Supplement: Supplementary file 3 — Supplementary Information 3. [file 41598_2021_99376_MOESM3_ESM.docx]

#NEXUS

begin taxa;

dimensions ntax=34;

taxlabels

'01_Acutodesmus_obliquus_SAG_22.81_AB037096'

02_Chlorella_emersonii_CCAP_211_15_FR865661

'03_Chlorella_fusca_var._vacuolata_UTEX252'

04_Coelastrella_aeroterrestrica_SWK1_2_JX513879

05_Coelastrella_corcontica_CCALA_308_AB037082

06_Coelastrella_oocystiformis_FACHB_2312_MH176105

07_Coelastrella_oocystiformis_SAG_277_1_AB012848

08_Coelastrella_rubescens_FACHB_2295_MH176095

09_Coelastrella_rubescens_IPPAS_H_350_KT962984

10_Coelastrella_saipanensis_LY31_2_MF407353

'11_Coelastrella_sp._FI69_KY368609'

'12_Coelastrella_sp._M_60_KF002250'

'13_Coelastrella_sp._QW_2019b_FACHB_2314_MH176108'

'14_Coelastrella_sp._QW_2019c_FACHB_2300_MH176090'

'15_Coelastrella_sp._QW_2019e_FACHB_2311_MH176103'

'16_Coelastrella_sp._SAG_2123_JX513883'

17_Coelastrella_striolata_CAUP_H_3602_JX513881

'18_Coelastrella_striolata_var._multistriata_CCALA_309_JX513880'

19_Coelastrella_terrestris_CAUP_H_4403

20_Coelastrella_terrestris_CCALA_476_JX513882

'21_Coelastrum_astroideum_SAG_65.81_AF388380'

22_Coelastrum_proboscideum_SAG_217_2_KF673364

'23_Coelastrum_proboscideum_var._gracile_SAG_217_3_GQ375099'

'24_Coelastrum_pseudomicroporum_SAG_33.88_AF388381'

'25_Coelastrum_sp._DOE0202'

26_Graesiella_emersonii_CCAP_211_8H_MG022718

'27_Scenedesmus_costatus_SAG_46.88_AB773883'

28_Scenedesmus_obtusus_CCAP_276_36_AB037091

'29_Scenedesmus_obtusus_SAG_52.80_X81966'

30_Scenedesmus_rubescens_CCALA_475_JX513884

'31_Scenedesmus_sp._Ki4_AB734096'

32_Scenedesmus_vacuolatus_SAG_211_8b_X56104

33_Scotiellopsis_reticulata_CCALA_474_JX513885

Ch23

;

end;

begin trees;

tree TREE1 = [&R] ((((22_Coelastrum_proboscideum_SAG_217_2_KF673364[&length_range={1.5937447305763824E-7,0.053034842561658975},height_95%_HPD={0.0,2.7755575615628914E-17},length_95%_HPD={1.5937447305763824E-7,0.0015071586994139415},rate=1.0,length=5.069628362201457E-4,length_median=3.5796085066005774E-4,height_median=1.3877787807814457E-17,height_range={0.0,8.881784197001252E-16},height=1.2322155954508776E-17]:3.58E-4,'23_Coelastrum_proboscideum_var._gracile_SAG_217_3_GQ375099'[&length_range={1.5937447305763824E-7,0.053034842561658975},height_95%_HPD={0.0,2.7755575615628914E-17},length_95%_HPD={1.5937447305763824E-7,0.0015071586994139415},rate=1.0,length=5.069628362201457E-4,length_median=3.5796085066005774E-4,height_median=1.3877787807814457E-17,height_range={0.0,8.881784197001252E-16},height=1.2322155954508776E-17]:3.58E-4)[&length_range={0.03472899293960988,2.6210678197588213},height_95%_HPD={1.5937447306324515E-7,0.001507158699413938},length_95%_HPD={0.0448198371633782,0.06939634598262209},rate=1.0,length=0.057033242365447115,posterior=1.0,length_median=0.05656898128674955,height_median=3.5796085066007455E-4,height_range={1.5937447306324515E-7,0.0530348425616598},height=5.069628362201586E-4]:0.056703,('21_Coelastrum_astroideum_SAG_65.81_AF388380'[&length_range={0.024474400965095136,2.718650761711503},height_95%_HPD={0.0,2.7755575615628914E-17},length_95%_HPD={0.03369141355927721,0.0550204576961425},rate=1.0,length=0.04416110569485315,length_median=0.043744544129570996,height_median=1.3877787807814457E-17,height_range={0.0,8.881784197001252E-16},height=1.2326736036692751E-17]:0.04374,'24_Coelastrum_pseudomicroporum_SAG_33.88_AF388381'[&length_range={0.024474400965095136,2.9225037728011944},height_95%_HPD={0.0,2.7755575615628914E-17},length_95%_HPD={0.033772791881915676,0.05514058576869028},rate=1.0,length=0.04418014817927122,length_median=0.043747556590006655,height_median=1.3877787807814457E-17,height_range={0.0,8.881784197001252E-16},height=1.2317853453063223E-17]:0.04374)[&length_range={2.2163547909681558E-4,0.693526334418942},height_95%_HPD={0.03369097641642596,0.05501138485133198},length_95%_HPD={0.004647274998824828,0.022362485843610586},rate=1.0,length=0.013393766571395538,posterior=0.9988298946905222,length_median=0.013009330107117266,height_median=0.04373986877728682,height_range={0.02447440096509515,1.5175820711788384},height=0.04408105854032908]:0.013321)[&length_range={0.003631507364806455,0.39974330182521556},height_95%_HPD={0.04550112773953764,0.07001061269942638},length_95%_HPD={0.01222541575556449,0.036379058652269924},rate=1.0,length=0.024245605159725726,posterior=0.999939994599514,length_median=0.023881797494965926,height_median=0.05706065134852526,height_range={0.035437429994638045,1.3670164755579557},height=0.05741681531568719]:0.02293,((10_Coelastrella_saipanensis_LY31_2_MF407353[&length_range={3.369548733101656E-7,2.430139599668294},height_95%_HPD={0.0,2.7755575615628914E-17},length_95%_HPD={3.369548733101656E-7,0.054266366739980636},rate=1.0,length=0.034211531357055465,length_median=0.04314859540047912,height_median=1.3877787807814457E-17,height_range={0.0,1.7763568394002505E-15},height=1.2326874827061962E-17]:0.045154,(09_Coelastrella_rubescens_IPPAS_H_350_KT962984[&length_range={1.529366983922531E-8,1.500498956522538},height_95%_HPD={0.0,2.7755575615628914E-17},length_95%_HPD={1.529366983922531E-8,0.018685025651063274},rate=1.0,length=0.006023507668080507,length_median=0.003951646377117856,height_median=1.3877787807814457E-17,height_range={0.0,8.881784197001252E-16},height=1.2280310658191546E-17]:0.032029,(((((30_Scenedesmus_rubescens_CCALA_475_JX513884[&length_range={1.7322243105861303E-8,0.01851902570896561},height_95%_HPD={0.0,2.7755575615628914E-17},length_95%_HPD={1.7322243105861303E-8,0.0014753374286186958},rate=1.0,length=4.944791740248296E-4,length_median=3.476062770573644E-4,height_median=1.3877787807814457E-17,height_range={0.0,1.7763568394002505E-15},height=1.2337422895122027E-17]:3.41E-4,08_Coelastrella_rubescens_FACHB_2295_MH176095[&length_range={1.7322243105861303E-8,0.01851902570896561},height_95%_HPD={0.0,2.7755575615628914E-17},length_95%_HPD={1.7322243105861303E-8,0.0014728757489011059},rate=1.0,length=4.945925869214547E-4,length_median=3.4841077895478046E-4,height_median=1.3877787807814457E-17,height_range={0.0,1.7763568394002505E-15},height=1.2344084832844172E-17]:3.41E-4)[&length_range={3.767002998357645E-8,0.9424087007667293},height_95%_HPD={1.7322243112127822E-8,0.0014463047438871418},length_95%_HPD={7.197310120755836E-7,0.019744307358561558},rate=1.0,length=0.013529366199589013,posterior=0.9572561530537749,length_median=0.014746227314857002,height_median=3.4131087673089366E-4,height_range={1.7322243112127822E-8,0.01851902570896602},height=4.85042019942331E-4]:0.01486,('27_Scenedesmus_costatus_SAG_46.88_AB773883'[&length_range={0.0011538850272846677,0.30920417968757613},height_95%_HPD={0.0,2.7755575615628914E-17},length_95%_HPD={0.0029743675171372912,0.009776524810290548},rate=1.0,length=0.0063448853371216715,length_median=0.006155306412463186,height_median=1.3877787807814457E-17,height_range={0.0,1.7763568394002505E-15},height=1.2334369506999376E-17]:0.006155,(20_Coelastrella_terrestris_CCALA_476_JX513882[&length_range={6.093819395835092E-8,0.30920417968757613},height_95%_HPD={0.0,2.7755575615628914E-17},length_95%_HPD={6.093819395835092E-8,0.0013396234604612942},rate=1.0,length=4.66873343741141E-4,length_median=3.217343970841008E-4,height_median=1.3877787807814457E-17,height_range={0.0,1.7763568394002505E-15},height=1.2358241450503733E-17]:3.22E-4,19_Coelastrella_terrestris_CAUP_H_4403[&length_range={6.093819395835092E-8,1.3161418365363544},height_95%_HPD={0.0,2.7755575615628914E-17},length_95%_HPD={6.093819395835092E-8,0.0013396234604612942},rate=1.0,length=4.985451670070615E-4,length_median=3.217343970841008E-4,height_median=1.3877787807814457E-17,height_range={0.0,1.7763568394002505E-15},height=1.2367124034133261E-17]:3.22E-4)[&length_range={6.349104822990844E-4,0.21411736947593646},height_95%_HPD={6.093819396313371E-8,0.00133940217584462},length_95%_HPD={0.002632031209454745,0.009356763040877179},rate=1.0,length=0.005877780318502757,posterior=0.9999599963996759,length_median=0.005705878308045337,height_median=3.2171757556088976E-4,height_range={6.093819396313371E-8,0.11769653250954715},height=4.5583059410409356E-4]:0.005833)[&length_range={2.4242260123587094E-4,0.5209290186636186},height_95%_HPD={0.0029739716718298753,0.009774144027792225},length_95%_HPD={0.004727791588086115,0.014588268876928136},rate=1.0,length=0.009552754919192202,posterior=0.9994999549959497,length_median=0.009401145495825654,height_median=0.006155133587595077,height_range={0.0011538850272846957,0.40739704706227187},height=0.00633814060477445]:0.009046)[&length_range={7.432216854105778E-9,0.014323878597050666},height_95%_HPD={0.010712764346670789,0.02007072143609316},length_95%_HPD={3.811413507998074E-7,0.007161581310365341},rate=1.0,length=0.003195155244399696,posterior=0.5227770499344941,length_median=0.0028814623618137677,height_median=0.015201493859430495,height_range={0.007293055964298148,0.029253461930870306},height=0.015304946056258224]:0.002885,(06_Coelastrella_oocystiformis_FACHB_2312_MH176105[&length_range={5.320204505378704E-4,0.07362807215264944},height_95%_HPD={0.0,2.7755575615628914E-17},length_95%_HPD={0.0015237958084057398,0.006164009823327216},rate=1.0,length=0.003704170117378725,length_median=0.003568525417030827,height_median=1.3877787807814457E-17,height_range={0.0,1.7763568394002505E-15},height=1.2428261191770871E-17]:0.003569,07_Coelastrella_oocystiformis_SAG_277_1_AB012848[&length_range={5.320204505378704E-4,0.07362807215264944},height_95%_HPD={0.0,2.7755575615628914E-17},length_95%_HPD={0.0015237958084057398,0.006164009823327216},rate=1.0,length=0.003704170117378725,length_median=0.003568525417030827,height_median=1.3877787807814457E-17,height_range={0.0,1.7763568394002505E-15},height=1.2428261191770871E-17]:0.003569)[&length_range={2.0963670493157754E-4,0.8872996543230455},height_95%_HPD={0.0015237958084057535,0.00616400982332721},length_95%_HPD={0.009117879561090537,0.019254759472049073},rate=1.0,length=0.014162264809444984,posterior=1.0,length_median=0.013978849580753516,height_median=0.0035685254170308434,height_range={5.320204505378789E-4,0.07362807215265033},height=0.0037041701173787366]:0.014518)[&length_range={2.6548743299256716E-6,0.018218301787277527},height_95%_HPD={0.01352755993393269,0.022967392380737583},length_95%_HPD={4.4963433728045094E-4,0.008237306028763514},rate=1.0,length=0.004231784608955742,posterior=0.7702693242391815,length_median=0.0040043122179669495,height_median=0.018086960272048258,height_range={0.009646072751700291,0.03120863339326964},height=0.01819673069993927]:0.004289,(04_Coelastrella_aeroterrestrica_SWK1_2_JX513879[&length_range={5.49929981286558E-4,1.0809207450307314},height_95%_HPD={0.0,2.7755575615628914E-17},length_95%_HPD={0.012765755860184434,0.02267493619216079},rate=1.0,length=0.017663831489167996,length_median=0.017487750683322714,height_median=1.3877787807814457E-17,height_range={0.0,1.7763568394002505E-15},height=1.2365666735256542E-17]:0.017419,((05_Coelastrella_corcontica_CCALA_308_AB037082[&length_range={0.0011076858845960541,0.5696127859434478},height_95%_HPD={0.0,2.7755575615628914E-17},length_95%_HPD={0.0027602264343211647,0.007931739329639973},rate=1.0,length=0.005231926940156525,length_median=0.005104889600007493,height_median=1.3877787807814457E-17,height_range={0.0,1.7763568394002505E-15},height=1.2320351679709028E-17]:0.005093,(Ch23[&length_range={1.0414923256178228E-6,0.7892082415848144},height_95%_HPD={0.0,2.7755575615628914E-17},length_95%_HPD={2.0778436779504332E-5,0.0016730228622817425},rate=1.0,length=7.323439558982169E-4,length_median=5.992837620844168E-4,height_median=1.3877787807814457E-17,height_range={0.0,8.881784197001252E-16},height=1.2247000969580817E-17]:5.99E-4,'18_Coelastrella_striolata_var._multistriata_CCALA_309_JX513880'[&length_range={1.0414923256178228E-6,0.5696127859434478},height_95%_HPD={0.0,2.7755575615628914E-17},length_95%_HPD={2.0778436779504332E-5,0.0016730228622817425},rate=1.0,length=7.31333250521166E-4,length_median=5.992837620844168E-4,height_median=1.3877787807814457E-17,height_range={0.0,1.7763568394002505E-15},height=1.2278090012284165E-17]:5.99E-4)[&length_range={1.9254463256543277E-4,0.07550885873679457},height_95%_HPD={1.933334859849367E-5,0.0016708196017321125},length_95%_HPD={0.002083466211703868,0.007149907127015388},rate=1.0,length=0.004505364919159523,posterior=0.999909991899271,length_median=0.004390279645777194,height_median=5.992393080126862E-4,height_range={1.0414923256318875E-6,0.05235305288011438},height=7.117262098709666E-4]:0.004494)[&length_range={4.977812829495311E-6,0.13311006894262867},height_95%_HPD={0.0027607564731950834,0.007886052041077174},length_95%_HPD={3.495473607606032E-4,0.005352401883415574},rate=1.0,length=0.002765636439491434,posterior=0.9902591233210989,length_median=0.0025881912888864374,height_median=0.00509343155985012,height_range={0.0014353213916046548,0.12786191161690885},height=0.0051991991693572]:0.002739,17_Coelastrella_striolata_CAUP_H_3602_JX513881[&length_range={1.1664410097226548E-4,0.7892082415848144},height_95%_HPD={0.0,2.7755575615628914E-17},length_95%_HPD={0.004877470231153205,0.011141617084357363},rate=1.0,length=0.007969896570410273,length_median=0.00782451187919375,height_median=1.3877787807814457E-17,height_range={0.0,8.881784197001252E-16},height=1.2295993969912432E-17]:0.007832)[&length_range={5.714733581893561E-6,0.2653178943652854},height_95%_HPD={0.004912256713997323,0.011154450758720902},length_95%_HPD={0.005276669451644269,0.014531184944427768},rate=1.0,length=0.009761700444445005,posterior=0.9974697722795052,length_median=0.009565861020663244,height_median=0.007832431621832996,height_range={0.0027569929162026136,0.33471021346584795},height=0.007964712074061995,!rotate=false]:0.009586)[&length_range={1.952899272531297E-5,0.017382273729233445},height_95%_HPD={0.012874646545837351,0.022562105538873112},length_95%_HPD={0.0013030050351221928,0.008988340062038726},rate=1.0,length=0.004910847128319727,posterior=0.9644968047124242,length_median=0.004708467382565435,height_median=0.01741870314512796,height_range={0.00888990230080794,0.02962995123663975},height=0.01753658187677196]:0.004957)[&length_range={1.1710942020385154E-4,0.0238121236282772},height_95%_HPD={0.01752215727087736,0.027730588824140645},length_95%_HPD={0.0032494785557402213,0.014437167042513608},rate=1.0,length=0.008642916868525691,posterior=0.7875308777790001,length_median=0.008419054423959751,height_median=0.022375465066925795,height_range={0.0131817390077541,0.03615149680763641},height=0.022494118670104896]:0.009561,((('16_Coelastrella_sp._SAG_2123_JX513883'[&length_range={0.004694432980644049,2.7561699478739468},height_95%_HPD={0.0,2.7755575615628914E-17},length_95%_HPD={0.008053839454394366,0.017859014784816694},rate=1.0,length=0.012984872968614709,length_median=0.012621556055599087,height_median=1.3877787807814457E-17,height_range={0.0,1.7763568394002505E-15},height=1.231341216124846E-17]:0.01262,('14_Coelastrella_sp._QW_2019c_FACHB_2300_MH176090'[&length_range={2.3927113041355258E-5,1.757986990750982},height_95%_HPD={0.0,2.7755575615628914E-17},length_95%_HPD={5.123549759232105E-4,0.003912804886891193},rate=1.0,length=0.0021453415897609274,length_median=0.001940431630783167,height_median=1.3877787807814457E-17,height_range={0.0,8.881784197001252E-16},height=1.2280380053376152E-17]:0.001944,('31_Scenedesmus_sp._Ki4_AB734096'[&length_range={7.183937515525012E-9,0.04900278734097188},height_95%_HPD={0.0,2.7755575615628914E-17},length_95%_HPD={7.183937515525012E-9,0.0015972125652754616},rate=1.0,length=5.588275091194539E-4,length_median=4.0777452575603985E-4,height_median=1.3877787807814457E-17,height_range={0.0,8.881784197001252E-16},height=1.2290928121436217E-17]:4.05E-4,'25_Coelastrum_sp._DOE0202'[&length_range={7.183937515525012E-9,0.04900278734097188},height_95%_HPD={0.0,2.7755575615628914E-17},length_95%_HPD={7.662495712276808E-9,0.0015946090664940243},rate=1.0,length=5.578680710079091E-4,length_median=4.073338283983306E-4,height_median=1.3877787807814457E-17,height_range={0.0,8.881784197001252E-16},height=1.2291066911805428E-17]:4.05E-4)[&length_range={1.8621799771380548E-6,1.6710091503997544},height_95%_HPD={7.662495721261209E-9,0.0015736841606014546},length_95%_HPD={9.081269536423842E-5,0.0031899052501853306},rate=1.0,length=0.0015797132370144972,posterior=0.9946995229570661,length_median=0.0013800347185655671,height_median=4.048580413488073E-4,height_range={7.1839375281879114E-9,0.04900278734097263},height=5.528141975243504E-4]:0.001539)[&length_range={0.0023916917429195788,0.5960797357939976},height_95%_HPD={5.228911079151466E-4,0.003915306192757678},length_95%_HPD={0.00617107785230212,0.0158270391207565},rate=1.0,length=0.010770303887190923,posterior=0.999949995499595,length_median=0.01052716908974705,height_median=0.0019442849874888174,height_range={1.1936879625991381E-4,0.4761957993845529},height=0.0020844068800872253]:0.010676)[&length_range={6.247701146369945E-4,0.034211018540560495},height_95%_HPD={0.008047385436065382,0.017841555653912877},length_95%_HPD={0.0028149914171848884,0.014122562093060152},rate=1.0,length=0.00811054409249632,posterior=0.9997299756978129,length_median=0.007780336346345428,height_median=0.012620191994870765,height_range={0.004694432980644064,0.0484702102220092},height=0.012810682128139631]:0.007388,'15_Coelastrella_sp._QW_2019e_FACHB_2311_MH176103'[&length_range={1.529366983922531E-8,1.1103663567219084},height_95%_HPD={0.0,2.7755575615628914E-17},length_95%_HPD={8.63502298923107E-7,0.02532726592425221},rate=1.0,length=0.014225704932102973,length_median=0.01699469284378618,height_median=1.3877787807814457E-17,height_range={0.0,1.7763568394002505E-15},height=1.2320906841185874E-17]:0.020008)[&length_range={5.805038661224615E-7,0.027397126157343445},height_95%_HPD={0.014143678846041313,0.026785047592455244},length_95%_HPD={3.1680115281720878E-6,0.015234638140656547},rate=1.0,length=0.0076321159670745585,posterior=0.4398595873628627,length_median=0.007418729867094369,height_median=0.020008359182361664,height_range={0.010336574672789797,0.03574514366943762},height=0.02019278221796752]:0.005102,('12_Coelastrella_sp._M_60_KF002250'[&length_range={0.0016283554354348216,7.374693389110324},height_95%_HPD={0.0,2.7755575615628914E-17},length_95%_HPD={0.006304517444933595,0.027874978733110612},rate=1.0,length=0.01685978202909773,length_median=0.01573988895963247,height_median=1.3877787807814457E-17,height_range={0.0,8.881784197001252E-16},height=1.2327707569277231E-17]:0.015371,'11_Coelastrella_sp._FI69_KY368609'[&length_range={0.0016283554354348216,6.917080055625351},height_95%_HPD={0.0,2.7755575615628914E-17},length_95%_HPD={0.006355684703214226,0.026491953197300723},rate=1.0,length=0.016384602287785995,length_median=0.015594529137061245,height_median=1.3877787807814457E-17,height_range={0.0,8.881784197001252E-16},height=1.2321878373770353E-17]:0.015371)[&length_range={1.1331032756661807E-6,0.05100434344910021},height_95%_HPD={0.006474313330971371,0.025713595645425316},length_95%_HPD={9.311574668118897E-4,0.023062140767734912},rate=1.0,length=0.011365691633439147,posterior=0.936924323189087,length_median=0.010632776199659497,height_median=0.015371067885639522,height_range={0.001628355435434825,0.04418242441347027},height=0.015832424903262306]:0.009739)[&length_range={1.7167750079871702E-7,0.023944689259835315},height_95%_HPD={0.01776598729138383,0.03362764768957513},length_95%_HPD={4.897594024569352E-7,0.013163149729538536},rate=1.0,length=0.006347365032675488,posterior=0.3781940374633717,length_median=0.006010137630752919,height_median=0.02510991926236953,height_range={0.01246568762692532,0.04478028131861496},height=0.02537857414090678]:0.006826)[&length_range={1.9564017933459965E-6,0.041022372483510997},height_95%_HPD={0.025034827097392617,0.040028447001030434},length_95%_HPD={0.004726241939484614,0.025740113029586675},rate=1.0,length=0.014801132970152309,posterior=0.34586112750147513,length_median=0.014462646044991881,height_median=0.031936311059360414,height_range={0.0193407942245324,0.06110449165892454},height=0.032296306501895924]:9.3E-5)[&length_range={1.4392993698895962E-5,0.04270642812016451},height_95%_HPD={0.02500059179633636,0.040337811124738575},length_95%_HPD={0.0048985857522548665,0.025828534086631008},rate=1.0,length=0.014788958933371682,posterior=0.6486183756538089,length_median=0.014456854454424586,height_median=0.03202900343519266,height_range={0.02022030511217289,0.23058996029529777},height=0.0323885260594466]:0.013125)[&length_range={6.784665961059866E-7,0.05075758721549012},height_95%_HPD={0.036255092777327136,0.05510994277056873},length_95%_HPD={6.300862166421853E-4,0.01449583216656497},rate=1.0,length=0.007476514339318398,posterior=0.6517486573791641,length_median=0.007134564862965542,height_median=0.04515397280707073,height_range={0.027573817002904864,0.2612043031909996},height=0.0453980220331234]:0.008492,(((32_Scenedesmus_vacuolatus_SAG_211_8b_X56104[&length_range={7.140706990491558E-9,2.3435208199922886},height_95%_HPD={0.0,2.7755575615628914E-17},length_95%_HPD={7.140706990491558E-9,0.0014877676906164363},rate=1.0,length=5.882189385197197E-4,length_median=3.3265584223238166E-4,height_median=1.3877787807814457E-17,height_range={0.0,1.7763568394002505E-15},height=1.2301268003942464E-17]:2.19E-4,26_Graesiella_emersonii_CCAP_211_8H_MG022718[&length_range={3.2455769773609643E-9,0.5405949541869773},height_95%_HPD={0.0,2.7755575615628914E-17},length_95%_HPD={3.2455769773609643E-9,0.001484621668911901},rate=1.0,length=5.128256455648065E-4,length_median=3.363751973204111E-4,height_median=1.3877787807814457E-17,height_range={0.0,1.7763568394002505E-15},height=1.2311746676817923E-17]:2.19E-4)[&length_range={9.15773580568947E-8,1.23339135788605},height_95%_HPD={3.4032854673293755E-8,9.650078650992877E-4},length_95%_HPD={9.15773580568947E-8,0.0014727600379886173},rate=1.0,length=5.623013852815548E-4,posterior=0.22547029232630936,length_median=3.423707184939291E-4,height_median=2.1870846327347315E-4,height_range={3.4032854673293755E-8,0.077383084951129},height=3.309583223943099E-4]:7.1E-4,('03_Chlorella_fusca_var._vacuolata_UTEX252'[&length_range={7.140706990491558E-9,1.3107744428371784},height_95%_HPD={0.0,2.7755575615628914E-17},length_95%_HPD={7.503348235364601E-9,0.001536168306176904},rate=1.0,length=5.431596670804652E-4,length_median=3.48831007205678E-4,height_median=1.3877787807814457E-17,height_range={0.0,1.7763568394002505E-15},height=1.2336937128829786E-17]:2.26E-4,02_Chlorella_emersonii_CCAP_211_15_FR865661[&length_range={3.2455769773609643E-9,1.8690567640109153},height_95%_HPD={0.0,2.7755575615628914E-17},length_95%_HPD={3.2455769773609643E-9,0.0014909497849585813},rate=1.0,length=6.222237563546648E-4,length_median=3.3198505593557537E-4,height_median=1.3877787807814457E-17,height_range={0.0,1.7763568394002505E-15},height=1.2289956588851737E-17]:2.26E-4)[&length_range={3.147426588166703E-8,0.005427046400488169},height_95%_HPD={7.503348248283359E-9,9.88196124535129E-4},length_95%_HPD={3.147426588166703E-8,0.0014644269777667558},rate=1.0,length=4.909623933693199E-4,posterior=0.22143992959366343,length_median=3.398048213436411E-4,height_median=2.2634431513329778E-4,height_range={7.503348248283359E-9,0.004642563945575814},height=3.284154739510989E-4]:7.02E-4)[&length_range={3.254173354402508E-5,0.25259438086992014},height_95%_HPD={8.744831649973306E-5,0.0023333484650965297},length_95%_HPD={0.03160637763110998,0.06403612448065207},rate=1.0,length=0.04571440469790774,posterior=0.9996699702973267,length_median=0.046363017057996024,height_median=9.287171665438021E-4,height_range={3.749561098803644E-5,0.22766332611498435},height=0.0010782709268715824]:0.04422,'13_Coelastrella_sp._QW_2019b_FACHB_2314_MH176108'[&length_range={0.001083702114821473,1.7410263176079177},height_95%_HPD={0.0,2.7755575615628914E-17},length_95%_HPD={0.03462133162458395,0.05906142031053903},rate=1.0,length=0.04689007025971837,length_median=0.04672843555820923,height_median=1.3877787807814457E-17,height_range={0.0,1.7763568394002505E-15},height=1.2337006524014393E-17]:0.045148)[&length_range={7.777375519990581E-8,0.031925934537316826},height_95%_HPD={0.03447693680310164,0.056672631612344204},length_95%_HPD={7.777375519990581E-8,0.01511234263426564},rate=1.0,length=0.00716199707684337,posterior=0.5853126781410327,length_median=0.006666994382243864,height_median=0.045148483619271304,height_range={0.024787242227207247,0.22110836930574893},height=0.04529582306424959]:0.008497)[&length_range={0.00659172365736712,0.2730273773159604},height_95%_HPD={0.04397671125807938,0.06406173384863964},length_95%_HPD={0.015736528695807897,0.03970039277508286},rate=1.0,length=0.027474169622607,posterior=0.999909991899271,length_median=0.02714972825544075,height_median=0.05364574304289011,height_range={0.036395600548836345,0.7732522143065192},height=0.053921623470501986]:0.026345)[&length_range={2.1970430500589666E-6,0.35093055138338525},height_95%_HPD={0.06658705653175001,0.0948603398621156},length_95%_HPD={2.1970430500589666E-6,0.02015769679586865},rate=1.0,length=0.00994610419412518,posterior=0.7927413467212049,length_median=0.009367690355798444,height_median=0.07999055373017046,height_range={0.05428587108396395,1.0462795916224796},height=0.08035481782472124]:0.009352,(((28_Scenedesmus_obtusus_CCAP_276_36_AB037091[&length_range={3.700412897151076E-8,0.0660883121582829},height_95%_HPD={0.0,2.7755575615628914E-17},length_95%_HPD={3.808617875766704E-8,0.0015762802877416706},rate=1.0,length=5.284404612500924E-4,length_median=3.7364562337104747E-4,height_median=1.3877787807814457E-17,height_range={0.0,8.881784197001252E-16},height=1.2341447815829157E-17]:3.74E-4,'29_Scenedesmus_obtusus_SAG_52.80_X81966'[&length_range={3.700412897151076E-8,0.0660883121582829},height_95%_HPD={0.0,2.7755575615628914E-17},length_95%_HPD={3.808617875766704E-8,0.0015762802877416706},rate=1.0,length=5.284404612500924E-4,length_median=3.7364562337104747E-4,height_median=1.3877787807814457E-17,height_range={0.0,8.881784197001252E-16},height=1.2341447815829157E-17]:3.74E-4)[&length_range={0.023659588265898972,2.6080143501621973},height_95%_HPD={3.8086178766327805E-8,0.001576280287741677},length_95%_HPD={0.03305760666988711,0.05664535166657079},rate=1.0,length=0.044426190079414715,posterior=1.0,length_median=0.04387166129026811,height_median=3.736456233710689E-4,height_range={3.700412898322014E-8,0.06608831215828381},height=5.284404612501037E-4]:0.043948,33_Scotiellopsis_reticulata_CCALA_474_JX513885[&length_range={0.024990224028181164,5.817172978897288},height_95%_HPD={0.0,2.7755575615628914E-17},length_95%_HPD={0.033403137954722065,0.05706093532841101},rate=1.0,length=0.04518331675074823,length_median=0.04439643553373444,height_median=1.3877787807814457E-17,height_range={0.0,8.881784197001252E-16},height=1.2324931761893003E-17]:0.044321)[&length_range={1.2341611425270571E-5,0.3585668227529717},height_95%_HPD={0.03359567742988165,0.05682560223883152},length_95%_HPD={0.004097296378453789,0.02441330784584895},rate=1.0,length=0.01395609475102238,posterior=0.9851886669800282,length_median=0.01356492508172592,height_median=0.044321218384168234,height_range={0.024990224028181164,0.37999414630949624},height=0.04471082767513686]:0.013864,'01_Acutodesmus_obliquus_SAG_22.81_AB037096'[&length_range={0.030819181061546144,2.8829856611426132},height_95%_HPD={0.0,2.7755575615628914E-17},length_95%_HPD={0.044775225621096384,0.07292365595792434},rate=1.0,length=0.05866055159036609,length_median=0.05811306589694848,height_median=1.3877787807814457E-17,height_range={0.0,8.881784197001252E-16},height=1.2324099019677736E-17]:0.058185)[&length_range={0.0032618266967599924,0.34517990559351097},height_95%_HPD={0.045235647179418595,0.07300689403440999},length_95%_HPD={0.016602118328445672,0.04466745253526381},rate=1.0,length=0.03034073688287309,posterior=0.9998999909991899,length_median=0.030078817830047823,height_median=0.05818507470740523,height_range={0.03224031733017056,0.7385609690624679},height=0.058644198753843114]:0.031157)[&height_95%_HPD={0.07482135395734532,0.10513744579856078},length=0.0,posterior=1.0,height_median=0.08934245360468397,height_range={0.06220876238840358,7.374693389110325},height=0.09020433816375514];

end;

begin figtree;

set appearance.backgroundColorAttribute="Default";

set appearance.backgroundColour=#ffffff;

set appearance.branchColorAttribute="User selection";

set appearance.branchColorGradient=false;

set appearance.branchLineWidth=1.0;

set appearance.branchMinLineWidth=0.0;

set appearance.branchWidthAttribute="Fixed";

set appearance.foregroundColour=#000000;

set appearance.hilightingGradient=false;

set appearance.selectionColour=#2d3680;

set branchLabels.colorAttribute="User selection";

set branchLabels.displayAttribute="Branch times";

set branchLabels.fontName="sansserif";

set branchLabels.fontSize=8;

set branchLabels.fontStyle=0;

set branchLabels.isShown=false;

set branchLabels.significantDigits=4;

set layout.expansion=0;

set layout.layoutType="RECTILINEAR";

set layout.zoom=0;

set legend.attribute=null;

set legend.fontSize=10.0;

set legend.isShown=false;

set legend.significantDigits=4;

set nodeBars.barWidth=4.0;

set nodeBars.displayAttribute=null;

set nodeBars.isShown=false;

set nodeLabels.colorAttribute="User selection";

set nodeLabels.displayAttribute="posterior";

set nodeLabels.fontName="sansserif";

set nodeLabels.fontSize=11;

set nodeLabels.fontStyle=0;

set nodeLabels.isShown=true;

set nodeLabels.significantDigits=4;

set nodeShape.colourAttribute=null;

set nodeShape.isShown=false;

set nodeShape.minSize=10.0;

set nodeShape.scaleType=Width;

set nodeShape.shapeType=Circle;

set nodeShape.size=4.0;

set nodeShape.sizeAttribute=null;

set polarLayout.alignTipLabels=false;

set polarLayout.angularRange=0;

set polarLayout.rootAngle=0;

set polarLayout.rootLength=100;

set polarLayout.showRoot=true;

set radialLayout.spread=0.0;

set rectilinearLayout.alignTipLabels=false;

set rectilinearLayout.curvature=0;

set rectilinearLayout.rootLength=10000;

set scale.offsetAge=0.0;

set scale.rootAge=1.0;

set scale.scaleFactor=1.0;

set scale.scaleRoot=false;

set scaleAxis.automaticScale=true;

set scaleAxis.fontSize=8.0;

set scaleAxis.isShown=false;

set scaleAxis.lineWidth=1.0;

set scaleAxis.majorTicks=1.0;

set scaleAxis.origin=0.0;

set scaleAxis.reverseAxis=false;

set scaleAxis.showGrid=true;

set scaleBar.automaticScale=true;

set scaleBar.fontSize=10.0;

set scaleBar.isShown=true;

set scaleBar.lineWidth=1.0;

set scaleBar.scaleRange=0.0;

set tipLabels.colorAttribute="User selection";

set tipLabels.displayAttribute="Names";

set tipLabels.fontName="sansserif";

set tipLabels.fontSize=11;

set tipLabels.fontStyle=0;

set tipLabels.isShown=true;

set tipLabels.significantDigits=4;

set trees.order=true;

set trees.orderType="increasing";

set trees.rooting=true;

set trees.rootingType="User Selection";

set trees.transform=false;

set trees.transformType="cladogram";

end;
