# Supplementary material for: Lipid accumulation by Coelastrella multistriata (Scenedesmaceae, Sphaeropleales) during nitrogen and phosphorus starvation
Source: Sci Rep. 2021 Oct 6;11:19818. doi: 10.1038/s41598-021-99376-9 (PMC8494790; doi:10.1038/s41598-021-99376-9)
Supplement: Supplementary file 4 — Supplementary Information 4. [file 41598_2021_99376_MOESM4_ESM.docx]

#

1

CUCAGCGUCGGUUUACACCCUCACCCCUCCCAACUUGUUGGGCGUGUUUG

CUCUGUUUGCAAGCCAUUGGGGUGGAUCUGGCUUCCCCAAUCAUGUCCGU

UUCACUGCGGCAGACUGGGCUGGCUGAAGUGCAGAGGCUUAAGCAAGGAC

CCGAUAUGGGCUUCAACUGGAUAGGUAGCACCGGCUCUUGCCGACUACAC

GAAGUUGUUGCUUGUGGAUCUUGCUAGGAGCCAAGCAGGAACGUGCCUUU

GGCAUGUCUAACUUUCGACCUGAG

.((((.(((((.........(((((((.(((((....))))).(((((((

(.......))))).))).)))))))...(((((..((((.((.((.((((

......)))))))).))))..))))).........(((((.(((((((.(

(....((((((..(((((......((((...((((....)))).))))..

..)))))..)))))))).)))))))..))))).....(((.((((((...

))))))))).....))))))))).
