# Supplementary material for: Lipid accumulation by Coelastrella multistriata (Scenedesmaceae, Sphaeropleales) during nitrogen and phosphorus starvation
Source: Sci Rep. 2021 Oct 6;11:19818. doi: 10.1038/s41598-021-99376-9 (PMC8494790; doi:10.1038/s41598-021-99376-9)
Supplement: Supplementary file 5 — Supplementary Information 5. [file 41598_2021_99376_MOESM5_ESM.pdf]

ANALYSIS OF VARIANCE TABLES

14:0 Myristic acid (% of total fatty acids)

One-way ANOVA of kk=5 independent treatments

source	sum of squares SS	degrees of freedom νν	mean square MS	F statistic	p-value
treatment	3.2986	4	0.8246	515.4000	1.5663e-11
error	0.0160	10	0.0016		
total	3.3146	14			

Tukey HSD results

treatments pair	Tukey HSD Q statistic	Tukey HSD p-value	Tukey HSD inferfence
60 days culture vs control	41.1362	0.0010053	** p<0.01
60 days culture vs -N	52.3945	0.0010053	** p<0.01
60 days culture vs -P	52.3945	0.0010053	** p<0.01
60 days culture vs -N-P	52.3945	0.0010053	** p<0.01
control vs -N	11.2583	0.0010053	** p<0.01
control vs -P	11.2583	0.0010053	** p<0.01
control vs -N-P	11.2583	0.0010053	** p<0.01
-N vs -P	0.0000	0.8999947	insignificant
-N vs -N-P	0.0000	0.8999947	insignificant
-P vs -N-P	0.0000	0.8999947	insignificant

16:0 Palmitic acid (% of total fatty acids)

One-way ANOVA of kk=5 independent treatments

source	sum of squares SS	degrees of freedom νν	mean square MS	F statistic	p-value
treatment	62.0996	4	15.5249	2.1614	0.1472
error	71.8272	10	7.1827		

source	sum of squares SS	degrees of freedom νν	mean square MS	F statistic	p-value
total	133.9268	14			

Tukey HSD results

treatments pair	Tukey HSD Q statistic	Tukey HSD p-value	Tukey HSD inferfence
60 days culture vs control	1.1439	0.8999947	insignificant
60 days culture vs -N	1.6221	0.7598267	insignificant
60 days culture vs -P	2.0939	0.5884216	insignificant
60 days culture vs -N-P	0.9048	0.8999947	insignificant
control vs -N	0.4782	0.8999947	insignificant
control vs -P	3.2378	0.2250286	insignificant
control vs -N-P	0.2391	0.8999947	insignificant
-N vs -P	3.7161	0.1379058	insignificant
-N vs -N-P	0.7174	0.8999947	insignificant
-P vs -N-P	2.9987	0.2835330	insignificant

16:1n-7 cis-9-Palmitoleic acid (% of total fatty acids)

One-way ANOVA of kk=5 independent treatments

source	sum of squares SS	degrees of freedom νν	mean square MS	F statistic	p-value
treatment	12.7161	4	3.1790	18.7789	0.0001
error	1.6929	10	0.1693		
total	14.4089	14			

Tukey HSD results

treatments pair	Tukey HSD Q statistic	Tukey HSD p-value	Tukey HSD inferfence
60 days culture vs control	3.1011	0.2570837	insignificant

treatments pair	Tukey HSD Q statistic	Tukey HSD p-value	Tukey HSD inferfence
60 days culture vs -N	9.9629	0.0010053	** p<0.01
60 days culture vs -P	0.8560	0.8999947	insignificant
60 days culture vs -N-P	4.9113	0.0377450	* p<0.05
control vs -N	6.8618	0.0047031	** p<0.01
control vs -P	3.9571	0.1068738	insignificant
control vs -N-P	1.8102	0.6915149	insignificant
-N vs -P	10.8189	0.0010053	** p<0.01
-N vs -N-P	5.0516	0.0323582	* p<0.05
-P vs -N-P	5.7673	0.0148502	* p<0.05

16:2n-6 cis-7,10-Hexadecadienoic acid (% of total fatty acids)

One-way ANOVA of kk=5 independent treatments

source	sum of squares SS	degrees of freedom νν	mean square MS	F statistic	p-value
treatment	19.1137	4	4.7784	28.8332	1.8111e-05
error	1.6573	10	0.1657		
total	20.7710	14			

Tukey HSD results

treatments pair	Tukey HSD Q statistic	Tukey HSD p-value	Tukey HSD inferfence
60 days culture vs control	8.9631	0.0010053	** p<0.01
60 days culture vs -N	3.1768	0.2388148	insignificant
60 days culture vs -P	7.3464	0.0028870	** p<0.01
60 days culture vs -N-P	0.9077	0.8999947	insignificant
control vs -N	12.1400	0.0010053	** p<0.01

treatments pair	Tukey HSD Q statistic	Tukey HSD p-value	Tukey HSD inferfence
control vs -P	1.6168	0.7617798	insignificant
control vs -N-P	9.8708	0.0010053	** p<0.01
-N vs -P	10.5232	0.0010053	** p<0.01
-N vs -N-P	2.2691	0.5247579	insignificant
-P vs -N-P	8.2540	0.0012022	** p<0.01

16:3n-3 cis-7,10,13-Hexadecatrienoic acid (% of total fatty acids)

One-way ANOVA of kk=5 independent treatments

source	sum of squares SS	degrees of freedom νν	mean square MS	F statistic	p-value
treatment	407.0275	4	101.7569	23.4637	4.5676e-05
error	43.3677	10	4.3368		
total	450.3952	14			

Tukey HSD results

treatments pair	Tukey HSD Q statistic	Tukey HSD p-value	Tukey HSD inferfence
60 days culture vs control	8.8606	0.0010053	** p<0.01
60 days culture vs -N	3.3463	0.2019235	insignificant
60 days culture vs -P	3.2298	0.2268108	insignificant
60 days culture vs -N-P	1.8187	0.6884167	insignificant
control vs -N	12.2069	0.0010053	** p<0.01
control vs -P	5.6307	0.0172095	* p<0.05
control vs -N-P	10.6793	0.0010053	** p<0.01
-N vs -P	6.5761	0.0063081	** p<0.01
-N vs -N-P	1.5276	0.7941784	insignificant

treatments pair	Tukey HSD Q statistic	Tukey HSD p-value	Tukey HSD inferfence
-P vs -N-P	5.0485	0.0324702	* p<0.05

18:0 Stearic acid (% of total fatty acids)

One-way ANOVA of kk=5 independent treatments

source	sum of squares SS	degrees of freedom νν	mean square MS	F statistic	p-value
treatment	26.4464	4	6.6116	15.6355	0.0003
error	4.2286	10	0.4229		
total	30.6750	14			

Tukey HSD results

treatments pair	Tukey HSD Q statistic	Tukey HSD p-value	Tukey HSD inferfence
60 days culture vs control	3.7023	0.1399199	insignificant
60 days culture vs -N	6.0996	0.0104099	* p<0.05
60 days culture vs -P	2.4238	0.4688524	insignificant
60 days culture vs -N-P	5.0075	0.0339628	* p<0.05
control vs -N	9.8019	0.0010053	** p<0.01
control vs -P	6.1262	0.0101190	* p<0.05
control vs -N-P	8.7098	0.0010053	** p<0.01
-N vs -P	3.6757	0.1439109	insignificant
-N vs -N-P	1.0921	0.8999947	insignificant
-P vs -N-P	2.5837	0.4121009	insignificant

18:1n-9 cis-9-Oleic acid (% of total fatty acids)

One-way ANOVA of kk=5 independent treatments

source	sum of squares SS	degrees of freedom νν	mean square MS	F statistic	p-value
treatment	2,410.1475	4	602.5369	170.2960	3.7576e-09
error	35.3817	10	3.5382		
total	2,445.5292	14			

Tukey HSD results

treatments pair	Tukey HSD Q statistic	Tukey HSD p-value	Tukey HSD inferfence
60 days culture vs control	4.2726	0.0760289	insignificant
60 days culture vs -N	27.1793	0.0010053	** p<0.01
60 days culture vs -P	9.0976	0.0010053	** p<0.01
60 days culture vs -N-P	27.9037	0.0010053	** p<0.01
control vs -N	22.9067	0.0010053	** p<0.01
control vs -P	4.8251	0.0414945	* p<0.05
control vs -N-P	23.6311	0.0010053	** p<0.01
-N vs -P	18.0817	0.0010053	** p<0.01
-N vs -N-P	0.7244	0.8999947	insignificant
-P vs -N-P	18.8061	0.0010053	** p<0.01

18:1n-7 cis-11-Vaccenic acid (% of total fatty acids)

One-way ANOVA of kk=5 independent treatments

source	sum of squares SS	degrees of freedom νν	mean square MS	F statistic	p-value
treatment	23.9254	4	5.9813	33.9810	8.5502e-06
error	1.7602	10	0.1760		
total	25.6856	14			

Tukey HSD results

treatments pair	Tukey HSD Q statistic	Tukey HSD p-value	Tukey HSD inferfence
60 days culture vs control	2.3119	0.5092298	insignificant
60 days culture vs -N	12.0549	0.0010053	** p<0.01
60 days culture vs -P	2.3532	0.4942919	insignificant
60 days culture vs -N-P	12.0549	0.0010053	** p<0.01
control vs -N	9.7430	0.0010053	** p<0.01
control vs -P	0.0413	0.8999947	insignificant
control vs -N-P	9.7430	0.0010053	** p<0.01
-N vs -P	9.7017	0.0010053	** p<0.01
-N vs -N-P	0.0000	0.8999947	insignificant
-P vs -N-P	9.7017	0.0010053	** p<0.01

18:2n-6 cis-9,12-Linoleic acid (% of total fatty acids)

One-way ANOVA of kk=5 independent treatments

source	sum of squares SS	degrees of freedom νν	mean square MS	F statistic	p-value
treatment	39.8458	4	9.9615	2.3052	0.1296
error	43.2129	10	4.3213		
total	83.0587	14			

Tukey HSD results

treatments pair	Tukey HSD Q statistic	Tukey HSD p-value	Tukey HSD inferfence
60 days culture vs control	1.5581	0.7830946	insignificant
60 days culture vs -N	2.2885	0.5177123	insignificant
60 days culture vs -P	0.9082	0.8999947	insignificant
60 days culture vs -N-P	0.9165	0.8999947	insignificant

treatments pair	Tukey HSD Q statistic	Tukey HSD p-value	Tukey HSD inferfence
control vs -N	3.8466	0.1203050	insignificant
control vs -P	0.6499	0.8999947	insignificant
control vs -N-P	2.4746	0.4506269	insignificant
-N vs -P	3.1967	0.2343062	insignificant
-N vs -N-P	1.3720	0.8507008	insignificant
-P vs -N-P	1.8247	0.6862243	insignificant

18:3n-3 cis-9,12,15-α-Linolenic acid (% of total fatty acids)

One-way ANOVA of kk=5 independent treatments

source	sum of squares SS	degrees of freedom νν	mean square MS	F statistic	p-value
treatment	1,024.5631	4	256.1408	110.6124	3.1062e-08
error	23.1566	10	2.3157		
total	1,047.7197	14			

Tukey HSD results

treatments pair	Tukey HSD Q statistic	Tukey HSD p-value	Tukey HSD inferfence
60 days culture vs control	5.1713	0.0283825	* p<0.05
60 days culture vs -N	18.2380	0.0010053	** p<0.01
60 days culture vs -P	4.7805	0.0435750	* p<0.05
60 days culture vs -N-P	17.6840	0.0010053	** p<0.01
control vs -N	23.4092	0.0010053	** p<0.01
control vs -P	9.9518	0.0010053	** p<0.01
control vs -N-P	22.8553	0.0010053	** p<0.01
-N vs -P	13.4575	0.0010053	** p<0.01

treatments pair	Tukey HSD Q statistic	Tukey HSD p-value	Tukey HSD inferfence
-N vs -N-P	0.5539	0.8999947	insignificant
-P vs -N-P	12.9035	0.0010053	** p<0.01

20:0 Arachidic acid (% of total fatty acids)

One-way ANOVA of kk=5 independent treatments

source	sum of squares SS	degrees of freedom νν	mean square MS	F statistic	p-value
treatment	4.9308	4	1.2327	56.2872	8.0495e-07
error	0.2190	10	0.0219		
total	5.1498	14			

Tukey HSD results

treatments pair	Tukey HSD Q statistic	Tukey HSD p-value	Tukey HSD inferfence
60 days culture vs control	5.5790	0.0182008	* p<0.05
60 days culture vs -N	11.2360	0.0010053	** p<0.01
60 days culture vs -P	5.5790	0.0182008	* p<0.05
60 days culture vs -N-P	6.7884	0.0050674	** p<0.01
control vs -N	16.8149	0.0010053	** p<0.01
control vs -P	0.0000	0.8999947	insignificant
control vs -N-P	12.3673	0.0010053	** p<0.01
-N vs -P	16.8149	0.0010053	** p<0.01
-N vs -N-P	4.4476	0.0627981	insignificant
-P vs -N-P	12.3673	0.0010053	** p<0.01

22:0 Behenic acid (% of total fatty acids)

One-way ANOVA of kk=5 independent treatments

source	sum of squares SS	degrees of freedom νν	mean square MS	F statistic	p-value
treatment	7.2311	4	1.8078	276.4179	3.4452e-10
error	0.0654	10	0.0065		
total	7.2965	14			

Tukey HSD results

treatments pair	Tukey HSD Q statistic	Tukey HSD p-value	Tukey HSD inferfence
60 days culture vs control	16.1346	0.0010053	** p<0.01
60 days culture vs -N	20.8465	0.0010053	** p<0.01
60 days culture vs -P	21.9888	0.0010053	** p<0.01
60 days culture vs -N-P	5.8542	0.0135251	* p<0.05
control vs -N	36.9811	0.0010053	** p<0.01
control vs -P	5.8542	0.0135251	* p<0.05
control vs -N-P	10.2805	0.0010053	** p<0.01
-N vs -P	42.8353	0.0010053	** p<0.01
-N vs -N-P	26.7007	0.0010053	** p<0.01
-P vs -N-P	16.1346	0.0010053	** p<0.01

24:0 Lignoceric acid (% of total fatty acids)

One-way ANOVA of kk=5 independent treatments

source	sum of squares SS	degrees of freedom νν	mean square MS	F statistic	p-value
treatment	23.6997	4	5.9249	189.1741	2.2402e-09
error	0.3132	10	0.0313		
total	24.0129	14			

Tukey HSD results

treatments pair	Tukey HSD Q statistic	Tukey HSD p-value	Tukey HSD inferfence
60 days culture vs control	35.8857	0.0010053	** p<0.01
60 days culture vs -N	21.3357	0.0010053	** p<0.01
60 days culture vs -P	26.7511	0.0010053	** p<0.01
60 days culture vs -N-P	29.7199	0.0010053	** p<0.01
control vs -N	14.5500	0.0010053	** p<0.01
control vs -P	9.1345	0.0010053	** p<0.01
control vs -N-P	6.1658	0.0097007	** p<0.01
-N vs -P	5.4155	0.0217377	* p<0.05
-N vs -N-P	8.3842	0.0010655	** p<0.01
-P vs -N-P	2.9687	0.2916388	insignificant

26:0 Cerotic acid (% of total fatty acids)

One-way ANOVA of kk=5 independent treatments

source	sum of squares SS	degrees of freedom νν	mean square MS	F statistic	p-value
treatment	33.9510	4	8.4878	802.7516	1.7261e-12
error	0.1057	10	0.0106		
total	34.0568	14			

Tukey HSD results

treatments pair	Tukey HSD Q statistic	Tukey HSD p-value	Tukey HSD inferfence
60 days culture vs control	65.2438	0.0010053	** p<0.01
60 days culture vs -N	65.2438	0.0010053	** p<0.01
60 days culture vs -P	44.1322	0.0010053	** p<0.01
60 days culture vs -N-P	65.2438	0.0010053	** p<0.01

treatments pair	Tukey HSD Q statistic	Tukey HSD p-value	Tukey HSD inferfence
control vs -N	0.0000	0.8999947	insignificant
control vs -P	21.1116	0.0010053	** p<0.01
control vs -N-P	0.0000	0.8999947	insignificant
-N vs -P	21.1116	0.0010053	** p<0.01
-N vs -N-P	0.0000	0.8999947	insignificant
-P vs -N-P	21.1116	0.0010053	** p<0.01

ΣSFAs (% of total fatty acids)

One-way ANOVA of kk=5 independent treatments

source	sum of squares SS	degrees of freedom νν	mean square MS	F statistic	p-value
treatment	282.0929	4	70.5232	5.0820	0.0170
error	138.7697	10	13.8770		
total	420.8626	14			

Tukey HSD results

treatments pair	Tukey HSD Q statistic	Tukey HSD p-value	Tukey HSD inferfence
60 days culture vs control	6.1746	0.0096113	** p<0.01
60 days culture vs -N	3.0191	0.2781043	insignificant
60 days culture vs -P	2.2597	0.5281951	insignificant
60 days culture vs -N-P	3.8483	0.1200941	insignificant
control vs -N	3.1555	0.2438460	insignificant
control vs -P	3.9149	0.1118300	insignificant
control vs -N-P	2.3263	0.5039813	insignificant
-N vs -P	0.7594	0.8999947	insignificant

treatments pair	Tukey HSD Q statistic	Tukey HSD p-value	Tukey HSD inferfence
-N vs -N-P	0.8292	0.8999947	insignificant
-P vs -N-P	1.5886	0.7720101	insignificant

ΣMUFAs (% of total fatty acids)

One-way ANOVA of kk=5 independent treatments

source	sum of squares SS	degrees of freedom νν	mean square MS	F statistic	p-value
treatment	1,666.6840	4	416.6710	72.5314	2.3956e-07
error	57.4470	10	5.7447		
total	1,724.1310	14			

Tukey HSD results

treatments pair	Tukey HSD Q statistic	Tukey HSD p-value	Tukey HSD inferfence
60 days culture vs control	1.6380	0.7540641	insignificant
60 days culture vs -N	16.9606	0.0010053	** p<0.01
60 days culture vs -P	6.0967	0.0104408	* p<0.05
60 days culture vs -N-P	18.1674	0.0010053	** p<0.01
control vs -N	15.3226	0.0010053	** p<0.01
control vs -P	4.4587	0.0620338	insignificant
control vs -N-P	16.5294	0.0010053	** p<0.01
-N vs -P	10.8638	0.0010053	** p<0.01
-N vs -N-P	1.2068	0.8999947	insignificant
-P vs -N-P	12.0706	0.0010053	** p<0.01

ΣSFAs+MUFAs (% of total fatty acids)

One-way ANOVA of kk=5 independent treatments

source	sum of squares SS	degrees of freedom νν	mean square MS	F statistic	p-value
treatment	1,701.7142	4	425.4286	11.5112	0.0009
error	369.5779	10	36.9578		
total	2,071.2921	14			

Tukey HSD results

treatments pair	Tukey HSD Q statistic	Tukey HSD p-value	Tukey HSD inferfence
60 days culture vs control	3.1378	0.2480926	insignificant
60 days culture vs -N	4.8368	0.0409636	* p<0.05
60 days culture vs -P	1.0190	0.8999947	insignificant
60 days culture vs -N-P	4.8045	0.0424386	* p<0.05
control vs -N	7.9746	0.0015649	** p<0.01
control vs -P	4.1568	0.0862028	insignificant
control vs -N-P	7.9423	0.0016149	** p<0.01
-N vs -P	3.8178	0.1240578	insignificant
-N vs -N-P	0.0323	0.8999947	insignificant
-P vs -N-P	3.7855	0.1281280	insignificant

ΣPUFAs (% of total fatty acids)

One-way ANOVA of kk=5 independent treatments

source	sum of squares SS	degrees of freedom νν	mean square MS	F statistic	p-value
treatment	1,701.7142	4	425.4286	11.5112	0.0009
error	369.5779	10	36.9578		
total	2,071.2921	14			

Tukey HSD results

treatments pair	Tukey HSD Q statistic	Tukey HSD p-value	Tukey HSD inferfence
60 days culture vs control	3.1378	0.2480926	insignificant
60 days culture vs -N	4.8368	0.0409636	* p<0.05
60 days culture vs -P	1.0190	0.8999947	insignificant
60 days culture vs -N-P	4.8045	0.0424386	* p<0.05
control vs -N	7.9746	0.0015649	** p<0.01
control vs -P	4.1568	0.0862028	insignificant
control vs -N-P	7.9423	0.0016149	** p<0.01
-N vs -P	3.8178	0.1240578	insignificant
-N vs -N-P	0.0323	0.8999947	insignificant
-P vs -N-P	3.7855	0.1281280	insignificant

14:0 Myristic acid (mg L^{-1})

One-way ANOVA of kk=5 independent treatments

source	sum of squares SS	degrees of freedom νν	mean square MS	F statistic	p-value
treatment	0.1070	4	0.0268	308.7308	1.9931e-10
error	0.0009	10	0.0001		
total	0.1079	14			

Tukey HSD results

treatments pair	Tukey HSD Q statistic	Tukey HSD p-value	Tukey HSD inferfence
60 days culture vs control	34.7297	0.0010053	** p<0.01
60 days culture vs -N	40.3113	0.0010053	** p<0.01
60 days culture vs -P	40.3113	0.0010053	** p<0.01
60 days culture vs -N-P	40.3113	0.0010053	** p<0.01

treatments pair	Tukey HSD Q statistic	Tukey HSD p-value	Tukey HSD inferfence
control vs -N	5.5816	0.0181472	* p<0.05
control vs -P	5.5816	0.0181472	* p<0.05
control vs -N-P	5.5816	0.0181472	* p<0.05
-N vs -P	0.0000	0.8999947	insignificant
-N vs -N-P	0.0000	0.8999947	insignificant
-P vs -N-P	0.0000	0.8999947	insignificant

16:0 Palmitic acid (mg L^{-1})

One-way ANOVA of kk=5 independent treatments

source	sum of squares SS	degrees of freedom νν	mean square MS	F statistic	p-value
treatment	6.7523	4	1.6881	8.3376	0.0032
error	2.0247	10	0.2025		
total	8.7770	14			

Tukey HSD results

treatments pair	Tukey HSD Q statistic	Tukey HSD p-value	Tukey HSD inferfence
60 days culture vs control	5.6457	0.0169315	* p<0.05
60 days culture vs -N	2.1428	0.5706696	insignificant
60 days culture vs -P	3.1436	0.2466939	insignificant
60 days culture vs -N-P	1.8605	0.6732285	insignificant
control vs -N	3.5029	0.1723216	insignificant
control vs -P	2.5021	0.4408387	insignificant
control vs -N-P	7.5062	0.0024646	** p<0.01
-N vs -P	1.0008	0.8999947	insignificant

treatments pair	Tukey HSD Q statistic	Tukey HSD p-value	Tukey HSD inferfence
-N vs -N-P	4.0033	0.1016784	insignificant
-P vs -N-P	5.0041	0.0340890	* p<0.05

16:1n-7 cis-9-Palmitoleic acid (mg L^{-1})

One-way ANOVA of kk=5 independent treatments

source	sum of squares SS	degrees of freedom νν	mean square MS	F statistic	p-value
treatment	0.2979	4	0.0745	20.0926	9.0365e-05
error	0.0371	10	0.0037		
total	0.3350	14			

Tukey HSD results

treatments pair	Tukey HSD Q statistic	Tukey HSD p-value	Tukey HSD inferfence
60 days culture vs control	6.5433	0.0065269	** p<0.01
60 days culture vs -N	12.0435	0.0010053	** p<0.01
60 days culture vs -P	3.1294	0.2501294	insignificant
60 days culture vs -N-P	4.5519	0.0560114	insignificant
control vs -N	5.5002	0.0198231	* p<0.05
control vs -P	3.4139	0.1885523	insignificant
control vs -N-P	1.9914	0.6256559	insignificant
-N vs -P	8.9141	0.0010053	** p<0.01
-N vs -N-P	7.4916	0.0024999	** p<0.01
-P vs -N-P	1.4225	0.8323769	insignificant

16:2n-6 cis-7,10-Hexadecadienoic acid (mg L^{-1})

One-way ANOVA of kk=5 independent treatments

source	sum of squares SS	degrees of freedom νν	mean square MS	F statistic	p-value
treatment	1.1024	4	0.2756	16.3341	0.0002
error	0.1687	10	0.0169		
total	1.2712	14			

Tukey HSD results

treatments pair	Tukey HSD Q statistic	Tukey HSD p-value	Tukey HSD inferfence
60 days culture vs control	6.2225	0.0091370	** p<0.01
60 days culture vs -N	1.4667	0.8162861	insignificant
60 days culture vs -P	5.5558	0.0186609	* p<0.05
60 days culture vs -N-P	2.4446	0.4614038	insignificant
control vs -N	7.6893	0.0020630	** p<0.01
control vs -P	0.6667	0.8999947	insignificant
control vs -N-P	8.6671	0.0010053	** p<0.01
-N vs -P	7.0226	0.0039922	** p<0.01
-N vs -N-P	0.9778	0.8999947	insignificant
-P vs -N-P	8.0004	0.0015277	** p<0.01

16:3n-3 cis-7,10,13-Hexadecatrienoic acid (mg L^{-1})

One-way ANOVA of kk=5 independent treatments

source	sum of squares SS	degrees of freedom νν	mean square MS	F statistic	p-value
treatment	3.1385	4	0.7846	16.9832	0.0002
error	0.4620	10	0.0462		
total	3.6005	14			

Tukey HSD results

treatments pair	Tukey HSD Q statistic	Tukey HSD p-value	Tukey HSD inferfence
60 days culture vs control	5.5870	0.0180422	* p<0.05
60 days culture vs -N	6.0168	0.0113667	* p<0.05
60 days culture vs -P	0.5641	0.8999947	insignificant
60 days culture vs -N-P	0.9938	0.8999947	insignificant
control vs -N	11.6039	0.0010053	** p<0.01
control vs -P	6.1511	0.0098539	** p<0.01
control vs -N-P	6.5809	0.0062769	** p<0.01
-N vs -P	5.4527	0.0208741	* p<0.05
-N vs -N-P	5.0230	0.0333950	* p<0.05
-P vs -N-P	0.4298	0.8999947	insignificant

18:0 Stearic acid (mg L^{-1})

One-way ANOVA of kk=5 independent treatments

source	sum of squares SS	degrees of freedom νν	mean square MS	F statistic	p-value
treatment	1.4433	4	0.3608	27.5573	2.2228e-05
error	0.1309	10	0.0131		
total	1.5742	14			

Tukey HSD results

treatments pair	Tukey HSD Q statistic	Tukey HSD p-value	Tukey HSD inferfence
60 days culture vs control	5.1465	0.0291638	* p<0.05
60 days culture vs -N	5.7015	0.0159404	* p<0.05
60 days culture vs -P	0.8578	0.8999947	insignificant
60 days culture vs -N-P	7.8712	0.0017303	** p<0.01

treatments pair	Tukey HSD Q statistic	Tukey HSD p-value	Tukey HSD inferfence
control vs -N	10.8481	0.0010053	** p<0.01
control vs -P	4.2888	0.0746983	insignificant
control vs -N-P	13.0177	0.0010053	** p<0.01
-N vs -P	6.5593	0.0064202	** p<0.01
-N vs -N-P	2.1696	0.5609228	insignificant
-P vs -N-P	8.7289	0.0010053	** p<0.01

18:1n-9 cis-9-Oleic acid (mg L^{-1})

One-way ANOVA of kk=5 independent treatments

source	sum of squares SS	degrees of freedom νν	mean square MS	F statistic	p-value
treatment	113.6487	4	28.4122	36.4645	6.1751e-06
error	7.7917	10	0.7792		
total	121.4404	14			

Tukey HSD results

treatments pair	Tukey HSD Q statistic	Tukey HSD p-value	Tukey HSD inferfence
60 days culture vs control	0.6410	0.8999947	insignificant
60 days culture vs -N	10.0007	0.0010053	** p<0.01
60 days culture vs -P	1.9295	0.6481616	insignificant
60 days culture vs -N-P	13.2187	0.0010053	** p<0.01
control vs -N	9.3597	0.0010053	** p<0.01
control vs -P	1.2885	0.8810403	insignificant
control vs -N-P	12.5777	0.0010053	** p<0.01
-N vs -P	8.0712	0.0014270	** p<0.01

treatments pair	Tukey HSD Q statistic	Tukey HSD p-value	Tukey HSD inferfence
-N vs -N-P	3.2180	0.2294701	insignificant
-P vs -N-P	11.2892	0.0010053	** p<0.01

18:1n-7 cis-11-Vaccenic acid (mg L^{-1})

One-way ANOVA of kk=5 independent treatments

source	sum of squares SS	degrees of freedom νν	mean square MS	F statistic	p-value
treatment	0.5892	4	0.1473	36.5182	6.1332e-06
error	0.0403	10	0.0040		
total	0.6295	14			

Tukey HSD results

treatments pair	Tukey HSD Q statistic	Tukey HSD p-value	Tukey HSD inferfence
60 days culture vs control	6.5455	0.0065125	** p<0.01
60 days culture vs -N	14.2727	0.0010053	** p<0.01
60 days culture vs -P	6.6364	0.0059262	** p<0.01
60 days culture vs -N-P	14.2727	0.0010053	** p<0.01
control vs -N	7.7273	0.0019881	** p<0.01
control vs -P	0.0909	0.8999947	insignificant
control vs -N-P	7.7273	0.0019881	** p<0.01
-N vs -P	7.6364	0.0021727	** p<0.01
-N vs -N-P	0.0000	0.8999947	insignificant
-P vs -N-P	7.6364	0.0021727	** p<0.01

18:2n-6 cis-9,12-Linoleic acid (mg L^{-1})

One-way ANOVA of kk=5 independent treatments

source	sum of squares SS	degrees of freedom νν	mean square MS	F statistic	p-value
treatment	4.7715	4	1.1929	16.0088	0.0002
error	0.7451	10	0.0745		
total	5.5166	14			

Tukey HSD results

treatments pair	Tukey HSD Q statistic	Tukey HSD p-value	Tukey HSD inferfence
60 days culture vs control	5.0338	0.0329954	* p<0.05
60 days culture vs -N	2.6861	0.3771903	insignificant
60 days culture vs -P	4.3147	0.0726150	insignificant
60 days culture vs -N-P	3.8071	0.1252242	insignificant
control vs -N	7.7200	0.0020006	** p<0.01
control vs -P	0.7191	0.8999947	insignificant
control vs -N-P	8.8409	0.0010053	** p<0.01
-N vs -P	7.0008	0.0040819	** p<0.01
-N vs -N-P	1.1210	0.8999947	insignificant
-P vs -N-P	8.1218	0.0013631	** p<0.01

18:3n-3 cis-9,12,15-α-Linolenic acid (mg L^{-1})

One-way ANOVA of kk=5 independent treatments

source	sum of squares SS	degrees of freedom νν	mean square MS	F statistic	p-value
treatment	15.7685	4	3.9421	27.5097	2.2403e-05
error	1.4330	10	0.1433		
total	17.2015	14			

Tukey HSD results

treatments pair	Tukey HSD Q statistic	Tukey HSD p-value	Tukey HSD inferfence
60 days culture vs control	7.0768	0.0037822	** p<0.01
60 days culture vs -N	13.6045	0.0010053	** p<0.01
60 days culture vs -P	11.4387	0.0010053	** p<0.01
60 days culture vs -N-P	9.5933	0.0010053	** p<0.01
control vs -N	6.5277	0.0066324	** p<0.01
control vs -P	4.3620	0.0689640	insignificant
control vs -N-P	2.5165	0.4356989	insignificant
-N vs -P	2.1657	0.5623313	insignificant
-N vs -N-P	4.0112	0.1008170	insignificant
-P vs -N-P	1.8454	0.6786976	insignificant

20:0 Arachidic acid (mg L^{-1})

One-way ANOVA of kk=5 independent treatments

source	sum of squares SS	degrees of freedom νν	mean square MS	F statistic	p-value
treatment	0.1757	4	0.0439	56.3248	8.0240e-07
error	0.0078	10	0.0008		
total	0.1835	14			

Tukey HSD results

treatments pair	Tukey HSD Q statistic	Tukey HSD p-value	Tukey HSD inferfence
60 days culture vs control	5.3748	0.0227220	* p<0.05
60 days culture vs -N	9.9228	0.0010053	** p<0.01
60 days culture vs -P	5.3748	0.0227220	* p<0.05
60 days culture vs -N-P	9.0959	0.0010053	** p<0.01

treatments pair	Tukey HSD Q statistic	Tukey HSD p-value	Tukey HSD inferfence
control vs -N	15.2976	0.0010053	** p<0.01
control vs -P	0.0000	0.8999947	insignificant
control vs -N-P	14.4707	0.0010053	** p<0.01
-N vs -P	15.2976	0.0010053	** p<0.01
-N vs -N-P	0.8269	0.8999947	insignificant
-P vs -N-P	14.4707	0.0010053	** p<0.01

22:0 Behenic acid (mg L^{-1})

One-way ANOVA of kk=5 independent treatments

source	sum of squares SS	degrees of freedom νν	mean square MS	F statistic	p-value
treatment	0.2362	4	0.0591	66.1082	3.7375e-07
error	0.0089	10	0.0009		
total	0.2452	14			

Tukey HSD results

treatments pair	Tukey HSD Q statistic	Tukey HSD p-value	Tukey HSD inferfence
60 days culture vs control	9.0788	0.0010053	** p<0.01
60 days culture vs -N	9.6583	0.0010053	** p<0.01
60 days culture vs -P	10.6242	0.0010053	** p<0.01
60 days culture vs -N-P	1.3522	0.8579141	insignificant
control vs -N	18.7372	0.0010053	** p<0.01
control vs -P	1.5453	0.7877314	insignificant
control vs -N-P	7.7267	0.0019890	** p<0.01
-N vs -P	20.2825	0.0010053	** p<0.01

treatments pair	Tukey HSD Q statistic	Tukey HSD p-value	Tukey HSD inferfence
-N vs -N-P	11.0105	0.0010053	** p<0.01
-P vs -N-P	9.2720	0.0010053	** p<0.01

24:0 Lignoceric acid (mg L^{-1})

One-way ANOVA of kk=5 independent treatments

source	sum of squares SS	degrees of freedom νν	mean square MS	F statistic	p-value
treatment	0.7790	4	0.1948	99.7065	5.1465e-08
error	0.0195	10	0.0020		
total	0.7986	14			

Tukey HSD results

treatments pair	Tukey HSD Q statistic	Tukey HSD p-value	Tukey HSD inferfence
60 days culture vs control	25.8652	0.0010053	** p<0.01
60 days culture vs -N	15.6759	0.0010053	** p<0.01
60 days culture vs -P	21.4237	0.0010053	** p<0.01
60 days culture vs -N-P	20.2480	0.0010053	** p<0.01
control vs -N	10.1893	0.0010053	** p<0.01
control vs -P	4.4415	0.0632176	insignificant
control vs -N-P	5.6172	0.0174613	* p<0.05
-N vs -P	5.7478	0.0151652	* p<0.05
-N vs -N-P	4.5721	0.0547787	insignificant
-P vs -N-P	1.1757	0.8999947	insignificant

26:0 Cerotic acid (mg L^{-1})

One-way ANOVA of kk=5 independent treatments

source	sum of squares SS	degrees of freedom νν	mean square MS	F statistic	p-value
treatment	1.0934	4	0.2734	261.1688	4.5616e-10
error	0.0105	10	0.0010		
total	1.1039	14			

Tukey HSD results

treatments pair	Tukey HSD Q statistic	Tukey HSD p-value	Tukey HSD inferfence
60 days culture vs control	37.2976	0.0010053	** p<0.01
60 days culture vs -N	37.2976	0.0010053	** p<0.01
60 days culture vs -P	29.2671	0.0010053	** p<0.01
60 days culture vs -N-P	37.2976	0.0010053	** p<0.01
control vs -N	0.0000	0.8999947	insignificant
control vs -P	8.0306	0.0014833	** p<0.01
control vs -N-P	0.0000	0.8999947	insignificant
-N vs -P	8.0306	0.0014833	** p<0.01
-N vs -N-P	0.0000	0.8999947	insignificant
-P vs -N-P	8.0306	0.0014833	** p<0.01

ΣSFAs (mg L^{-1})

One-way ANOVA of kk=5 independent treatments

source	sum of squares SS	degrees of freedom νν	mean square MS	F statistic	p-value
treatment	28.0752	4	7.0188	17.8826	0.0001
error	3.9249	10	0.3925		
total	32.0001	14			

Tukey HSD results

treatments pair	Tukey HSD Q statistic	Tukey HSD p-value	Tukey HSD inferfence
60 days culture vs control	10.2293	0.0010053	** p<0.01
60 days culture vs -N	3.6770	0.1437092	insignificant
60 days culture vs -P	7.2342	0.0032281	** p<0.01
60 days culture vs -N-P	1.2902	0.8804314	insignificant
control vs -N	6.5523	0.0064647	** p<0.01
control vs -P	2.9951	0.2845091	insignificant
control vs -N-P	8.9391	0.0010053	** p<0.01
-N vs -P	3.5572	0.1629115	insignificant
-N vs -N-P	2.3868	0.4821716	insignificant
-P vs -N-P	5.9441	0.0122861	* p<0.05

ΣMUFAs (mg L^{-1})

One-way ANOVA of kk=5 independent treatments

source	sum of squares SS	degrees of freedom νν	mean square MS	F statistic	p-value
treatment	92.6119	4	23.1530	26.8781	2.4876e-05
error	8.6141	10	0.8614		
total	101.2260	14			

Tukey HSD results

treatments pair	Tukey HSD Q statistic	Tukey HSD p-value	Tukey HSD inferfence
60 days culture vs control	0.6345	0.8999947	insignificant
60 days culture vs -N	7.4959	0.0024906	** p<0.01
60 days culture vs -P	0.8087	0.8999947	insignificant
60 days culture vs -N-P	10.9297	0.0010053	** p<0.01

treatments pair	Tukey HSD Q statistic	Tukey HSD p-value	Tukey HSD inferfence
control vs -N	8.1304	0.0013523	** p<0.01
control vs -P	1.4432	0.8248447	insignificant
control vs -N-P	11.5642	0.0010053	** p<0.01
-N vs -P	6.6872	0.0056245	** p<0.01
-N vs -N-P	3.4338	0.1847660	insignificant
-P vs -N-P	10.1210	0.0010053	** p<0.01

ΣSFAs+MUFAs (mg L^{-1})

One-way ANOVA of kk=5 independent treatments

source	sum of squares SS	degrees of freedom νν	mean square MS	F statistic	p-value
treatment	185.1176	4	46.2794	24.6336	3.6781e-05
error	18.7871	10	1.8787		
total	203.9048	14			

Tukey HSD results

treatments pair	Tukey HSD Q statistic	Tukey HSD p-value	Tukey HSD inferfence
60 days culture vs control	5.1010	0.0306548	* p<0.05
60 days culture vs -N	5.1010	0.0306548	* p<0.05
60 days culture vs -P	2.7548	0.3547777	insignificant
60 days culture vs -N-P	6.8153	0.0049317	** p<0.01
control vs -N	0.0000	0.8999947	insignificant
control vs -P	2.3462	0.4968010	insignificant
control vs -N-P	11.9163	0.0010053	** p<0.01
-N vs -P	2.3462	0.4968010	insignificant

treatments pair	Tukey HSD Q statistic	Tukey HSD p-value	Tukey HSD inferfence
-N vs -N-P	11.9163	0.0010053	** p<0.01
-P vs -N-P	9.5701	0.0010053	** p<0.01

ΣPUFAs (mg L^{-1})

One-way ANOVA of kk=5 independent treatments

source	sum of squares SS	degrees of freedom νν	mean square MS	F statistic	p-value
treatment	26.3724	4	6.5931	7.5193	0.0046
error	8.7683	10	0.8768		
total	35.1407	14			

Tukey HSD results

treatments pair	Tukey HSD Q statistic	Tukey HSD p-value	Tukey HSD inferfence
60 days culture vs control	3.9830	0.1039280	insignificant
60 days culture vs -N	6.0301	0.0112081	* p<0.05
60 days culture vs -P	6.9179	0.0044387	** p<0.01
60 days culture vs -N-P	2.7931	0.3426742	insignificant
control vs -N	2.0470	0.6054639	insignificant
control vs -P	2.9349	0.3009818	insignificant
control vs -N-P	1.1900	0.8999947	insignificant
-N vs -P	0.8879	0.8999947	insignificant
-N vs -N-P	3.2370	0.2252158	insignificant
-P vs -N-P	4.1249	0.0891351	insignificant

Biomass dry weight (g L^{-1})

One-way ANOVA of kk=4 independent treatments

source	sum of squares SS	degrees of freedom νν	mean square MS	F statistic	p-value
treatment	231,134.7400	3	77,044.9133	33.0222	7.4379e-05
error	18,664.9867	8	2,333.1233		
total	249,799.7267	11			

Tukey HSD results

treatments pair	Tukey HSD Q statistic	Tukey HSD p-value	Tukey HSD inferfence
control vs -N	7.2960	0.0038167	** p<0.01
control vs -P	14.0625	0.0010053	** p<0.01
control vs -N-P	6.6828	0.0064943	** p<0.01
-N vs -P	6.7665	0.0060325	** p<0.01
-N vs -N-P	0.6132	0.8999947	insignificant
-P vs -N-P	7.3797	0.0035576	** p<0.01

Total lipids (% of biomass)

One-way ANOVA of kk=4 independent treatments

source	sum of squares SS	degrees of freedom νν	mean square MS	F statistic	p-value
treatment	1,483.1633	3	494.3878	19.5012	0.0005
error	202.8133	8	25.3517		
total	1,685.9767	11			

Tukey HSD results

treatments pair	Tukey HSD Q statistic	Tukey HSD p-value	Tukey HSD inferfence
control vs -N	6.5016	0.0076361	** p<0.01
control vs -P	3.4629	0.1443727	insignificant
control vs -N-P	10.3773	0.0010053	** p<0.01

treatments pair	Tukey HSD Q statistic	Tukey HSD p-value	Tukey HSD inferfence
-N vs -P	3.0387	0.2173114	insignificant
-N vs -N-P	3.8757	0.0959773	insignificant
-P vs -N-P	6.9144	0.0052991	** p<0.01

Total lipids (mg L^{-1})

One-way ANOVA of kk=4 independent treatments

source	sum of squares SS	degrees of freedom νν	mean square MS	F statistic	p-value
treatment	185,067.1200	3	61,689.0400	48.0869	1.8356e-05
error	10,262.9200	8	1,282.8650		
total	195,330.0400	11			

Tukey HSD results

treatments pair	Tukey HSD Q statistic	Tukey HSD p-value	Tukey HSD inferfence
control vs -N	7.3311	0.0037080	** p<0.01
control vs -P	0.7060	0.8999947	insignificant
control vs -N-P	14.0207	0.0010053	** p<0.01
-N vs -P	8.0371	0.0020742	** p<0.01
-N vs -N-P	6.6896	0.0064573	** p<0.01
-P vs -N-P	14.7267	0.0010053	** p<0.01

Total fatty acids (% of biomass)

One-way ANOVA of kk=4 independent treatments

source	sum of squares SS	degrees of freedom νν	mean square MS	F statistic	p-value
treatment	1,563.5710	3	521.1903	128.4123	4.1810e-07
error	32.4698	8	4.0587		

source	sum of squares SS	degrees of freedom νν	mean square MS	F statistic	p-value
total	1,596.0408	11			

Tukey HSD results

treatments pair	Tukey HSD Q statistic	Tukey HSD p-value	Tukey HSD inferfence
control vs -N	17.6246	0.0010053	** p<0.01
control vs -P	10.9158	0.0010053	** p<0.01
control vs -N-P	26.9098	0.0010053	** p<0.01
-N vs -P	6.7088	0.0063462	** p<0.01
-N vs -N-P	9.2852	0.0010053	** p<0.01
-P vs -N-P	15.9940	0.0010053	** p<0.01

TAGs (% of biomass)

One-way ANOVA of kk=4 independent treatments

source	sum of squares SS	degrees of freedom νν	mean square MS	F statistic	p-value
treatment	73.1836	3	24.3945	105.5431	8.9864e-07
error	1.8491	8	0.2311		
total	75.0327	11			

Tukey HSD results

treatments pair	Tukey HSD Q statistic	Tukey HSD p-value	Tukey HSD inferfence
control vs -N	11.8649	0.0010053	** p<0.01
control vs -P	21.9765	0.0010053	** p<0.01
control vs -N-P	21.2560	0.0010053	** p<0.01
-N vs -P	10.1116	0.0010053	** p<0.01
-N vs -N-P	9.3911	0.0010053	** p<0.01

treatments pair	Tukey HSD Q statistic	Tukey HSD p-value	Tukey HSD inferfence
-P vs -N-P	0.7205	0.8999947	insignificant

TAGs (mg L^{-1})

One-way ANOVA of kk=4 independent treatments

source	sum of squares SS	degrees of freedom νν	mean square MS	F statistic	p-value
treatment	3,991.1446	3	1,330.3815	59.0671	8.4187e-06
error	180.1857	8	22.5232		
total	4,171.3303	11			

Tukey HSD results

treatments pair	Tukey HSD Q statistic	Tukey HSD p-value	Tukey HSD inferfence
control vs -N	6.9002	0.0053661	** p<0.01
control vs -P	8.1045	0.0019651	** p<0.01
control vs -N-P	18.6130	0.0010053	** p<0.01
-N vs -P	1.2044	0.8120709	insignificant
-N vs -N-P	11.7128	0.0010053	** p<0.01
-P vs -N-P	10.5084	0.0010053	** p<0.01

Cell number (10^{6} mL^{-1})

One-way ANOVA of kk=4 independent treatments

source	sum of squares SS	degrees of freedom νν	mean square MS	F statistic	p-value
treatment	142.1750	3	47.3917	169.4173	1.4103e-07
error	2.2379	8	0.2797		
total	144.4129	11			

Tukey HSD results

treatments pair	Tukey HSD Q statistic	Tukey HSD p-value	Tukey HSD inferfence
control vs -N	26.5152	0.0010053	** p<0.01
control vs -P	19.9765	0.0010053	** p<0.01
control vs -N-P	28.4910	0.0010053	** p<0.01
-N vs -P	6.5387	0.0073861	** p<0.01
-N vs -N-P	1.9758	0.5330124	insignificant
-P vs -N-P	8.5146	0.0014246	** p<0.01
